# Inside the Alterations of Circulating Metabolome in Antarctica: The Adaptation to Chronic Hypoxia

**DOI:** 10.3389/fphys.2022.819345

**Published:** 2022-01-25

**Authors:** Michele Dei Cas, Camillo Morano, Sara Ottolenghi, Roberto Dicasillati, Gabriella Roda, Michele Samaja, Rita Paroni

**Affiliations:** ^1^Department of Health Sciences, Università degli Studi di Milano, Milan, Italy; ^2^Department of Medicine and Surgery, Università degli Studi di Milano-Bicocca, Milan, Italy; ^3^Department of General Surgery, ASST Santi Paolo e Carlo, San Paolo Hospital, Milan, Italy; ^4^Department of Pharmaceutical Sciences, Università degli Studi di Milano, Milan, Italy; ^5^MAGI Group, Brescia, Italy

**Keywords:** chronic hypoxia, adaptation, Antarctica, metabolites, metabolomics, lipidomics

## Abstract

Although the human body may dynamically adapt to mild and brief oxygen shortages, there is a growing interest in understanding how the metabolic pathways are modified during sustained exposure to chronic hypoxia. Located at an equivalent altitude of approximately 3,800 m asl, the Concordia Station in Antarctica represents an opportunity to study the course of human adaption to mild hypoxia with reduced impact of potentially disturbing variables else than oxygen deprivation. We recruited seven healthy subjects who spent 10 months in the Concordia Station, and collected plasma samples at sea level before departure, and 90 days, 6 months, and 10 months during hypoxia. Samples were analyzed by untargeted liquid chromatography high resolution mass spectrometry to unravel how the non-polar and polar metabolomes are affected. Statistical analyses were performed by clustering the subjects into four groups according to the duration of hypoxia exposure. The non-polar metabolome revealed a modest decrease in the concentration of all the major lipid classes. By contrast, the polar metabolome showed marked alterations in several metabolic pathways, especially those related to amino acids metabolism, with a particular concern of arginine, glutamine, phenylalanine, tryptophan, and tyrosine. Remarkably, all the changes were evident since the first time point and remained unaffected by hypoxia duration (with the exception of a slight return of the non-polar metabolome after 6 months), highlighting a relative inability of the body to compensate them. Finally, we identified a few metabolic pathways that emerged as the main targets of chronic hypoxia.

## Introduction

About 83 million people permanently reside above 2,500 m asl (Beall, 2014) and numberless people are exposed to moderate or high altitudes for touristic and job-related reasons worldwide, yet sojourning at altitude still represents a challenge because of systemic hypoxia, i.e., lower oxygen supply with respect to the body’s need. Although humans can adapt, at least partially, to mild and brief oxygen shortages, hypoxia still remains a potentially lethal situation that represents a great burden in terms of lost lives and social costs for care and rehabilitation, especially for children in low- and middle-income countries (Lam et al., 2020). Nevertheless, despite a plethora of studies (10,194 articles with the term “hypoxia” in the title have been published in PubMed in 2020, compared to 5,610 in 2010 and 2,670 in 2000), systemic hypoxia has not yet been examined to a sufficient detail to grant adequate and safe therapeutic countermeasures. This issue is further aggravated by the occurrence of adaptive patterns that may potentially lead to pathological conditions.

Long-term adaptation of sea-level dwellers to hypoxia involves metabolic, respiratory, circulatory, and genetic mechanisms (Julian, 1985; Moore, 1985; Storz, 2021). Among these, the mechanisms related to respiratory adaptation are particularly suitable to monitor hypoxia adaptation. Yet the comprehension of these mechanisms requires the availability of experimental models wherein altitude hypoxia represents the unique major variable, with exclusion of poorly controllable factors, such as strenuous exercise, psychological stress, excessive temperature fluctuations, altitude changes, irregular feeding, and liquids assumption. The French-Italian Concordia Station in the Antarctica represents an opportunity to study the progress of human adaptation to hypoxia. The station is located at 3,233 m asl, but high latitude reduces the local barometric pressure and the amount of oxygen in breathed air mimics an approximate altitude of 3,800 m asl that leads to arterial oxygen saturation in the 91–94% range (Porcelli et al., 2017). As this condition is to be classified as mild hypoxia (Jahan et al., 2014), it represents a non-dangerous situation to whom healthy humans are predicted to adapt. As the subjects who winter-over at the Concordia Station do not have any possibility to change altitude for up to 10 months, this provided the chance to get an insight into the effects of mild hypoxia in the absence of disturbing factors.

In this study, we report the non-polar and polar metabolomes in plasma samples obtained from subjects exposed to mild hypoxia in the search of signs of long-term adaptation or return to baseline conditions. The non-polar and polar metabolomes were investigated by an up-to-date liquid chromatography high resolution mass spectrometry (LC-HR-MS) platform looking for metabolic pathways with impact on the human body response to hypoxia. The working hypothesis is that the absence of confounding factors and the lack of observable distressful outcomes would enable to focus into the physiological mechanisms underlying the body adaptation to mild hypoxia, a stressing condition that is more common than moderate and extreme hypoxia.

## Materials and Methods

### Subjects

Seven healthy crew members in the Concordia Station (all men, aged 25–55, mean 41 years old) participated in this study. All enrolled subjects signed a declaration whereby they were informed of the risks of the study and agreed in performing the planned measurements for the full duration of the study. This study, that was approved by the Ethical Committee of the San Paolo Hospital in Milan, was conducted within the framework of the European Space Agency’s Life Science campaign at the Concordia Station.

### Study Design

The data reported in this study were obtained during the winter-over campaign in 2019. Baseline measurements (Group 0) were performed before the start of the mission at the European Space Agency Centre in Cologne (Germany, 91 m asl). Then, subjects were studied at three time points: 90 days (Group 90) after reaching the Concordia Station, 150–180 days (Mid-Winter, Group MW), and at the end of the winter-over campaign, immediately before leaving, 300 days after their arrival (Group END). The temperature inside the Concordia Station, a French-Italian research facility located at Dome C on the Antarctic Plateau, was permanently 22 ± 2°C despite the average air temperature, humidity and wind speed outside the Station were −58 ± 9°C, 41 ± 10%, and 2.8 m/s, respectively. Located at a geographical altitude of 3,233 m asl, the mean barometric pressure during the winter-over (478 mmHg) corresponds to an altitude of approximately 3,800 m asl in the rest of the world, a value slightly lower than that reported elsewhere (Venemans-Jellema et al., 2014), probably because the oxygen fraction in the Antarctica air (20.82–20.90%) is lower than that in the rest of the world (Kanwisher, 1957). However, all subjects were examined weekly by a staff physician for assessment of the Acute Mountain Sickness (AMS) score according to the Lake Louise symptom scale (Ferrazzini et al., 1987).

At all time points, blood samples were obtained from the antecubital vein in K_2_ EDTA vacuum tubes while fasting in the morning, centrifuged (2,500 rpm, 15 min) and plasma was stored at −80°C until analyses performed in the laboratory in Milan.

### Chemicals and Reagents

The chemicals acetonitrile, 2-propanol, methanol, ethanol, chloroform, formic acid, ammonium acetate, and ammonium formate were all at analytical grade and purchased by Sigma-Aldrich (St. Louis, MO, United States). All aqueous solutions were prepared using purified water at a Milli-Q grade (Burlington, MA, United States).

### Creation of an In-House Metabolites Library

The Mass Spectrometry Metabolite Library (Supplied by IROA Technologies; Gonzalez-Ruiz et al., 2017; Pezzatti et al., 2019) was purchased by Sigma-Aldrich (St. Louis, MO, United States) and includes 603 unique metabolites divided in seven plates: five with polar metabolites and two with lipid-related molecules (for the composition of the plates, see Supplementary Table S1). Stock solutions of the analytes were prepared at the concentration of 25 μg/ml according to the manufacturer’s instructions by using 5% methanol in water for plates 1–5 and methanol/chloroform (1:1, v/v) for lipids. Each plate was shaken on an oscillator thermo-mixer for 30 min at 1,000 rpm at 5°C. Twenty-nine pools were prepared, so that each one contained a maximum of 22 standards with distinct exact masses; isomers were never mixed in the same vial. The different mixes’ final concentration was about 1.25 μg/ml (for the composition of each pool, see Supplementary Table S2). Five μl of each vial was directly injected in LC–MS/MS (see “Analysis by LC-HR-MS”).

After LC–MS analysis of the standard pools, data were manually processed by MS-DIAL (ver 4.0) or semi-automatically by MSLDiscovery (ver. 3.1B.15), checking for the most prominent adducts for each analyte in both polarities (Simon-Manso et al., 2013; see “LC-HR-MS Data Processing”). The retention time (Rt), intensity, three prevalent MS/MS fragments, and S/N for each standard peak were annotated only when at least one of the accurate masses of the adducts was in accordance with those reported in MSLDiscovery Database (m/z ± 0.02 Da, <30 ppm). Final curation of the “in-house” attained Database, and compounds validation, was then made on the basis of isotopic distribution, the number of recognized adducts, and MS/MS spectra. The in-house Database comprised at the end 506 metabolites (see Supplementary Table S3).

### Plasma Extraction Procedures

#### Non-polar Metabolites

Plasma (25 μl) was diluted with water (75 μl) and added with cold methanol/chloroform mixture (850 μl, 2:1, v/v). They were ice-sonicated and extracted with an oscillator thermo-mixer (30 min 5°C, 1,000 rpm). After centrifugation (15 min at 13,400 rpm), the organic phase was evaporated under a stream of nitrogen. The residues were dissolved in 100 μl of isopropanol/acetonitrile (2:1, v/v) + 0.1 mm BHT and withdrawn in a glass vial (Dei Cas et al., 2020).

#### Polar Metabolites

Plasma (50 μl) was diluted with water (50 μl) and added with cold methanol/ethanol mixture (400 μl, 1:1, v/v). They were ice-sonicated and extracted with an oscillator thermo-mixer (30 min 5°C, 1,000 rpm). After centrifugation (15 min at 13,400 rpm), the protein debris was discharged, and the clean supernatant was evaporated under a stream of nitrogen. The residues were dissolved in 50 μl of water and withdrawn in a vial (Wawrzyniak et al., 2018).

### Analysis by LC-HR-MS

The instrument consisted of a Shimadzu UPLC coupled with a Triple TOF 6600 Sciex (Concord, Canada) equipped with Turbo Spray IonDrive. All samples were analyzed in duplicate in both positive and negative mode with electrospray ionization.

#### Untargeted Lipidomics

The instrument settings were as follows: CUR = 35, GS1 = 55, GS2 = 65, capillary voltage ±5.5 kV, and source temperature 350°C. Spectra were contemporarily acquired by full-mass scan from *m/z* 200 to 1,500 (100 ms accumulation time) and data-dependent acquisition from *m/z* 50 to 1,500 (40 ms accumulation time, top-20 spectra per cycle 0.8 s). Declustering potential (DP) was fixed to 50 eV, and the collision energy (CE) was 35 ± 15 eV. The chromatographic separation was reached on a reverse-phase Acquity CSH C18 column 1.7 μm, 2.1 × 100 mm (Waters, MA, United States) equipped with a pre-column by using, as mobile phase (A) water/acetonitrile (60:40) and, as mobile phase (B) 2-propanol/acetonitrile (90:10), both containing 10-mm ammonium acetate and 0.1% of formic acid (Dei Cas et al., 2020). The flow rate was 0.4 ml/min, and the column temperature was 55°C. The elution gradient (%B) was set as below: 0–2.0 min (40%), 2.0–2.5 min (40–50%), 2.5–12.5 min (50–55%), 12.5–13.0 min (55–70%), 13.0–19.0 min (70–99%), 19.0–24.0 min (99%), and 24.0–24.2 (99–40%) and kept constant until 30 min. Five μl of clear organic supernatant was directly injected in the LC–MS/MS.

#### Untargeted Metabolomics

The instrument settings were as follows: CUR = 35, GS1 = 40, GS2 = 40, capillary voltage ±5.5 kV, and source temperature 500°C. Spectra were contemporarily acquired by full-mass scan from *m/z* 50 to 1,000 (100 ms accumulation time) and data-dependent acquisition from *m/z* 40 to 1,000 (40 ms accumulation time, top-20 spectra per cycle 0.8 s). Declustering potential (DP) was fixed to 60 eV, and collision energy (CE) was 30 ± 15 eV. Chromatographic separation was achieved on a reverse-phase Acquity HSS T3 column 1.7 μm, 2.1 × 100 mm (Waters, MA, United States) equipped with pre-column using as mobile phase (A) water and as mobile phase (B) methanol both containing 0.1% of formic acid (Want et al., 2013). The flow rate was 0.4 ml/min and the column temperature was 40°C. The elution gradient (%B) was set as below: 0–2.0 min (1%), 2.0–6.0 min (1–25%), 6.0–10.0 min (25–80%), 10.0–12.0 min (80–90%), 12.0–21.0 min (90–99%), 21.0–23.0 min (99–99%), and 23.0–23.2 min (99–1%) held until 30 min. Five μl of clear aqueous supernatant was directly injected in LC–MS/MS.

#### LC-HR-MS Data Processing

The spectra deconvolution, peak alignment, and sample normalization were attained using MS-DIAL (ver. 4.0; Tsugawa et al., 2015; Cajka et al., 2017; Huan et al., 2017). MS and MS/MS tolerance for peak profile were set to 0.01 and 0.05 Da, respectively. Identification was achieved matching molecular (*m/z* ± 0.02) and MS/MS experimental spectra (*m/z* ± 0.05) with (Beall, 2014) the Fiehn Hilic library for metabolomics or (Lam et al., 2020) the LipidBlast library for lipidomics. MS-DIAL post-identification was completed by the in-house library, which also considered analytes retention times. Features that met the following criteria were extracted and used for further analysis: (Beall, 2014) the CV% of the feature in the QC sample, repeatedly injected all along with the batch, should be <30%, and (Lam et al., 2020) the value of the feature peak was more than 10-fold the value of the same feature in the blank. Intensities of the remained metabolites were normalized by Lowess algorithm.

### Statistical Analysis

Multivariate analysis was achieved with MetaboAnalyst 5.0, after data log-transformation and auto-scaling. Partial least squares discriminant analysis (PLS-DA) was performed to increase the group separation and to identify the variables with high Variance Importance in Projection score (VIP), assuming a cut-off value of 1.0 to disclose the discriminating variables. In PLS-DA, the dimension of a dataset is reduced while retaining as much information as possible; particularly, all the data acquired from a sample are condensed in a single dot, characterized by two dimensions. To further corroborate data, univariate statistical analysis was performed by GraphPad Prism 9.0 (GraphPad Software, Inc., La Jolla, California, United States) using one-way ANOVA followed by the Dunnett *post-hoc* test to compare metabolites against baseline (time 0) for each subject.

To confirm the relevance of the identified metabolites in precise metabolic pathways affected by hypoxia, the pathway analysis was performed based on *Homo sapiens* KEGG pathway networks (release 99, 2021/07). The value of *p* was corrected for the false discovery rate. Visualization of the results was attained using GraphPad Prism 9.0 (GraphPad Software, Inc., La Jolla, California, United States). In all tests, *p* < 0.05 was considered statistically significant.

## Results

The environmental conditions at the Concordia Station were predicted to cause some degree of emotional stress (Caputo et al., 2020), but all subjects remained in good health for the whole duration of the study and never experienced signs of high-altitude illness (AMS score < 1).

### Non-polar Metabolome

**Figure 1:**
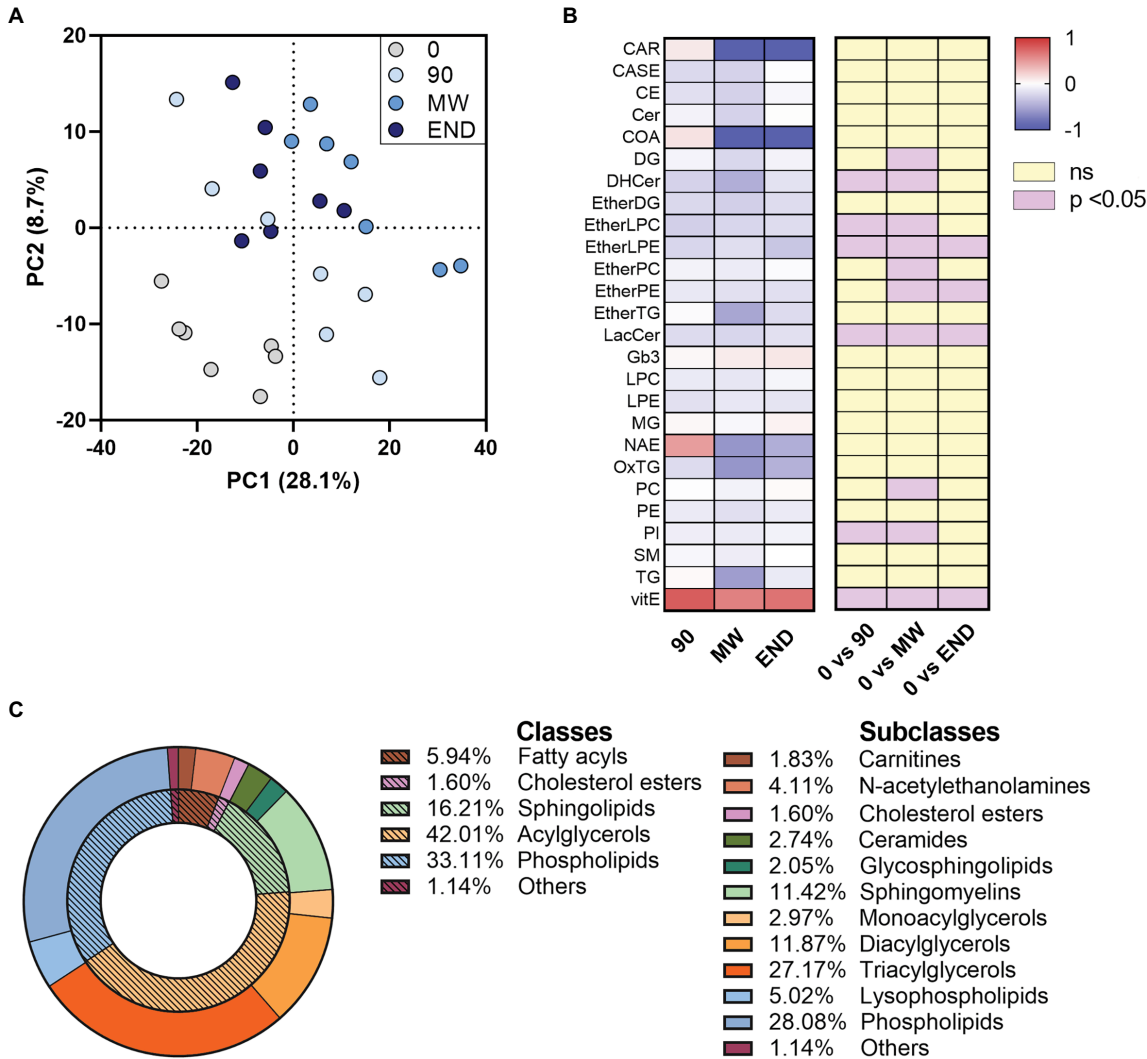
Alterations in circulating lipidome during 10 months of chronic hypoxia in the Antarctica. **(A)** Multivariate analysis visualized as principal discriminant analysis of lipids in plasma after 0, 90, 150–180 (MW), and 300 days (END) in the Antarctica. **(B)** Discriminant lipids were chosen between those with a VIP > 1.0, then ordered and summed according to their class, and finally visualized as a heat map after transformation to values of *z*. Data are shown as log-fold change of each time points (90, MW, END) over baseline values. The three columns on the right show the significance of the difference vs. time 0 by one-way ANOVA and the Dunnett *post-hoc* test. **(C)** Discriminant (VIP) lipids grouped in their classes and subclasses as a donut chart graph.

By processing the LC-HR-MS data as described in “LC-HR-MS Data Processing”, we identified ~1,000 lipid species in the plasma of each subject. The discriminant analysis (PLS-DA), useful to highlight the changes in the lipidome along the chronic hypoxia exposure period, showed a separation of 28.1% on principal component (PC1). The analysis showed a remarkable separation between the lipidome gathered at sea level before departure (time 0), and all the other observation times during hypoxia (Figure 1A).

To rank the discriminating features alongside the exposure to hypoxia, we used the VIP scores derived from the PLS-DA, assuming 1.0 as cut-off value. With this process, we selected 220 discriminant lipid species with VIP > 1.0 that emerged as markers of the differences among the plasma samples gathered at the four time points. They were then ordered and grouped according to their classes and visualized as a heat map after transformation to values of *z* (Figures 1B,C and Supplementary Figure S1). To detect those metabolites that were changed (*p* < 0.05) in at least one time point with respect to baseline, we employed univariate ANOVA. Apart from the 90-day time point, which often showed mild non-significant changes in some lipid species, the trend depicted a decrease in nine plasma lipid species along with hypoxia time, while Vitamin E levels resulted increased (Figure 1B). Although the data points taken in hypoxia appear consistently different from baseline at any time, a slight but significant (*p* < 0.01) change was observed between MW and END along PC1, highlighting a possible normalization of the non-polar metabolome after 6-month hypoxia. Such change, however, was not observed along neither PC2 nor PC3.

### Polar Metabolome

**Figure 2:**
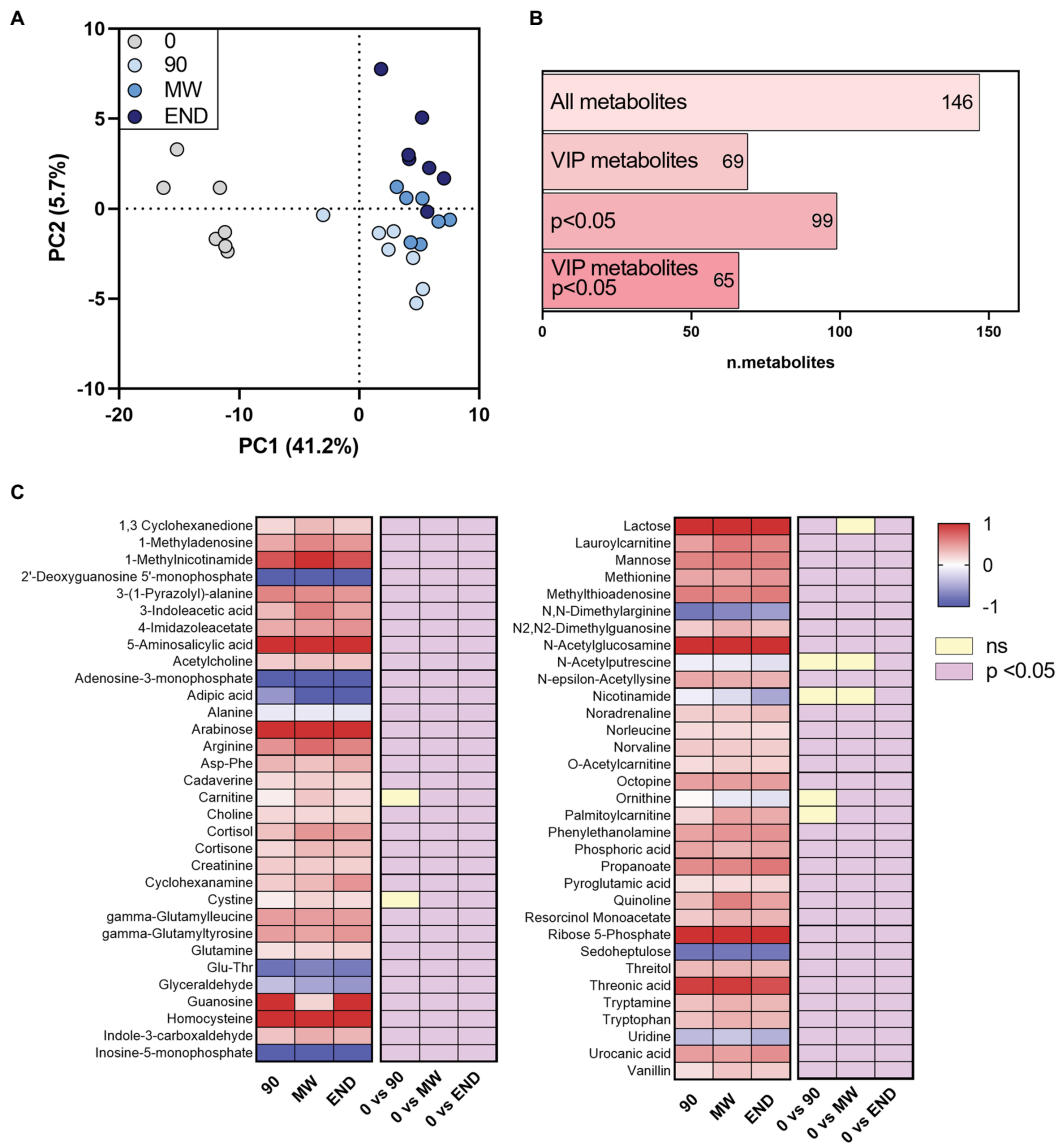
Alterations in the circulating polar metabolome during 10 months of chronic hypoxia in the Antarctica. **(A)** Multivariate analysis visualized as principal discriminant analysis of polar metabolites in plasma after 0, 90, 150–180 (MW), and 300 days (END) in Antarctica. **(B)** Number of identified metabolites in each subgroup: VIP, metabolites with *p* < 0.05 and VIP with *p* < 0.05. **(C)** Heat map of discriminant metabolites with VIP > 1.0, and at least one time point with *p* < 0.05 vs. time 0 after transformation to values of *z*. Data are shown as log-fold change of each time points (90, MW, END) over baseline. The three columns on the right show the significance of the difference vs. time 0 by one-way ANOVA and the Dunnett *post-hoc* test.

After applying to LC-HR-MS data the same processing procedure described in paragraph “LC-HR-MS Data Processing”, we identified *n* = 146 polar metabolites in the plasma of each subject (Supplementary Table S4). PLS-DA on polar metabolites showed a more remarkable separation than lipids between non-hypoxic and hypoxic conditions (PC1 = 41.2% vs. 28.3%; Figure 2A). The PLS-DA performed on these metabolites helped identifying *n* = 69 metabolites with VIP > 1 that are related with hypoxia (Figure 2B). Figure 2C shows as the heat map of the metabolites that satisfy two conditions: VIP > 1 and *p* < 0.05 at least at one time point during the observation period (*n* = 65). Whereas PLS-DA component 1 describes the immediate responses to environment changes, component 2 shows the progressive change of the polar metabolome during hypoxia exposure. The VIP metabolites calculated on both PC1 and PC2 are listed in Supplementary Table S5. Particularly, the results from this analysis appear consistent with the previous ones, as most of the metabolites are found in both VIP groups, such as ornithine, nicotinamide, and N-acetylputrescine. By contrast, other molecules, such as, for instance, propionylcarnitine, are VIP metabolites following PC2 only.

#### Pathway Analysis

**Figure 3:**
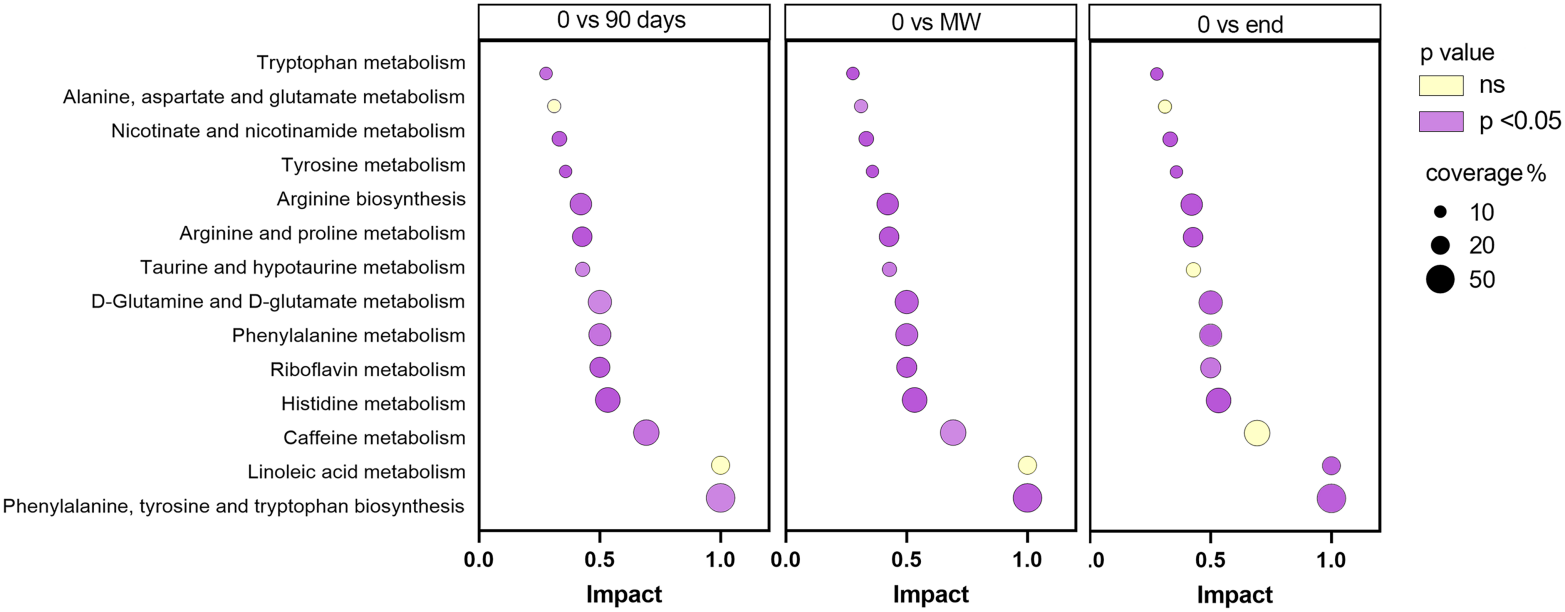
Schematic diagram of the most altered metabolic pathways after 90 days, MW and 9 months in Antarctica with respect to baseline. Values of *p* were corrected for false discovery rate. Enrichment pathway analysis was performed based on *Homo sapiens* KEGG pathway networks. In purple, are reported those significantly modulated by performing one-way ANOVA and the Dunnett *post-hoc* test; in yellow, those not modulated. For each metabolism pathway, the coverage is represented by the size of the circles, while the position on the X-axis indicates the impact in a scale from 0 to 1.

**Figure 4:**
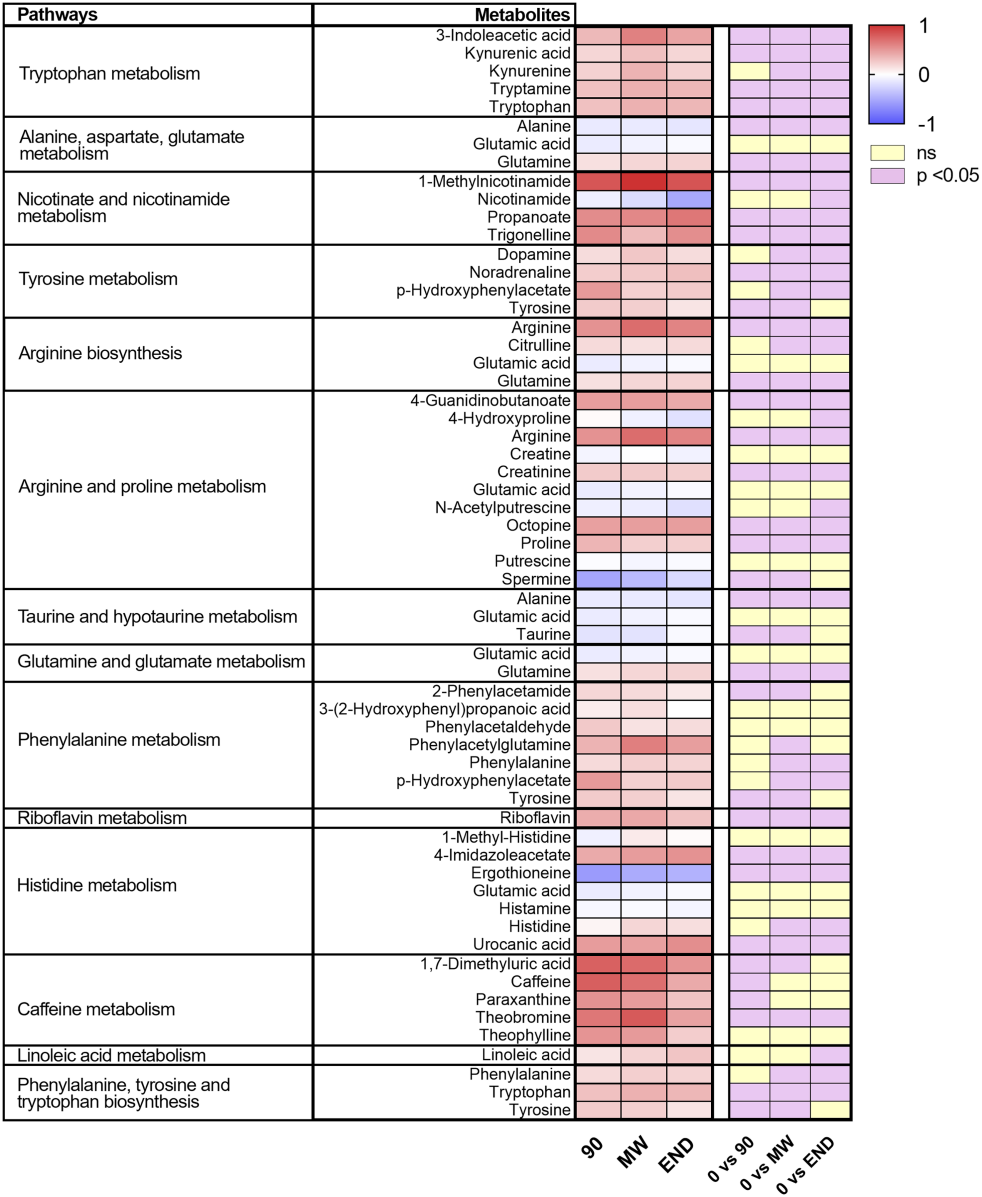
Heat map of the metabolites included in the pathways significantly affected by hypoxia for every time point. On the right, are indicated in violet those significantly modulated by performing one-way ANOVA and the Dunnett *post-hoc* test; in yellow, those not modulated.

To discover the metabolic pathways mostly involved during hypoxia adaptation, we performed three different pathway analyses comparing the metabolomes of the three time points during hypoxia with the baseline one. The bubble graph in Figure 3 shows schematically the modulation of the pathways. For each metabolism pathway, the coverage is represented by the size of the circles, while the position on the X-axis indicates the impact in a scale from 0 to 1 and the bubble color the value of *p*. All the pathways indicated therein have an impact of at least 0.3. Most of the pathways implicated in chronic hypoxia seem to be linked to the metabolism of amino acids. More specifically, the pathways with higher impact and coverage resulted to be associated to the biosynthesis of phenylalanine, tyrosine, and tryptophan (map 00040 in KEGG). Figure 4 displays, instead, the relation of each metabolite to the modulated pathway itself and their levels across the groups. For each of the pathways represented as a bubble in Figure 3, all the detected metabolites are annotated from the *H. sapiens* KEGG pathway networks, their variation over time and the significance of the variation.

## Discussion

When the effects of chronic hypoxia in humans are investigated at altitude, many disturbing factors may interfere with the measurement of metabolic and physiological variables. These may include exposure to cold, development of anxiety, heavy exercise, abrupt altitude changes, environmental-born stress, food ingestion irregularities, or even starving. By contrast, the logistics of the Concordia Station, with reduced fluctuations in temperature and humidity, comforting environment, regular feeding, and drinking, impossibility to change altitude and relatively controlled psychological stress should reduce, or even prevent, the occurrence of disturbing factors. Specifically, although cold exposure during outdoor activities may affect the lipidome profile (Lynes et al., 2018; Coolbaugh et al., 2019), we exclude this as a potential confounding factor in this study, due to the low exposure volume and the great preventive attention paid by the subjects. It is known that the lack of natural sunlight may alter the fasting plasma metabolome in healthy subjects (Noordam et al., 2019). Therefore, it is possible that the rupture in the circadian rhythms in the subjects studied here may represent a confounding factor because the MW time point corresponds to day-long lightless conditions in contrast to the 90 and END points. Although indoor light tended to preserve in part the day-night light cycle, this confounding factor should be taken as a potential limit of this study.

Therefore, in this study, hypoxia may be considered the only variable that influenced the measurements. The duration of the subjects’ sojourn at altitude (10 months) enables focusing into relatively long-term adaptation to hypoxia. The degree of hypoxia at the Concordia Station, equivalent to approximately 3,800 m asl, is compatible with the body’s need to adapt, yet it is not extreme, with the risk to conflict with altitude sickness. Indeed, the blood gas values at the Concordia Station revealed decreased pCO_2_ with alkalemia resulting from moderate increase of the breathing rate to balance hypoxia (Porcelli et al., 2017). The inadequate renal compensation to cope with increased CO_2_ washout in the lungs nevertheless produced a persistent base excess of −4 mEq/l, to be compared with −6 mEq/l found at 6,450 m asl (Samaja et al., 1997).

Today LC-HR-MS represents a powerful approach to address the metabolome changes in response to hypoxia. Despite the low number of subjects, most of the metabolome changes were statistically consistent and visible since *t* = 90 days. Because they remained independent of the hypoxia duration until *t* = 300 days, this highlights human inability to compensate the observed changes with return to baseline values. This feature might be interpreted as lack of adaptation to hypoxia, in the same way as it has been previously observed as for the changes induced by hypoxia on the acid–base status and on the erythropoietic stimulus (Porcelli et al., 2017).

Whereas some of the observed changes appear occasional and perhaps dependent on the individual diet (e.g., caffeine, 1,7-dimethyluric acid, and Vitamin E), other changes may be referred to as part of one or more mechanisms of hypoxia adaptation, with special concern of the metabolism of amino acids. Exposure to altitude hypoxia is well known to increase protein catabolism (Pasiakos et al., 2017). However, the finding that the whole pool of proteogenic amino acid is not altered by hypoxia nor to vary with time (Supplementary Figure S2) suggests the lack of significant hypoxia-induced protein catabolism. Most likely, the living conditions at the Concordia Station exclude this potentially confounding variable (Rathor et al., 2021); thus, the variations of individual amino acids may be attributed solely to selective recruitment of hypoxia-linked metabolic pathways (see also Supplementary Figure S3).

The increase in the plasma levels of some amino acids agrees with a study performed in 34 volunteers ascending progressively to 6,885 m asl in 19 days, that showed altitude-related increases in tryptophan, serotonin, and peroxidation-sensitive lipids, together with enhanced oxidative stress (Pichler Hefti et al., 2013). In an attempt to associate the variations of single amino acids to hypoxia-linked pathways, we focused into those species that displayed marked (>0.5) and significant (*p* < 0.05) changes over the three time points.

High glutamine may be related to the disruption of the ammonia vs. glutamate balance with potential to affect negatively the brain cognitive function (Dos Santos et al., 2020), while depleted levels of the excitatory neurotransmitter glutamate may be associated to increased brain uptake of glutamate to stimulate ventilation in an attempt to compensate lower oxygen saturation (Honda et al., 1985). The disruption of the ammonia vs. glutamate balance is likely related to altered nitrogen metabolism and supported by the decreases in N-acetylputrescine, spermine, and ornithine, polyamines with relevant physiological roles on angiogenesis and reproductive physiology (Lenis et al., 2017; Supplementary Figure S4).

Higher plasma tryptophan may highlight lower activity of the tryptophan hydroxylase reaction, the rate-limiting enzyme in serotonin synthesis because it requires molecular oxygen (Fitzpatrick, 1999). Enhanced hypoxia vulnerability of this enzyme (Vaccari et al., 1978) is expected to lower brain serotonin synthesis, which results in depression, reduced appetite and motivation, and disruption of sleep patterns (Katz, 1982), as commonly found in altitude residents (Maa, 2010; DelMastro et al., 2011). Likewise, higher tryptophan may also recruit the kynurenine pathway. Untargeted metabolomic analysis already revealed that these pathways are upregulated in a model of perinatal asphyxia and neonatal hypoxia-ischemia encephalopathy (Denihan et al., 2019). Furthermore, enhanced activity of the kynurenine pathway is a common finding in umbilical cord blood and placental samples in normal and fetal growth restriction pregnancies, as well as in the media of placental explants incubated with 5–8% oxygen (Murthi et al., 2017).

The kynurenine pathway is also linked to the nicotinamide pathway to account for the increased levels of 1-methylnicotinamide observed in this study. The observed decrease in nicotinamide may be related to its higher use in the nicotinamide phosphoribosyltransferase reaction that promotes pulmonary vascular remodeling in the development of pulmonary arterial hypertension (Chen et al., 2017), a common finding in chronic hypoxia (Ottolenghi et al., 2020; Gassmann et al., 2021). As a matter of facts, either nicotinamide (Sztormowska-Achranowicz et al., 2020) or niacin (or nicotinic acid; Jia et al., 2020) are being investigated as drugs to ameliorate progression of hypoxia-induced pulmonary hypertension.

A precursor of NO, arginine is often used as a vasodilator drug in several human diseases, but the effect of hypoxia on NO bioavailability is still debated. In a most recent view, improved NO bioavailability appears to favor human adaptation to altitude (Beall et al., 2012) and underlies the Tibetans’ response to altitude hypoxia (Erzurum et al., 2007). This is compatible with either the increased plasma arginine observed in this study, and the beneficial effects of supplementing arginine (Schneider et al., 2001) or sildenafil (Xu et al., 2014) at altitude.

Finally, octopin, a derivative of alanine and arginine that may function as an analog of lactic acid in invertebrate muscles (Hochachka et al., 1977), was found to be increased in marine animals exposed to 50% oxygen shortage without changes in protein turnover (Capaz et al., 2017).

Comparing the reported findings with the literature -*omics* data may be of limited value due to extremely different materials and hypoxia administration protocols. A first cohort of studies concerns LC-HR-MS analyses performed in contexts related to acute hypoxia. A study aimed at identifying plasma metabolites predictive of AMS insurgence in 60 subjects after 4 days at 5,300 m asl revealed variations in up to 44 metabolites of the pathways related to inflammation, energy consumption (e.g., TCA, fatty acids, and amino acids), bile acids, and hemoglobin metabolism (Liao et al., 2016). Another study of the red blood cell metabolome after 1–16 days at 5,000 m asl highlighted promoted glycolysis and deregulated pentose phosphate pathway, purine catabolism, glutathione homeostasis, arginine/NO, and sulfur/H_2_S (D’Alessandro et al., 2016). A third study performed in four subjects exposed for 2 days at 4,559 m asl revealed subject-dependent redox-changes (Cumpstey et al., 2019). Finally, a study in eight subjects exercising in a hypobaric chamber at an equivalent altitude of 4,300 m asl for 8 h revealed increased glycolysis and TCA cycle activity, amino acid breakdown, oxidative stress, fatty acid storage, and decreased fatty acid mobilization (Margolis et al., 2021). The last findings are compatible with acute onset of hypoxia-mediated peripheral insulin resistance, which is to be compared with the increased levels of some amino acids observed in the present report, along with depressed pool of lipids, cofactors, and vitamins. Thus, despite some analogies, comparing the data reported here with those obtained in acute hypoxia appears difficult.

Another cohort of -*omics* studies compared gene analysis of altitude vs. sea-level populations in the search of changes in the evolutionary processes that have tinkered differently on the various altitude populations across the world, who followed different routes of hypoxia adaptation (Beall, 2007). Such gene changes are expected to impact the metabolome. The genic differences between Andeans and Tibetans, presumably a paradigm of human generational hypoxia adaptation, as from the occurrence of Chronic Mountain Sickness, highlighted a panel of approximately 120 genes that responded differentially to hypoxia and are related to the development of the nervous, lymphoid, cardiovascular, and erythropoietic systems (Azad et al., 2017). Likewise, a gene expression study in acclimatized altitude populations revealed reduced oxidative capacity, decreased oxidation of fatty acids, and decreased ATP turnover with respect to lowlanders (Murray et al., 2018). Other genome-wide scans of high-altitude populations identified the oxygen-sensing machinery as responsible for phenotypic adjustments leading to adaptation (Storz, 2021). Certainly, genetic signals eventually impact differentially the metabolic adaptation to hypoxia but as a matter of facts studies performed in populations residing at altitude could not yet distinguish the roles played by genes, Darwinian evolution and environmental factors.

A further cohort of studies potentially useful to compare the reported data concerns the -*omics* in patients affected by pathological hypoxia secondary to pulmonary diseases as Acute respiratory distress syndrome (ARDS), Chronic obstructive pulmonary disease, and perhaps asthma. NMR-based metabolome in ARDS patients grouped by disease severity identified major involvement of amino acid-linked pathways, especially glutamate, threonine, taurine, lysine, arginine, and proline (Viswan et al., 2017). Other studies in asthma patients reviewed in (Kelly et al., 2017) revealed a wide range of affected variables. A recent review extended to all -*omics* techniques confirmed these features (Sim et al., 2021). It is therefore likely that the pulmonary pathologies that lead to chronic hypoxemia involve more pathways than those triggered by physiological hypoxia only, e.g., inflammation, iron handling dysfunction, and redox imbalance (Duca et al., 2021), thereby making it difficult to compare pathological with physiological hypoxia.

### Limits of the Study

Although data reported here were obtained under conditions of month-long hypoxia of mild intensity, it remains to be clarified whether longer or more severe challenges would enable better insight into the mechanisms underlying human adaptation to hypoxia. However, the present study, which compares the same subjects at sea level before departure and at three time points during hypoxia, may help to enucleate the effects of hypoxia as a single factor, keeping to a minimum the incidence of hypoxia-associated disturbing variables.

In the present investigation, we identified a panel of polar and non-polar metabolites that are systematically altered by chronic hypoxia. Yet, despite the statistical significance obtained using the most conservative tests, the size limit of the recruited population necessarily prevents expanding these findings to the general population facing a mild hypoxia challenge. Furthermore, the existing literature is not yet sufficiently developed to determine whether the changes described in the present report represent spurious findings or reflect the substantial heterogeneity and limited statistical power. Therefore, this study necessarily represents a preliminary report that needs validation from further larger size studies, possibly including gene correlations. Further studies should use standardized methods and – perhaps most important – reduce to a reasonable minimum the population’s background noise, i.e., not simply the usual macroscopic variables as smoking, alcohol/drug assumption, and diseases, but also abrupt changes in altitude, environment temperature/humidity, activity, and feeding and life behavior.

Both the polar and the non-polar metabolomes were characterized by relevant adjustments within 90-day hypoxia. While the polar metabolome did not change further until at least 300-day hypoxia, the non-polar metabolome apparently displayed a slight but statistically significant return toward the baseline situation after MW to be evaluated in future studies. Nevertheless, these observations highlight the completion of the metabolome response within 90-day hypoxia, accompanied by the human body’s inability to perform longer term adjustments.

Finally, the rupture of the circadian rhythms due to extremely variable light conditions during the year may be a point of concern to be examined in further studies. Likewise, even the contribute of a regular exercise program, which was not accounted for in the present investigation, to both the polar and non-polar metabolome changes, does merit future *ad-hoc* studies.

## Conclusion

The analysis of non-polar and polar metabolome patterns in the plasma from humans exposed to mild hypoxia (barometric pressure of 478 mmHg, equivalent to 3,800 m asl) revealed minor changes in the lipidome as compared to marked changes in the polar metabolite pattern. Remarkably, such changes occur within 90 days after the start of hypoxia and were constant over time for at least 300 days (with the exception of a slight return of the non-polar metabolome after 6 months), thus highlighting either a relatively acute response or the inability of the human body to further adjust the metabolism after the initial phase. KEGG analysis enabled identifying, among the pathways affected by hypoxia, the metabolism of some amino acids, chiefly arginine, glutamine, phenylalanine, tryptophan, and tyrosine, as major candidates that presumably drive the human response, perhaps adaptation, to a potentially lethal situation that challenge an intolerably high number of patients affected by pulmonary and circulatory diseases worldwide.

## Data Availability Statement

The original contributions presented in the study are included in the article/Supplementary Material, further inquiries can be directed to the corresponding author.

## Ethics Statement

The studies involving human participants were reviewed and approved by Ethical Committee of the San Paolo Hospital in Milan (154/ST/2014). The patients/participants provided their written informed consent to participate in this study.

## Author Contributions

All authors contributed extensively to the work presented in this manuscript and approved the submitted version. MD, RP, SO, and MS: conceptualization. MD and CM: investigation and formal analysis. MD and SO: visualization. RD, GR, SO, and MS: resources. RD, GR, MS, and RP: supervision. MD, SO, and CM: writing—original draft. RP, MS, and SO: writing—review and editing.

## Funding

This research was funded by the Department of Health Science, University of Milan, Italy, and the Concorde Project PNRA18_0 0071_Prot. 20891.21-11-2019 by the Ministero dell’Università e della Ricerca, Rome, Italy, assigned to MS. PNRA16_00047 project-Line A2, assigned to Dr. Simone Macri, Istituto Superiore di Sanità, Rome.

## Conflict of Interest

The authors declare that the research was conducted in the absence of any commercial or financial relationships that could be construed as a potential conflict of interest.

## Publisher’s Note

All claims expressed in this article are solely those of the authors and do not necessarily represent those of their affiliated organizations, or those of the publisher, the editors and the reviewers. Any product that may be evaluated in this article, or claim that may be made by its manufacturer, is not guaranteed or endorsed by the publisher.

## Abbreviations

AMS: Acute Mountain Sickness
Car: Carnitines
CASE: Campesterol Esters
CE: Cholesterol Esters
Cer: Ceramides
COA: Coenzyme A
DHCer: Dihydro Ceramides
DG: Diacylglycerols
EtherDG: Ether-linked Diacylglycerols
EtherLPC: Ether-linked Lysophosphatidylcholines
EtherLPE: Ether-linked Lysophosphatidylethanolamines
EtherPC: Ether-linked Phosphatidylcholines
EtherPE: Ether-linked Phosphatidylethanolamines
EtherTG: Ether-linked Triacylglycerols
LacCer: Lactosyl-Ceramides
Gb3: Globosides
LPC: Lysophosphatidylcholines
LPE: Lysophosphatidylethanolamines
MG: Monoacylglycerols
NAE: N-acylethanolamines
OxTG: Oxidized Triacylglycerols
PC: Phosphatidylcholines
PE: Phosphatidylethanolamines
PI: Phosphatidylinositols
PLS-DA: Partial Least Squares Discriminant Analysis
SM: Sphingomyelins
TG: Triacylglycerols
vitE: Tocopherols
