# Corrigendum: Inside the Alterations of Circulating Metabolome in Antarctica: The Adaptation to Chronic Hypoxia

**DOI:** 10.3389/fphys.2022.876623

**Published:** 2022-03-09

**Authors:** Michele Dei Cas, Camillo Morano, Sara Ottolenghi, Roberto Dicasillati, Gabriella Roda, Michele Samaja, Rita Paroni

**Affiliations:** ^1^Department of Health Sciences, Università degli Studi di Milano, Milan, Italy; ^2^Department of Medicine and Surgery, Università degli Studi di Milano-Bicocca, Milan, Italy; ^3^Department of General Surgery, ASST Santi Paolo e Carlo, San Paolo Hospital, Milan, Italy; ^4^Department of Pharmaceutical Sciences, Università degli Studi di Milano, Milan, Italy; ^5^MAGI Group, Brescia, Italy

**Keywords:** chronic hypoxia, adaptation, Antarctica, metabolites, metabolomics, lipidomics

In the original article, we neglected to include the funder PNRA16_00047 project - Line A2, assigned to dr. Simone Macri, Istituto Superiore di Sanità, Rome.

In the original article, we neglected to include the acknowledgment to Prof. Michele Maffia, University of Salento, Lecce, Italy, and Dr. Maria Rosaria Coscia, Institute of Biochemistry and Cell Biology, National Research Council, Naples, Italy, for kindly providing the samples used for the reported analyses.

The authors apologize for this error and state that this does not change the scientific conclusions of the article in any way. The original article has been updated.

## Publisher's Note

All claims expressed in this article are solely those of the authors and do not necessarily represent those of their affiliated organizations, or those of the publisher, the editors and the reviewers. Any product that may be evaluated in this article, or claim that may be made by its manufacturer, is not guaranteed or endorsed by the publisher.

