# Supplementary material for: Inside the Alterations of Circulating Metabolome in Antarctica: The Adaptation to Chronic Hypoxia
Source: Front Physiol. 2022 Jan 25;13:819345. doi: 10.3389/fphys.2022.819345 (PMC8821919; doi:10.3389/fphys.2022.819345)
Supplement: Supplementary file 1 [file Data_Sheet_1.PDF]

**Supplementary Materials**

# Inside the alterations of circulating metabolome in Antarctica: the (mal)adaptation to chronic hypoxia

**Michele Dei Cas[1], Camillo Morano[1], Sara Ottolenghi[1,2], Roberto Dicasillati[3], Gabriella Roda[4], Michele Samaja[1,5,#, *], Rita Paroni[1,#]**

[1] Department of Health Sciences, Università degli Studi di Milano, Milan, Italy
[2] Department of Medicine and Surgery, Università degli Studi di Milano-Bicocca, Milano, Italy
[3] Department of General Surgery, ASST Santi Paolo e Carlo, H San Paolo, Milan, Italy
[4] Department of Pharmaceutical Sciences, Università degli Studi di Milano, Milan, Italy
[5] MAGI GROUP, San Felice del Benaco, Brescia, Italy

*** Correspondence:**
Prof. Michele Samaja, Department of Health Sciences, Università degli Studi di Milano, via A di Rudinì, 8, H San Paolo, Milan, Italy. email:michele.samaja@unimi.it

#These authors equally contributed to the production of this work

**Keywords: Chronic hypoxia, Adaptation, Metabolites, Metabolomics, Antarctica**

**Supplementary Materials**

**Table S1**. The plate map of mass spectrometry metabolite Library supplied by IROA Technologies, each well contains 5 ug of each metabolite.

| PLATE | ROW | COL | PRIMARY NAME |
|---|---|---|---|
| 1 | B | 1 | 3-Methyl-L-Histidine |
| 1 | B | 2 | Nicotinamide Mononucleotide |
| 1 | B | 3 | Folate |
| 1 | B | 4 | Deoxyadenosine Monophosphate |
| 1 | B | 5 | Pyridoxine |
| 1 | B | 6 | Homoserine |
| 1 | B | 7 | Guanine |
| 1 | B | 8 | Valine |
| 1 | B | 9 | Asparagine |
| 1 | B | 10 | Glycerol |
| 1 | B | 11 | Tyrosine |
| 1 | B | 12 | Isocitrate |
| 1 | C | 1 | Malate |
| 1 | C | 2 | Dihydrouracil |
| 1 | C | 3 | Guanosine |
| 1 | C | 4 | L-Dopa |
| 1 | C | 5 | Creatine |
| 1 | C | 6 | Hypoxanthine |
| 1 | C | 7 | Deoxycytidine Monophosphate |
| 1 | C | 8 | Aspartate |
| 1 | C | 9 | Thiourea |
| 1 | C | 10 | Uracil |
| 1 | C | 11 | Phenylalanine |
| 1 | C | 12 | Succinate |
| 1 | D | 1 | Shikimate |
| 1 | D | 2 | Nicotinamide |
| 1 | D | 3 | Carnosine |
| 1 | D | 4 | Fructose Bisphosphate |
| 1 | D | 5 | Uridine |
| 1 | D | 6 | Lactate |
| 1 | D | 7 | Succinate Semialdehyde |
| 1 | D | 8 | Thymine |
| 1 | D | 9 | Proline |
| 1 | D | 10 | Uridine Monophosphate |
| 1 | D | 11 | Diethanolamine |
| 1 | D | 12 | L-Alanine |
| 1 | E | 1 | Cystine |
| 1 | E | 2 | Dihydrofolate |
| 1 | E | 3 | Xanthine |
| 1 | E | 4 | Gulose |
| 1 | E | 5 | Aminoisobutanoate |
| 1 | E | 6 | Cys-Gly |
| 1 | E | 7 | Thymidine |
| 1 | E | 8 | Methylthioadenosine |
| 1 | E | 9 | 2-Phosphoglycerate |
| 1 | E | 10 | Tetrahydrofolate |
| 1 | E | 11 | Methionine |
| 1 | E | 12 | Glycine |
| 1 | F | 1 | Formamide |
| 1 | F | 2 | Guanidinoacetate |
| 1 | F | 3 | Malonate |
| 1 | F | 4 | Dihydroorotate |
| 1 | F | 5 | Quinate |
| 1 | F | 6 | Creatinine |
| 1 | F | 7 | Sarcosine |
| 1 | F | 8 | Glycolate |
| 1 | F | 9 | N-Acetylglucosamine |
| 1 | F | 10 | P-Hydroxyphenylacetate |
| 1 | F | 11 | Ascorbate |
| 1 | F | 12 | Glutamate |
| 1 | G | 1 | Pyrazole |
| 1 | G | 2 | Isoleucine |
| 1 | G | 3 | Cytosine |
| 1 | G | 4 | Gamma-Aminobutyrate |
| 1 | G | 5 | Inosine |
| 1 | G | 6 | Nicotinate |
| 1 | G | 7 | N-Acetyltryptophan |
| 1 | G | 8 | Taurine |
| 1 | G | 9 | Citrulline |
| 1 | G | 10 | Cysteine |
| 1 | G | 11 | Serine |
| 1 | G | 12 | Cytidine |
| 1 | H | 1 | Urate |
| 1 | H | 2 | Trans-Aconitate |
| 1 | H | 3 | Pyrimidine |
| 1 | H | 4 | N-Acetylmannosamine |
| 1 | H | 5 | N-Acetylneuraminate |
| 1 | H | 6 | Purine |
| 1 | H | 7 | Threonine |
| 1 | H | 8 | Citrate |
| 1 | H | 9 | N-Metyl-Alanine |
| 1 | H | 10 | Hypotaurine |
| 1 | H | 11 | Glutamine |
| 1 | H | 12 | Beta-Nicotinamide Adenine Dinucleotide |
| 2 | A | 1 | Diaminopimelate |
| 2 | A | 2 | Aminoadipate |
| 2 | A | 3 | Deoxycytidine |
| 2 | A | 4 | Noradrenaline |
| 2 | A | 5 | Glucosamine 6-Phosphate |
| 2 | A | 6 | Tartrate |
| 2 | A | 7 | 3-Dehydroshikimate |
| 2 | A | 8 | Caffeine |
| 2 | A | 9 | Homocysteine |
| 2 | A | 10 | Theophylline |
| 2 | A | 11 | Leucine |
| 2 | A | 12 | Trehalose |
| 2 | B | 1 | Betaine |
| 2 | B | 2 | Tryptophan |
| 2 | B | 3 | 3-Sulfinoalanine |
| 2 | B | 4 | O-Succinyl-Homoserine |
| 2 | B | 5 | Allantoin |
| 2 | B | 6 | Glyceraldehyde |
| 2 | B | 7 | D-Glucuronolactone |
| 2 | B | 8 | (2-Aminoethyl)Phosphonate |
| 2 | B | 9 | 2,5-Dihydrobenzoic Acid |
| 2 | B | 10 | Maleimide |
| 2 | B | 11 | Threitol |
| 2 | B | 12 | Glucosamine |
| 2 | C | 1 | Paraxanthine |
| 2 | C | 2 | Adenosine 5'-Diphosphate |
| 2 | C | 3 | 2-Deoxy-D-Glucose |
| 2 | C | 4 | 1-Methyl-L-Histidine |
| 2 | C | 5 | Galactitol |
| 2 | C | 6 | Oxoproline |
| 2 | C | 7 | 4-Pyridoxate |
| 2 | C | 8 | Quinolinate |

| PLATE | ROW | COL | PRIMARY NAME |
|---|---|---|---|
| 2 | C | 9 | Methylguanidine |
| 2 | C | 10 | Deoxyguanosine-Monophosphate |
| 2 | C | 11 | 3-Hydroxy-3-Methylglutaryl-Coa |
| 2 | C | 12 | Glucuronate |
| 2 | D | 1 | 1-Methyladenosine |
| 2 | D | 2 | Deoxyuridine |
| 2 | D | 3 | Gluconate |
| 2 | D | 4 | Urocanate |
| 2 | D | 5 | Kynurenine |
| 2 | D | 6 | Pyroglutamate |
| 2 | D | 7 | 4-Acetamidobutanoate |
| 2 | D | 8 | Trans-1,2-Cyclohexanediol |
| 2 | D | 9 | Melanin |
| 2 | D | 10 | Dopamine |
| 2 | D | 11 | Adenosine-Monophosphate |
| 2 | D | 12 | Lysine |
| 2 | E | 1 | Citicoline |
| 2 | E | 2 | 1,3-Diaminopropane |
| 2 | E | 3 | Phosphoserine |
| 2 | E | 4 | 1-Aminocyclopropanecarboxylate |
| 2 | E | 5 | Glutarylcarnitine |
| 2 | E | 6 | Cystathionine |
| 2 | E | 7 | Norvaline |
| 2 | E | 8 | 3-Hydroxymethylglutarate |
| 2 | E | 9 | Phosphonoacetate |
| 2 | E | 10 | Picolinate |
| 2 | E | 11 | Ethanolamine |
| 2 | E | 12 | Arginine |
| 2 | F | 1 | Trans-4-Hydroxy-L-Proline |
| 2 | F | 2 | Fucose |
| 2 | F | 3 | Homocystine |
| 2 | F | 4 | N-Methylglutamate |
| 2 | F | 5 | D-Ornithine |
| 2 | F | 6 | Xanthosine |
| 2 | F | 7 | 3-Methylcrotonyl-Coa |
| 2 | F | 8 | Thyrotropin Releasing Hormone |
| 2 | F | 9 | Cysteate |
| 2 | F | 10 | N-Methylaspartate |
| 2 | F | 11 | Galactarate |
| 2 | F | 12 | Alpha-Hydroxyisobutyrate |
| 2 | G | 1 | Nicotinic Acid Adenine Dinucleotide Phosphate |
| 2 | G | 2 | N-Acetylasparagine |
| 2 | G | 3 | Pipecolate |
| 2 | G | 4 | Glucose 6-Phosphate |
| 2 | G | 5 | Nadp |
| 2 | G | 6 | 6-Phosphogluconate |
| 2 | G | 7 | Isopentenyl Pyrophosphate |
| 2 | G | 8 | Guanosine Triphosphate |
| 2 | G | 9 | Dtdp-D-Glucose |
| 2 | G | 10 | Agmatine Sulfate |
| 2 | G | 11 | Glycolaldehyde |
| 2 | G | 12 | Dgtp |
| 2 | H | 1 | N-Acetylglycine |
| 2 | H | 2 | N-Acetylaspartate |
| 2 | H | 3 | Inosine 5'-Diphosphate |
| 2 | H | 4 | Palmitoylcarnitine |
| 2 | H | 5 | Norspermidine |
| 2 | H | 6 | Nicotinamide Hypoxanthine Dinucleotide |
| 2 | H | 7 | S-Adenosylmethionine |
| 2 | H | 8 | Erythritol |
| 2 | H | 9 | Glucosaminate |
| 2 | H | 10 | Uridine Triphosphate |

| PLATE | ROW | COL | PRIMARY NAME |
|---|---|---|---|
| 2 | H | 11 | 2-Keto-3-Deoxy-D-Gluconic Acid |
| 2 | H | 12 | D-Sedoheptulose |
| 3 | A | 1 | 1,4-Diaminobutane Dihydrocloride |
| 3 | A | 2 | Deoxycarnitine |
| 3 | A | 3 | Adenosine 2',3'-Cyclic Phosphate |
| 3 | A | 4 | Mevalolactone |
| 3 | A | 5 | Galactose 1-Phosphate |
| 3 | A | 6 | Gamma,Gamma-Dimethylallyl Pyrophosphate |
| 3 | A | 7 | Deoxyuridine Triphosphate |
| 3 | A | 8 | Phosphorylcholine |
| 3 | A | 9 | O-Acetylcarnitine |
| 3 | A | 10 | 6-Hydroxydopamine |
| 3 | A | 11 | Thiamine |
| 3 | A | 12 | Dgdp |
| 3 | B | 1 | 5-Methylcytosine |
| 3 | B | 2 | Glycerate |
| 3 | B | 3 | Cytidine 2',3'-Cyclic Phosphate |
| 3 | B | 4 | N,N,N-Trimethyllysine |
| 3 | B | 5 | Riboflavin |
| 3 | B | 6 | Uridine Diphosphate Glucose |
| 3 | B | 7 | Methyl Galactoside |
| 3 | B | 8 | Pyridoxal-Phosphate |
| 3 | B | 9 | Dihydroxyacetone Phosphate |
| 3 | B | 10 | Phosphoenolpyruvate |
| 3 | B | 11 | Mannose 6-Phosphate |
| 3 | B | 12 | 3-Phosphoglycerate |
| 3 | C | 1 | L-Carnitine |
| 3 | C | 2 | O-Phosphoethanolamine |
| 3 | C | 3 | O-Acetylserine |
| 3 | C | 4 | Cytidine Monophosphate |
| 3 | C | 5 | Guanosine Diphosphate Mannose |
| 3 | C | 6 | Adp-Glucose |
| 3 | C | 7 | Fructose 6-Phosphate |
| 3 | C | 8 | Adenosine 3',5'-Diphosphate |
| 3 | C | 9 | 3-Nitro-L-Tyrosine |
| 3 | C | 10 | P-Octopamine |
| 3 | C | 11 | N-Alpha-Acetyllysine |
| 3 | C | 12 | Uridine Diphosphategalactose |
| 3 | D | 1 | Dihydroxyfumarate |
| 3 | D | 2 | Pyridoxamine |
| 3 | D | 3 | 5-Aminolevulinate |
| 3 | D | 4 | Deoxyuridine-Monophosphate |
| 3 | D | 5 | 5'-Deoxyadenosine |
| 3 | D | 6 | Ribose 1,5-Bisphosphate |
| 3 | D | 7 | Xanthosine-Monophosphate |
| 3 | D | 8 | Fad |
| 3 | D | 9 | Deoxyguanosine |
| 3 | D | 10 | Orotate |
| 3 | D | 11 | Lauroylcarnitine |
| 3 | D | 12 | 1-Methylnicotinamide |
| 3 | E | 1 | Spermine |
| 3 | E | 2 | N-Acetylmethionine |
| 3 | E | 3 | Carbamoyl Phosphate |
| 3 | E | 4 | Phosphoribosyl Pyrophosphate |
| 3 | E | 5 | Aicar |
| 3 | E | 6 | Uridine Diphosphate-N-Acetylgalactosamine |
| 3 | E | 7 | Glyceraldehyde 3-Phosphate |
| 3 | E | 8 | Cyclic Gmp |
| 3 | E | 9 | Homocysteine Thiolactone |
| 3 | E | 10 | O-Phosphoserine |
| 3 | E | 11 | S-Adenosylhomocysteine |
| 3 | E | 12 | L-Ornithine |

| PLATE | ROW | COL | PRIMARY NAME |
|---|---|---|---|
| 3 | F | 1 | Adenine |
| 3 | F | 2 | Normetanephrine |
| 3 | F | 3 | Uridine Diphosphate-N-Acetylglucosamine |
| 3 | F | 4 | Guanosine Diphosphate |
| 3 | F | 5 | Glutathione Reduced |
| 3 | F | 6 | Uridine Diphosphate Glucuronic Acid |
| 3 | F | 7 | N,N-Dimethylarginine |
| 3 | F | 8 | Cytidine Diphosphate |
| 3 | F | 9 | Selenocystamine |
| 3 | F | 10 | Histamine |
| 3 | F | 11 | Indoxyl Sulfate |
| 3 | F | 12 | Ethyl 3-Ureidopropionate |
| 3 | G | 1 | Deoxyribose |
| 3 | G | 2 | Phytate |
| 3 | G | 3 | Thiamine Monophosphate |
| 3 | G | 4 | Uracil 5-Carboxylate |
| 3 | G | 5 | S-Hexyl-Glutathione |
| 3 | G | 6 | Glyoxylate |
| 3 | G | 7 | Guanosine Monophosphate |
| 3 | G | 8 | N-Acetylalanine |
| 3 | G | 9 | 4-Guanidinobutanoate |
| 3 | G | 10 | Hydroxypyruvate |
| 3 | G | 11 | D-Mannosamine |
| 3 | G | 12 | Cytochrome C |
| 4 | A | 1 | Deoxyadenosine |
| 4 | A | 2 | N-Acetylputrescine |
| 4 | A | 3 | N-Acetylgalactosamine |
| 4 | A | 4 | N-Acetylglutamate |
| 4 | A | 5 | 2,4-Dihydroxypteridine |
| 4 | A | 6 | 6-Hydroxynicotinate |
| 4 | A | 7 | N-Acetylcysteine |
| 4 | A | 8 | Inosine-Monophosphate |
| 4 | A | 9 | Pantothenate |
| 4 | A | 10 | 2-Aminoisobutyrate |
| 4 | A | 11 | Aniline-2-Sulfonate |
| 4 | A | 12 | S-Carboxymethylcysteine |
| 4 | B | 1 | Rhamnose |
| 4 | B | 2 | Thiamine Pyrophosphate |
| 4 | B | 3 | Histidinol |
| 4 | B | 4 | Thymidine-Monophosphate |
| 4 | B | 5 | Ureidopropionate |
| 4 | B | 6 | 5-Aminopentanoate |
| 4 | B | 7 | Norleucine |
| 4 | B | 8 | N-Formylglycine |
| 4 | B | 9 | Adenosine |
| 4 | B | 10 | Raffinose |
| 4 | B | 11 | Meso-Tartrate |
| 4 | B | 12 | 2-Acetamido-2-Deoxy-Beta-D-Glucosylamine |
| 4 | C | 1 | Saccharate |
| 4 | C | 2 | Adenosine Triphosphate |
| 4 | C | 3 | 3-Methoxytyrosine |
| 4 | C | 4 | Lactose |
| 4 | C | 5 | 3-Hydroxybutanoate |
| 4 | C | 6 | 4-Imidazoleacetate |
| 4 | C | 7 | Galacturonate |
| 4 | C | 8 | Cytidine Triphosphate |
| 4 | C | 9 | Cyclic Amp |
| 4 | C | 10 | Methionine Sulfoximine |
| 4 | C | 11 | Cis-4-Hydroxy-D-Proline |
| 4 | C | 12 | N1-Acetylspermine |
| 4 | D | 1 | Glucosamine 6-Sulfate |
| 4 | D | 2 | Nadph |
| 4 | D | 3 | 3-Methylhistamine |
| 4 | D | 4 | Maleamate |
| 4 | D | 5 | Choline |

| PLATE | ROW | COL | PRIMARY NAME |
|---|---|---|---|
| 4 | D | 6 | Methyl 4-Aminobutyrate |
| 4 | D | 7 | N-Formyl-L-Methionine |
| 4 | D | 8 | Acetylcholine |
| 4 | D | 9 | Oxalate |
| 4 | D | 10 | 5-Hydroxy-L-Tryptophan |
| 4 | D | 11 | D-Alanine |
| 4 | D | 12 | Theobromine |
| 4 | E | 1 | Guanidinosuccinate |
| 4 | E | 2 | Histidine |
| 4 | E | 3 | Allothreonine |
| 4 | E | 4 | Phosphocreatine |
| 4 | E | 5 | Spermidine |
| 4 | E | 6 | Adenosine Diphosphate Ribose |
| 4 | E | 7 | 2-Methoxyethanol |
| 4 | E | 8 | Citramalate |
| 4 | E | 9 | Anserine |
| 4 | E | 10 | Biliverdin |
| 4 | E | 11 | 5-Hydroxylysine |
| 4 | E | 12 | Cysteamine |
| 4 | F | 1 | Ophthalmate |
| 4 | F | 2 | Mesoxalate |
| 4 | F | 3 | Trigonelline |
| 4 | F | 4 | Epinephrine |
| 4 | F | 5 | 3,4-Dihydroxyphenylglycol |
| 4 | F | 6 | Cadaverine |
| 4 | F | 7 | 2-Hydroxybutyrate |
| 4 | F | 8 | Coenzyme A |
| 4 | F | 9 | Oxalomalate |
| 4 | F | 10 | Inosine Triphosphate |
| 4 | F | 11 | Cdp-Ethanolamine |
| 4 | F | 12 | 2,5-Dimethylpyrazine |
| 4 | G | 1 | Stachyose |
| 4 | G | 2 | Deoxycytidine-Diphosphate |
| 4 | G | 3 | 2,3-Butanediol |
| 4 | G | 4 | D-Ribose 5-Phosphate |
| 4 | G | 5 | Hydroxykynurenine |
| 4 | G | 6 | Galactosamine |
| 4 | G | 7 | Deoxyadenosine Triphosphate |
| 4 | G | 8 | Glycerol 3-Phosphate |
| 4 | G | 9 | Cyanocobalamin |
| 4 | G | 10 | 4-Hydroxy-L-Phenylglycine |
| 4 | G | 11 | N-Acetylserine |
| 4 | G | 12 | Uridine 5'-Diphosphate |
| 5 | A | 1 | Methylglutarate |
| 5 | A | 2 | Sorbate |
| 5 | A | 3 | Monoethylmalonate |
| 5 | A | 4 | Gluconolactone |
| 5 | A | 5 | 4-Hydroxybenzoate |
| 5 | A | 6 | Tyramine |
| 5 | A | 7 | Cortisol |
| 5 | A | 8 | Prenol |
| 5 | A | 9 | 3-Hydroxybenzaldehyde |
| 5 | A | 10 | Xanthurenate |
| 5 | A | 11 | 2-Methylpropanal |
| 5 | A | 12 | Indoxyl B-Glucoside |
| 5 | B | 1 | Trimethylamine |
| 5 | B | 2 | Melatonin |
| 5 | B | 3 | Maleate |
| 5 | B | 4 | Pentanoate |
| 5 | B | 5 | Propanoate |
| 5 | B | 6 | Bilirubin |
| 5 | B | 7 | Nicotine |
| 5 | B | 8 | Pregnenolone Sulfate |
| 5 | B | 9 | Kynurenate |
| 5 | B | 10 | Isobutyrate |
| 5 | B | 11 | 3-Hydroxybenzyl Alcohol |
| 5 | B | 12 | Aniline |
| 5 | C | 1 | Acetoin |

| PLATE | ROW | COL | PRIMARY NAME |
|---|---|---|---|
| 5 | C | 2 | 3,5-Diiodo-L-Tyrosine |
| 5 | C | 3 | Mandelate |
| 5 | C | 4 | Tryptamine |
| 5 | C | 5 | 4-Aminobenzoate |
| 5 | C | 6 | Glutarate |
| 5 | C | 7 | 5-Valerolactone |
| 5 | C | 8 | Caffeate |
| 5 | C | 9 | Lumichrome |
| 5 | C | 10 | Beta-Alanine |
| 5 | C | 11 | N-Acetylphenylalanine |
| 5 | C | 12 | N-Acetylproline |
| 5 | D | 1 | L-Tryptophanamide |
| 5 | D | 2 | Phenol |
| 5 | D | 3 | N-Methyltryptamine |
| 5 | D | 4 | Oxaloacetate |
| 5 | D | 5 | 2,3-Dihydroxybenzoate |
| 5 | D | 6 | 2-Propenoate |
| 5 | D | 7 | Indole-3-Ethanol |
| 5 | D | 8 | Ferulate |
| 5 | D | 9 | Glycocholate |
| 5 | D | 10 | Phenylethanolamine |
| 5 | D | 11 | Thiopurine S-Methylether |
| 5 | D | 12 | 2-Hydroxy-4-(Methylthio)Butanoate |
| 5 | E | 1 | Glycochenodeoxycholate |
| 5 | E | 2 | Benzoate |
| 5 | E | 3 | 3-Amino-5-Hydroxybenzoate |
| 5 | E | 4 | Pyrocatechol |
| 5 | E | 5 | 3,4-Dihydroxybenzoate |
| 5 | E | 6 | Cyclopentanone |
| 5 | E | 7 | Pantolactone |
| 5 | E | 8 | Guaiacol |
| 5 | E | 9 | 2-Hydroxyphenylacetate |
| 5 | E | 10 | 10-Hydroxydecanoate |
| 5 | E | 11 | Didecanoyl-Glycerophosphocholine |
| 5 | E | 12 | 2-Hydroxypyridine |
| 5 | F | 1 | 3,4-Dihydroxyphenylacetate |
| 5 | F | 2 | N6-(Delta2-Isopentenyl)-Adenine |
| 5 | F | 3 | Methyl Vanillate |
| 5 | F | 4 | 2-Oxobutanoate |
| 5 | F | 5 | Lipoamide |
| 5 | F | 6 | 3-Hydroxyanthranilate |
| 5 | F | 7 | 3-(4-Hydroxyphenyl)Pyruvate |
| 5 | F | 8 | Hexanoate |
| 5 | F | 9 | Methylmalonate |
| 5 | F | 10 | Indole-3-Acetate |
| 5 | F | 11 | Cortisol 21-Acetate |
| 5 | F | 12 | Indole-3-Acetamide |
| 5 | G | 1 | Hippurate |
| 5 | G | 2 | Ethylmalonate |
| 5 | G | 3 | 3,5-Diiodo-L-Thyronine |
| 5 | G | 4 | Fumarate |
| 5 | G | 5 | Benzaldehyde |
| 5 | G | 6 | 4-Hydroxybenzaldehyde |
| 5 | G | 7 | 3-(2-Hydroxyphenyl)Propanoate |
| 5 | G | 8 | 3-Methoxytyramine |
| 5 | G | 9 | Benzylamine |
| 5 | G | 10 | 2-Quinolinecarboxylate |
| 5 | G | 11 | Serotonin |
| 5 | G | 12 | Pterin |
| 5 | H | 1 | Butanoate |
| 5 | H | 2 | 2-Aminophenol |
| 5 | H | 3 | 6-Carboxyhexanoate |
| 5 | H | 4 | Indole-3-Pyruvate |
| 5 | H | 5 | Dehydroascorbate |
| 5 | H | 6 | 3-Amino-4-Hydroxybenzoate |

| PLATE | ROW | COL | PRIMARY NAME |
|---|---|---|---|
| 5 | H | 7 | 3,4 Dihydroxymandelate |
| 5 | H | 8 | 2-Methylcitrate |
| 5 | H | 9 | Dihydrobiopterin |
| 5 | H | 10 | Beta-Glycerophosphate |
| 5 | H | 11 | Glucose 1-Phosphate |
| 5 | H | 12 | 2,3-Diaminopropionate |
| 6 | A | 1 | 2,5-Dihydroxybenzoate |
| 6 | A | 2 | 4-Quinolinecarboxylate |
| 6 | A | 3 | Hydroquinone |
| 6 | A | 4 | Dethiobiotin |
| 6 | A | 5 | 3-Hydroxybenzoate |
| 6 | A | 6 | 2-Methylbutanal |
| 6 | A | 7 | N-Acetylserotonin |
| 6 | A | 8 | Hydrophenyllactic Acid |
| 6 | A | 9 | Itaconate |
| 6 | A | 10 | Azelate |
| 6 | A | 11 | Oxoadipate |
| 6 | A | 12 | 2-Methylglutarate |
| 6 | B | 1 | Phenylacetaldehyde |
| 6 | B | 2 | 3-Methyl-2-Oxovalerate |
| 6 | B | 3 | Porphobilinogen |
| 6 | B | 4 | Diacetyl |
| 6 | B | 5 | Pyruvate |
| 6 | B | 6 | Trans-Cinnamaldehyde |
| 6 | B | 7 | 2,6-Dihydroxypyridine |
| 6 | B | 8 | Vanillin |
| 6 | B | 9 | Methyl Acetoacetate |
| 6 | B | 10 | Suberate |
| 6 | B | 11 | Adipate |
| 6 | B | 12 | Geranyl-Pp |
| 6 | C | 1 | N-Acetylleucine |
| 6 | C | 2 | 2',4'-Dihydroxyacetophenone |
| 6 | C | 3 | Benzyl Alcohol |
| 6 | C | 4 | Monomethylglutarate |
| 6 | C | 5 | Indole-3-Methyl Acetate |
| 6 | C | 6 | Mevalonate |
| 6 | C | 7 | 3-Methoxy-4-Hydroxymandelate |
| 6 | C | 8 | Homovanillate |
| 6 | C | 9 | 2-Methylmaleate |
| 6 | C | 10 | 1-Phenylethanol |
| 6 | C | 11 | Salsolinol |
| 6 | C | 12 | Salicylamide |
| 6 | D | 1 | Oxoglutarate |
| 6 | D | 2 | Ethyl 3-Indoleacetate |
| 6 | D | 3 | 3-Alpha,11-Beta,17,21-Tetrahydroxy- 5-Beta-Pregnan-20-One |
| 6 | D | 4 | N,N-Dimethyl-1,4-Phenylenediamine |
| 6 | D | 5 | Homogentisate |
| 6 | D | 6 | Indoleacetaldehyde |
| 6 | D | 7 | 4-Hydroxy-3-Methoxyphenylglycol |
| 6 | D | 8 | 3-Hydroxyphenylacetate |
| 6 | D | 9 | 4-Methylcatechol |
| 6 | D | 10 | Pyridoxal |
| 6 | D | 11 | Salicylate |
| 6 | D | 12 | Sebacate |
| 6 | E | 1 | 3-Methyl-2-Oxindole |
| 6 | E | 2 | 3-Methyladenine |
| 6 | E | 3 | Hydroxyphenyllactate |
| 6 | E | 4 | Biotin |
| 6 | E | 5 | Mercaptopyruvate |
| 6 | E | 6 | Pyruvic Aldehyde |
| 6 | E | 7 | Pyrrole-2-Carboxylate |
| 6 | E | 8 | 5-Hydroxyindoleacetate |
| 6 | E | 9 | 3-Methylglutaconate |
| 6 | E | 10 | Resorcinol Monoacetate |

| PLATE | ROW | COL | PRIMARY NAME |
|---|---|---|---|
| 6 | E | 11 | Acetoacetate |
| 6 | E | 12 | Acetylphosphate |
| 6 | F | 1 | Sorbose |
| 6 | F | 2 | Xylitol |
| 6 | F | 3 | Ribitol |
| 6 | F | 4 | Myoinositol |
| 6 | F | 5 | Mannose |
| 6 | F | 6 | Xylose |
| 6 | F | 7 | Sucrose |
| 6 | F | 8 | Galactose |
| 6 | F | 9 | Alpha-D-Glucose |
| 6 | F | 10 | Allose |
| 6 | F | 11 | Mannitol |
| 6 | F | 12 | Melibiose |
| 6 | G | 1 | Sorbitol |
| 6 | G | 2 | Maltose |
| 6 | G | 3 | Tagatose |
| 6 | G | 4 | L-Gulonolactone |
| 6 | G | 5 | Arabinose |
| 6 | G | 6 | Cellobiose |
| 6 | G | 7 | Psicose |
| 6 | G | 8 | Arabitol |
| 6 | G | 9 | Lyxose |
| 6 | G | 10 | Ribose |
| 6 | G | 11 | Palatinose |
| 6 | G | 12 | D-Pinitol |
| 7 | A | 1 | Vitamin D2 |
| 7 | A | 2 | Squalene |
| 7 | A | 3 | 4-Coumarate |
| 7 | A | 4 | Nonanoate |
| 7 | A | 5 | Estradiol-17alpha |
| 7 | A | 6 | Caprylate |
| 7 | A | 7 | Ursodeoxycholate |
| 7 | A | 8 | Petroselinate |
| 7 | A | 9 | Dipalmitoylglycerol |
| 7 | A | 10 | Corticosterone |
| 7 | A | 11 | Lithocholate |
| 7 | A | 12 | Protoporphyrin |
| 7 | B | 1 | Heptanoate |
| 7 | B | 2 | Retinol |
| 7 | B | 3 | Menaquinone |
| 7 | B | 4 | Elaidate |
| 7 | B | 5 | Chenodeoxycholate |
| 7 | B | 6 | Myristate |
| 7 | B | 7 | Cholesteryl Oleate |
| 7 | B | 8 | Rosmarinate |
| 7 | B | 9 | Glyceryl Tripalmitate |
| 7 | B | 10 | Cortexolone |
| 7 | B | 11 | Lithocholyltaurine |
| 7 | B | 12 | Palmitoleate |
| 7 | C | 1 | Palmitate |
| 7 | C | 2 | Liothyronine |
| 7 | C | 3 | Sphinganine |
| 7 | C | 4 | Lanosterol |
| 7 | C | 5 | Laurate |
| 7 | C | 6 | Arachidate |
| 7 | C | 7 | Erucate |
| 7 | C | 8 | Deoxycholate |
| 7 | C | 9 | Ketoleucine |
| 7 | C | 10 | Eicosapentaenoate |
| 7 | C | 11 | Heptadecanoate |
| 7 | C | 12 | Glyceryl Trimyristate |
| 7 | D | 1 | Linoleate |
| 7 | D | 2 | Sphingomyelin |
| 7 | D | 3 | 7-Dehydrocholesterol |
| 7 | D | 4 | Thyroxine |
| 7 | D | 5 | Bis(2-Ethylhexyl)Phthalate |
| 7 | D | 6 | Gamma-Linolenate |

| PLATE | ROW | COL | PRIMARY NAME |
|---|---|---|---|
| 7 | D | 7 | Omega-Hydroxydodecanoate |
| 7 | D | 8 | Methyl Jasmonate |
| 7 | D | 9 | Dipalmitoyl-Phosphatidylcholine |
| 7 | D | 10 | Hexadecanol |
| 7 | D | 11 | 5,6 Dimethylbenzimidazole |
| 7 | D | 12 | Retinoate |
| 7 | E | 1 | Indole |
| 7 | E | 2 | Cholate |
| 7 | E | 3 | Phylloquinone |
| 7 | E | 4 | Cholesteryl Palmitate |
| 7 | E | 5 | Quinoline |
| 7 | E | 6 | Docosahexaenoate |
| 7 | E | 7 | Diethyl 2-Methyl-3-Oxosuccinate |
| 7 | E | 8 | Retinyl Palmitate |
| 7 | E | 9 | 2-Undecanone |
| 7 | E | 10 | 1-Hydroxy-2-Naphthoate |
| 7 | E | 11 | Dipalmitoyl-Phosphoethanolamine |
| 7 | E | 12 | Phenylpyruvate |
| 7 | F | 1 | Trans-Cinnamate |
| 7 | F | 2 | Oleate |
| 7 | F | 3 | Stearate |
| 7 | F | 4 | Beta-Carotene |
| 7 | F | 5 | 25-Hydroxycholesterol |
| 7 | F | 6 | Nervonate |
| 7 | F | 7 | Desmosterol |
| 7 | F | 8 | Deoxycorticosterone Acetate |
| 7 | F | 9 | Oleoyl-Glycerol |
| 7 | F | 10 | Alpha-Tocopherol |
| 7 | F | 11 | Glycerol-Myristate |
| 7 | F | 12 | Tricosanoate |
| 7 | G | 1 | Coenzyme Q10 |
| 7 | G | 2 | Cortisone |
| 7 | G | 3 | Decanoate |

**Table S2**. Partition of all metabolites in each one of the 29 vials. Metabolites were grouped in vials according to their Monoisotopic Molecular Mass (MMI) so that there were no compounds with the same molecular weight in the same pool.

| VIAL | MMI | PRIMARY NAME |
|---|---|---|
| 1 | 92.04734 | Glycerol |
| 1 | 181.07389 | Tyrosine |
| 1 | 192.02700 | Isocitrate |
| 1 | 334.05660 | Nicotinamide Mononucleotide |
| 1 | 441.13968 | Folate |
| 1 | 331.06817 | Deoxyadenosine Monophosphate |
| 1 | 169.07389 | Pyridoxine |
| 1 | 119.05824 | Homoserine |
| 1 | 151.04941 | Guanine |
| 1 | 117.07898 | Valine |
| 1 | 132.05349 | Asparagine |
| 1 | 134.02152 | Malate |
| 1 | 114.04293 | Dihydrouracil |
| 1 | 283.09167 | Guanosine |
| 1 | 197.06881 | L-Dopa |
| 1 | 131.06948 | Creatine |
| 1 | 136.03851 | Hypoxanthine |
| 1 | 307.05694 | Deoxycytidine Monophosphate |
| 1 | 133.03751 | Aspartate |
| 1 | 76.00952 | Thiourea |
| 1 | 152.06847 | Arabitol |
| 2 | 169.08513 | 3-Methyl-L-Histidine |
| 2 | 112.02728 | Uracil |
| 2 | 165.07898 | Phenylalanine |
| 2 | 118.02661 | Succinate |
| 2 | 174.05282 | Shikimate |
| 2 | 324.03587 | Uridine Monophosphate |
| 2 | 105.07898 | Diethanolamine |
| 2 | 89.04768 | L-Alanine |
| 2 | 122.04801 | Nicotinamide |
| 2 | 226.10659 | Carnosine |
| 2 | 339.99605 | Fructose Bisphosphate |
| 2 | 244.06954 | Uridine |
| 2 | 90.03169 | Lactate |
| 2 | 102.03169 | Succinate Semialdehyde |
| 2 | 126.04293 | Thymine |
| 2 | 115.06333 | Proline |
| 2 | 240.02385 | Cystine |
| 2 | 443.15533 | Dihydrofolate |
| 2 | 152.03343 | Xanthine |
| 2 | 180.06339 | Gulose |
| 2 | 150.05282 | Ribose |
| 2 | 342.11622 | Palatinose |
| 3 | 445.17098 | Tetrahydrofolate |
| 3 | 149.05105 | Methionine |
| 3 | 75.03203 | Glycine |
| 3 | 103.06333 | Aminoisobutanoate |
| 3 | 178.04121 | Cys-Gly |
| 3 | 242.09027 | Thymidine |
| 3 | 297.08956 | Methylthioadenosine |
| 3 | 185.99294 | 2-Phosphoglycerate |
| 3 | 45.02146 | Formamide |
| 3 | 152.04734 | P-Hydroxyphenylacetate |
| 3 | 176.03209 | Ascorbate |
| 3 | 147.05316 | Glutamate |
| 3 | 117.05383 | Guanidinoacetate |
| 3 | 104.01096 | Malonate |

| VIAL | MMI | PRIMARY NAME |
|---|---|---|
| 3 | 158.03276 | Dihydroorotate |
| 3 | 192.06339 | Quinate |
| 3 | 113.05891 | Creatinine |
| 3 | 89.04768 | Sarcosine |
| 3 | 76.01604 | Glycolate |
| 3 | 221.08994 | N-Acetylglucosamine |
| 3 | 180.06339 | Sorbose |
| 3 | 194.07904 | D-Pinitol |
| 4 | 68.03745 | Pyrazole |
| 4 | 121.01975 | Cysteine |
| 4 | 105.04259 | Serine |
| 4 | 243.08552 | Cytidine |
| 4 | 131.09463 | Isoleucine |
| 4 | 111.04326 | Cytosine |
| 4 | 103.06333 | Gamma-Aminobutyrate |
| 4 | 268.08077 | Inosine |
| 4 | 123.03203 | Nicotinate |
| 4 | 246.10044 | N-Acetyltryptophan |
| 4 | 125.01466 | Taurine |
| 4 | 175.09569 | Citrulline |
| 4 | 168.02834 | Urate |
| 4 | 174.01644 | Trans-Aconitate |
| 4 | 80.03745 | Pyrimidine |
| 4 | 221.08994 | N-Acetylmannosamine |
| 4 | 309.10598 | N-Acetylneuraminate |
| 4 | 120.04360 | Purine |
| 4 | 119.05824 | Threonine |
| 4 | 192.02700 | Citrate |
| 4 | 180.06339 | Psicose |
| 5 | 109.01975 | Hypotaurine |
| 5 | 146.0691422 | Glutamine |
| 5 | 663.10912 | Beta-Nicotinamide Adenine Dinucleotide |
| 5 | 103.06333 | N-Metyl-Alanine |
| 5 | 190.09536 | Diaminopimelate |
| 5 | 180.06473 | Theophylline |
| 5 | 131.09463 | Leucine |
| 5 | 342.11621 | Trehalose |
| 5 | 161.06881 | Aminoadipate |
| 5 | 227.09061 | Deoxycytidine |
| 5 | 169.07389 | Noradrenaline |
| 5 | 259.04570 | Glucosamine 6-Phosphate |
| 5 | 150.01644 | Tartrate |
| 5 | 172.03717 | 3-Dehydroshikimate |
| 5 | 194.08038 | Caffeine |
| 5 | 135.03540 | Homocysteine |
| 5 | 117.07898 | Betaine |
| 5 | 204.08988 | Tryptophan |
| 5 | 153.00958 | 3-Sulfinoalanine |
| 5 | 219.07429 | O-Succinyl-Homoserine |
| 6 | 97.01638 | Maleimide |
| 6 | 122.05791 | Threitol |
| 6 | 179.07937 | Glucosamine |
| 6 | 158.04399 | Allantoin |
| 6 | 90.03169 | Glyceraldehyde |
| 6 | 176.03209 | D-Glucuronolactone |
| 6 | 125.02418 | (2-Aminoethyl)Phosphonate |
| 6 | 154.02661 | 2,5-Dihydrobenzoic Acid |

| VIAL | MMI | PRIMARY NAME |
|---|---|---|
| 6 | 180.06473 | Paraxanthine |
| 6 | 347.06308 | Deoxyguanosine-Monophosphate |
| 6 | 194.04265 | Glucuronate |
| 6 | 427.02941 | Adenosine 5'-Diphosphate |
| 6 | 164.06847 | 2-Deoxy-D-Glucose |
| 6 | 169.08513 | 1-Methyl-L-Histidine |
| 6 | 182.07904 | Galactitol |
| 6 | 129.04259 | Oxoproline |
| 6 | 183.05316 | 4-Pyridoxate |
| 6 | 167.02186 | Quinolinate |
| 6 | 73.06400 | Methylguanidine |
| 6 | 152.06847 | Xylitol |
| 6 | 150.05282 | Lyxose |
| 7 | 281.11240 | 1-Methyladenosine |
| 7 | 153.07898 | Dopamine |
| 7 | 347.06308 | Adenosine-Monophosphate |
| 7 | 146.10553 | Lysine |
| 7 | 228.07462 | Deoxyuridine |
| 7 | 196.05830 | Gluconate |
| 7 | 138.04293 | Urocanate |
| 7 | 208.08479 | Kynurenine |
| 7 | 129.04259 | Pyroglutamate |
| 7 | 145.07389 | 4-Acetamidobutanoate |
| 7 | 116.08373 | Trans-1,2-Cyclohexanediol |
| 7 | 318.06406 | Melanin |
| 7 | 488.10733 | Citicoline |
| 7 | 74.08440 | 1,3-Diaminopropane |
| 7 | 185.00892 | Phosphoserine |
| 7 | 101.04768 | 1-Aminocyclopropanecarboxylate |
| 7 | 275.13689 | Glutarylcarnitine |
| 7 | 222.06743 | Cystathionine |
| 7 | 117.07898 | Norvaline |
| 7 | 162.05282 | 3-Hydroxymethylglutarate |
| 7 | 152.06847 | Ribitol |
| 8 | 123.03203 | Picolinate |
| 8 | 61.05276 | Ethanolamine |
| 8 | 139.98746 | Phosphonoacetate |
| 8 | 131.05824 | Trans-4-Hydroxy-L-Proline |
| 8 | 147.05316 | N-Methylaspartate |
| 8 | 210.03757 | Galactarate |
| 8 | 104.04734 | Alpha-Hydroxyisobutyrate |
| 8 | 164.06847 | Fucose |
| 8 | 268.05515 | Homocystine |
| 8 | 161.06881 | N-Methylglutamate |
| 8 | 132.08988 | D-Ornithine |
| 8 | 284.07568 | Xanthosine |
| 8 | 362.17025 | Thyrotropin Releasing Hormone |
| 8 | 169.00449 | Cysteate |
| 8 | 745.06674 | Nicotinic Acid Adenine Dinucleotide Phosphate |
| 8 | 174.06406 | N-Acetylasparagine |
| 8 | 129.07898 | Pipecolate |
| 8 | 260.02972 | Glucose 6-Phosphate |
| 8 | 743.07545 | Nadp |
| 8 | 180.06339 | Myoinositol |
| 9 | 174.11168 | Arginine |
| 9 | 130.12185 | Agmatine Sulfate |
| 9 | 120.04226 | Glycolaldehyde |
| 9 | 506.99575 | Dgtp |
| 9 | 276.02463 | 6-Phosphogluconate |
| 9 | 246.00583 | Isopentenyl Pyrophosphate |
| 9 | 522.99066 | Guanosine Triphosphate |
| 9 | 564.07576 | Dtdp-D-Glucose |
| 9 | 117.04259 | N-Acetylglycine |
| 9 | 483.96853 | Uridine Triphosphate |

| VIAL | MMI | PRIMARY NAME |
|---|---|---|
| 9 | 178.04775 | 2-Keto-3-Deoxy-D-Gluconic Acid |
| 9 | 210.07395 | D-Sedoheptulose |
| 9 | 175.04807 | N-Acetylaspartate |
| 9 | 428.01343 | Inosine 5'-Diphosphate |
| 9 | 399.33486 | Palmitoylcarnitine |
| 9 | 131.14225 | Norspermidine |
| 9 | 664.09314 | Nicotinamide Hypoxanthine Dinucleotide |
| 9 | 122.05791 | Erythritol |
| 9 | 195.07429 | Glucosaminate |
| 9 | 160.05300 | 1,4-Diaminobutane Dihydrocloride |
| 9 | 180.06339 | Mannose |
| 10 | 399.14506 | S-Adenosylmethionine |
| 10 | 169.07389 | 6-Hydroxydopamine |
| 10 | 265.11231 | Thiamine |
| 10 | 427.02941 | Dgdp |
| 10 | 145.11028 | Deoxycarnitine |
| 10 | 329.05252 | Adenosine 2',3'-Cyclic Phosphate |
| 10 | 130.06299 | Mevalolactone |
| 10 | 260.02972 | Galactose 1-Phosphate |
| 10 | 246.00583 | Gamma,Gamma-Dimethylallyl Pyrophosphate |
| 10 | 467.97361 | Deoxyuridine Triphosphate |
| 10 | 184.07387 | Phosphorylcholine |
| 10 | 203.11576 | O-Acetylcarnitine |
| 10 | 125.05891 | 5-Methylcytosine |
| 10 | 106.02661 | Glycerate |
| 10 | 305.04129 | Cytidine 2',3'-Cyclic Phosphate |
| 10 | 188.15248 | N,N,N-Trimethyllysine |
| 10 | 376.13828 | Riboflavin |
| 10 | 566.05503 | Uridine Diphosphate Glucose |
| 10 | 194.07904 | Methyl Galactoside |
| 10 | 247.02457 | Pyridoxal-Phosphate |
| 10 | 150.05282 | Xylose |
| 11 | 167.98237 | Phosphoenolpyruvate |
| 11 | 260.02972 | Mannose 6-Phosphate |
| 11 | 185.99294 | 3-Phosphoglycerate |
| 11 | 169.99802 | Dihydroxyacetone Phosphate |
| 11 | 161.10519 | L-Carnitine |
| 11 | 153.07898 | P-Octopamine |
| 11 | 188.11609 | N-Alpha-Acetyllysine |
| 11 | 566.05502 | Uridine Diphosphategalactose |
| 11 | 141.01909 | O-Phosphoethanolamine |
| 11 | 147.05316 | O-Acetylserine |
| 11 | 323.05185 | Cytidine Monophosphate |
| 11 | 605.07715 | Guanosine Diphosphate Mannose |
| 11 | 589.08224 | Adp-Glucose |
| 11 | 427.02941 | Adenosine 3',5'-Diphosphate |
| 11 | 226.05897 | 3-Nitro-L-Tyrosine |
| 11 | 148.00079 | Dihydroxyfumarate |
| 11 | 168.08988 | Pyridoxamine |
| 11 | 131.05824 | 5-Aminolevulinate |
| 11 | 308.04095 | Deoxyuridine-Monophosphate |
| 11 | 251.10184 | 5'-Deoxyadenosine |
| 11 | 342.11621 | Sucrose |
| 12 | 260.02972 | Fructose 6-Phosphate |
| 12 | 156.01711 | Orotate |
| 12 | 343.27226 | Lauroylcarnitine |
| 12 | 137.07149 | 1-Methylnicotinamide |
| 12 | 309.98548 | Ribose 1,5-Bisphosphate |
| 12 | 364.04202 | Xanthosine-Monophosphate |
| 12 | 785.15713 | Fad |
| 12 | 267.09675 | Deoxyguanosine |
| 12 | 202.21575 | Spermine |

| VIAL | MMI | PRIMARY NAME |
|---|---|---|
| 12 | 185.00892 | O-Phosphoserine |
| 12 | 384.12159 | S-Adenosylhomocysteine |
| 12 | 132.08988 | L-Ornithine |
| 12 | 191.06161 | N-Acetylmethionine |
| 12 | 140.98271 | Carbamoyl Phosphate |
| 12 | 389.95181 | Phosphoribosyl Pyrophosphate |
| 12 | 338.06275 | Aicar |
| 12 | 607.08157 | Uridine Diphosphate-N-Acetylgalactosamine |
| 12 | 169.99803 | Glyceraldehyde 3-Phosphate |
| 12 | 345.04743 | Cyclic Gmp |
| 12 | 117.02483 | Homocysteine Thiolactone |
| 12 | 180.06339 | Galactose |
| 13 | 135.05450 | Adenine |
| 13 | 111.07965 | Histamine |
| 13 | 213.00958 | Indoxyl Sulfate |
| 13 | 160.08479 | Ethyl 3-Ureidopropionate |
| 13 | 183.08954 | Normetanephrine |
| 13 | 607.08157 | Uridine Diphosphate-N-Acetylglucosamine |
| 13 | 443.02433 | Guanosine Diphosphate |
| 13 | 307.08381 | Glutathione Reduced |
| 13 | 580.03428 | Uridine Diphosphate Glucuronic Acid |
| 13 | 202.14298 | N,N-Dimethylarginine |
| 13 | 403.01818 | Cytidine Diphosphate |
| 13 | 247.93309 | Selenocystamine |
| 13 | 134.05791 | Deoxyribose |
| 13 | 659.86137 | Phytate |
| 13 | 344.07081 | Thiamine Monophosphate |
| 13 | 156.01711 | Uracil 5-Carboxylate |
| 13 | 391.17771 | S-Hexyl-Glutathione |
| 13 | 74.00039 | Glyoxylate |
| 13 | 363.05800 | Guanosine Monophosphate |
| 13 | 131.05824 | N-Acetylalanine |
| 13 | 180.06339 | Alpha-D-Glucose |
| 14 | 104.01096 | Hydroxypyruvate |
| 14 | 179.07937 | D-Mannosamine |
| 14 | 145.08513 | 4-Guanidinobutanoate |
| 14 | 251.10184 | Deoxyadenosine |
| 14 | 103.06333 | 2-Aminoisobutyrate |
| 14 | 173.01467 | Aniline-2-Sulfonate |
| 14 | 130.11061 | N-Acetylputrescine |
| 14 | 221.08994 | N-Acetylgalactosamine |
| 14 | 189.06372 | N-Acetylglutamate |
| 14 | 164.03343 | 2,4-Dihydroxypteridine |
| 14 | 139.02694 | 6-Hydroxynicotinate |
| 14 | 163.03031 | N-Acetylcysteine |
| 14 | 348.04710 | Inosine-Monophosphate |
| 14 | 219.11067 | Pantothenate |
| 14 | 425.04497 | Thiamine Pyrophosphate |
| 14 | 141.09021 | Histidinol |
| 14 | 322.05660 | Thymidine-Monophosphate |
| 14 | 132.05349 | Ureidopropionate |
| 14 | 117.07898 | 5-Aminopentanoate |
| 14 | 180.06339 | Allose |
| 15 | 179.02523 | S-Carboxymethylcysteine |
| 15 | 164.06847 | Rhamnose |
| 15 | 504.16903 | Raffinose |
| 15 | 150.01644 | Meso-Tartrate |
| 15 | 220.10592 | 2-Acetamido-2-Deoxy-Beta-D-Glucosylamine |
| 15 | 131.09463 | Norleucine |
| 15 | 103.02694 | N-Formylglycine |
| 15 | 267.09675 | Adenosine |
| 15 | 210.03757 | Saccharate |
| 15 | 180.05686 | Methionine Sulfoximine |
| 15 | 244.22631 | N1-Acetylspermine |

| VIAL | MMI | PRIMARY NAME |
|---|---|---|
| 15 | 506.99575 | Adenosine Triphosphate |
| 15 | 211.08446 | 3-Methoxytyrosine |
| 15 | 342.11621 | Lactose |
| 15 | 104.04734 | 3-Hydroxybutanoate |
| 15 | 126.04293 | 4-Imidazoleacetate |
| 15 | 194.04265 | Galacturonate |
| 15 | 482.98451 | Cytidine Triphosphate |
| 15 | 329.05252 | Cyclic Amp |
| 15 | 259.03619 | Glucosamine 6-Sulfate |
| 15 | 182.07904 | Mannitol |
| 16 | 131.05824 | Cis-4-Hydroxy-D-Proline |
| 16 | 220.08479 | 5-Hydroxy-L-Tryptophan |
| 16 | 180.06473 | Theobromine |
| 16 | 745.09110 | Nadph |
| 16 | 125.09530 | 3-Methylhistamine |
| 16 | 115.02694 | Maleamate |
| 16 | 104.10754 | Choline |
| 16 | 117.07898 | Methyl 4-Aminobutyrate |
| 16 | 177.04596 | N-Formyl-L-Methionine |
| 16 | 146.11810 | Acetylcholine |
| 16 | 89.99531 | Oxalate |
| 16 | 175.05931 | Guanidinosuccinate |
| 16 | 155.06948 | Histidine |
| 16 | 119.05824 | Allothreonine |
| 16 | 211.03581 | Phosphocreatine |
| 16 | 145.15790 | Spermidine |
| 16 | 559.07167 | Adenosine Diphosphate Ribose |
| 16 | 76.05243 | 2-Methoxyethanol |
| 16 | 148.03717 | Citramalate |
| 16 | 240.12224 | Anserine |
| 16 | 342.11621 | Melibiose |
| 17 | 89.04768 | D-Alanine |
| 17 | 582.24783 | Biliverdin |
| 17 | 162.10044 | 5-Hydroxylysine |
| 17 | 77.02992 | Cysteamine |
| 17 | 289.12739 | Ophthalmate |
| 17 | 507.97976 | Inosine Triphosphate |
| 17 | 446.06038 | Cdp-Ethanolamine |
| 17 | 108.06875 | 2,5-Dimethylpyrazine |
| 17 | 117.99022 | Mesoxalate |
| 17 | 137.04768 | Trigonelline |
| 17 | 183.08954 | Epinephrine |
| 17 | 170.05791 | 3,4-Dihydroxyphenylglycol |
| 17 | 102.11570 | Cadaverine |
| 17 | 104.04734 | 2-Hydroxybutyrate |
| 17 | 767.11521 | Coenzyme A |
| 17 | 206.00627 | Oxalomalate |
| 17 | 666.22186 | Stachyose |
| 17 | 387.02327 | Deoxycytidine-Diphosphate |
| 17 | 90.06808 | 2,3-Butanediol |
| 17 | 230.01915 | D-Ribose 5-Phosphate |
| 17 | 182.07904 | Sorbitol |
| 18 | 167.05824 | 4-Hydroxy-L-Phenylglycine |
| 18 | 147.05316 | N-Acetylserine |
| 18 | 404.00220 | Uridine 5'-Diphosphate |
| 18 | 224.07971 | Hydroxykynurenine |
| 18 | 179.07937 | Galactosamine |
| 18 | 491.00083 | Deoxyadenosine Triphosphate |
| 18 | 172.01367 | Glycerol 3-Phosphate |
| 18 | 146.05791 | Methyglutarate |
| 18 | 205.03751 | Xanthurenate |
| 18 | 72.05751 | 2-Methylpropanal |
| 18 | 295.10559 | INDOXYL B-GLUCOSIDE |
| 18 | 112.05243 | Sorbate |
| 18 | 132.04226 | Monoethylmalonate |
| 18 | 178.04774 | Gluconolactone |
| 18 | 138.03169 | 4-Hydroxybenzoate |
| 18 | 137.08406 | Tyramine |

| VIAL | MMI | PRIMARY NAME |
|---|---|---|
| 18 | 362.20932 | Cortisol |
| 18 | 86.07316 | Prenol |
| 18 | 122.03678 | 3-Hydroxybenzaldehyde |
| 18 | 342.11621 | Maltose |
| 19 | 59.07350 | Trimethylamine |
| 19 | 88.05243 | Isobutyrate |
| 19 | 124.05243 | 3-Hydroxybenzyl Alcohol |
| 19 | 93.05785 | Aniline |
| 19 | 232.12118 | Melatonin |
| 19 | 116.01096 | Maleate |
| 19 | 102.06808 | Pentanoate |
| 19 | 74.03678 | Propanoate |
| 19 | 584.26348 | Bilirubin |
| 19 | 161.10787 | Nicotine |
| 19 | 396.19705 | Pregnenolone Sulfate |
| 19 | 189.04259 | Kynurenate |
| 19 | 432.86718 | 3,5-Diiodo-L-Tyrosine |
| 19 | 152.04734 | Mandelate |
| 19 | 160.10005 | Tryptamine |
| 19 | 137.04768 | 4-Aminobenzoate |
| 19 | 132.04226 | Glutarate |
| 19 | 100.05243 | 5-Valerolactone |
| 19 | 180.04226 | Caffeate |
| 19 | 242.08038 | Lumichrome |
| 19 | 178.04774 | L-Gulonolactone |
| 20 | 88.05243 | Acetoin |
| 20 | 89.04768 | Beta-Alanine |
| 20 | 207.08954 | N-Acetylphenylalanine |
| 20 | 157.07389 | N-Acetylproline |
| 20 | 203.10586 | L-Tryptophanamide |
| 20 | 137.08406 | Phenylethanolamine |
| 20 | 166.03132 | Thiopurine S-Methylether |
| 20 | 150.03506 | 2-Hydroxy-4-(Methylthio)Butanoate |
| 20 | 94.04186 | Phenol |
| 20 | 174.11570 | N-Methyltryptamine |
| 20 | 132.00587 | Oxaloacetate |
| 20 | 154.02661 | 2,3-Dihydroxybenzoate |
| 20 | 72.02113 | 2-Propenoate |
| 20 | 161.08406 | Indole-3-Ethanol |
| 20 | 194.05791 | Ferulate |
| 20 | 465.30904 | Glycocholate |
| 20 | 449.31412 | Glycochenodeoxycholate |
| 20 | 122.03678 | Benzoate |
| 20 | 153.04259 | 3-Amino-5-Hydroxybenzoate |
| 20 | 110.03678 | Pyrocatechol |
| 20 | 180.06339 | Tagatose |
| 21 | 188.14125 | 10-Hydroxydecanoate |
| 21 | 565.37436 | Didecanoyl-Glycerophosphocholine |
| 21 | 95.03711 | 2-Hydroxypyridine |
| 21 | 154.02661 | 3,4-Dihydroxybenzoate |
| 21 | 84.05751 | Cyclopentanone |
| 21 | 130.06299 | Pantolactone |
| 21 | 124.05243 | Guaiacol |
| 21 | 152.04734 | 2-Hydroxyphenylacetate |
| 21 | 168.04226 | 3,4-Dihydroxyphenylacetate |
| 21 | 175.06333 | Indole-3-Acetate |
| 21 | 404.21989 | Cortisol 21-Acetate |
| 21 | 174.07931 | Indole-3-Acetamide |
| 21 | 203.11710 | N6-(Delta2-Isopentenyl)-Adenine |
| 21 | 182.05791 | Methyl Vanillate |
| 21 | 102.03169 | 2-Oxobutanoate |
| 21 | 205.05951 | Lipoamide |
| 21 | 153.04259 | 3-Hydroxyanthranilate |
| 21 | 180.04226 | 3-(4-Hydroxyphenyl)Pyruvate |
| 21 | 116.08373 | Hexanoate |

| VIAL | MMI | PRIMARY NAME |
|---|---|---|
| 21 | 118.02661 | Methylmalonate |
| 21 | 150.05282 | Arabinose |
| 22 | 179.05824 | Hippurate |
| 22 | 173.04768 | 2-Quinolinecarboxylate |
| 22 | 176.09496 | Serotonin |
| 22 | 163.04941 | Pterin |
| 22 | 132.04226 | Ethylmalonate |
| 22 | 524.89339 | 3,5-Diiodo-L-Thyronine |
| 22 | 116.01096 | Fumarate |
| 22 | 106.04186 | Benzaldehyde |
| 22 | 122.03678 | 4-Hydroxybenzaldehyde |
| 22 | 166.06299 | 3-(2-Hydroxyphenyl)Propanoate |
| 22 | 167.09463 | 3-Methoxytyramine |
| 22 | 107.07350 | Benzylamine |
| 22 | 88.05243 | Butanoate |
| 22 | 172.01367 | Beta-Glycerophosphate |
| 22 | 260.02972 | Glucose 1-Phosphate |
| 22 | 104.05858 | 2,3-Diaminopropionate |
| 22 | 109.05276 | 2-Aminophenol |
| 22 | 160.07356 | 6-Carboxyhexanoate |
| 22 | 203.05824 | Indole-3-Pyruvate |
| 22 | 348.03288 | Dehydroascorbate |
| 22 | 153.04259 | 3-Amino-4-Hydroxybenzoate |
| 22 | 184.03717 | 3,4 Dihydroxymandelate |
| 22 | 206.04265 | 2-Methylcitrate |
| 22 | 239.10184 | Dihydrobiopterin |
| 22 | 342.11621 | Cellobiose |
| 23 | 154.02661 | 2,5-Dihydroxybenzoate |
| 23 | 188.10486 | Azelate |
| 23 | 160.03718 | Oxoadipate |
| 23 | 146.05791 | 2-Methylglutarate |
| 23 | 173.04768 | 4-Quinolinecarboxylate |
| 23 | 110.03678 | Hydroquinone |
| 23 | 214.13174 | Dethiobiotin |
| 23 | 138.03169 | 3-Hydroxybenzoate |
| 23 | 86.07316 | 2-Methylbutanal |
| 23 | 218.10553 | N-Acetylserotonin |
| 23 | 182.05791 | Hydrophenyllactic Acid |
| 23 | 130.02661 | Itaconate |
| 23 | 120.05751 | Phenylacetaldehyde |
| 23 | 226.09536 | Porphobilinogen |
| 23 | 152.04734 | 2',4'-Dihydroxyacetophenone |
| 23 | 164.04734 | 4-Coumarate |
| 23 | 117.05785 | Indole |
| 23 | 202.08412 | Diethyl 2-Methyl-3-Oxosuccinate |
| 24 | 174.08921 | Suberate |
| 24 | 146.05791 | Adipate |
| 24 | 314.06843 | Geranyl-Pp |
| 24 | 130.06299 | 3-Methyl-2-Oxovalerate |
| 24 | 86.03678 | Diacetyl |
| 24 | 88.01604 | Pyruvate |
| 24 | 132.05751 | Trans-Cinnamaldehyde |
| 24 | 111.03203 | 2,6-Dihydroxypyridine |
| 24 | 152.04734 | Vanillin |
| 24 | 116.04734 | Methyl Acetoacetate |
| 24 | 173.10519 | N-Acetylleucine |
| 24 | 108.05751 | Benzyl Alcohol |
| 24 | 189.07898 | Indole-3-Methyl Acetate |
| 24 | 148.07356 | Mevalonate |
| 24 | 139.98746 | Acetylphosphate |
| 24 | 158.13068 | Nonanoate |
| 24 | 776.68668 | Thyroxine |
| 24 | 188.04735 | 1-Hydroxy-2-Naphthoate |
| 24 | 129.05785 | Quinoline |
| 25 | 122.07316 | 1-Phenylethanol |
| 25 | 179.09463 | Salsolinol |
| 25 | 137.04768 | Salicylamide |

| VIAL | MMI | PRIMARY NAME |
|---|---|---|
| 25 | 198.05282 | 3-Methoxy-4-Hydroxymandelate |
| 25 | 182.05791 | Homovanillate |
| 25 | 130.02661 | 2-Methylmaleate |
| 25 | 146.02152 | Oxoglutarate |
| 25 | 203.09463 | Ethyl 3-Indoleacetate |
| 25 | 136.10005 | N,N-Dimethyl-1,4-Phenylenediamine |
| 25 | 168.04226 | Homogentisate |
| 25 | 159.06841 | Indoleacetaldehyde |
| 25 | 184.07356 | 4-Hydroxy-3-Methoxyphenylglycol |
| 25 | 152.04734 | 3-Hydroxyphenylacetate |
| 25 | 124.05243 | 4-Methylcatechol |
| 25 | 144.11503 | Caprylate |
| 25 | 130.09938 | Heptanoate |
| 25 | 650.79004 | Liothyronine |
| 25 | 146.08440 | 5,6 Dimethylbenzimidazole |
| 25 | 164.04734 | Phenylpyruvate |
| 25 | 170.16707 | 2-Undecanone |
| 25 | 172.14633 | Decanoate |
| 26 | 146.05791 | Monomethylglutarate |
| 26 | 167.05824 | Pyridoxal |
| 26 | 138.03169 | Salicylate |
| 26 | 202.12051 | Sebacate |
| 26 | 147.06841 | 3-Methyl-2-Oxindole |
| 26 | 152.04735 | Resorcinol Monoacetate |
| 26 | 102.03169 | Acetoacetate |
| 26 | 149.07015 | 3-Methyladenine |
| 26 | 182.05791 | Hydroxyphenyllactate |
| 26 | 244.08816 | Biotin |
| 26 | 119.98811 | Mercaptopyruvate |
| 26 | 72.02113 | Pyruvic Aldehyde |
| 26 | 111.03203 | Pyrrole-2-Carboxylate |
| 26 | 191.05824 | 5-Hydroxyindoleacetate |
| 26 | 144.04226 | 3-Methylglutaconate |
| 26 | 200.17763 | Laurate |
| 26 | 130.06300 | Ketoleucine |
| 26 | 148.05243 | Trans-Cinnamate |
| 27 | 911.15748 | 3-Hydroxy-3-Methylglutaryl-Coa |
| 27 | 849.15707 | 3-Methylcrotonyl-Coa |
| 27 | 884.28006 | Cytochrome C |
| 27 | 1355.57523 | Cyanocobalamin |
| 27 | 366.24063 | 3-Alpha,11-Beta,17,21-Tetrahydroxy- 5-Beta-Pregnan-20-One |
| 27 | 396.33922 | Vitamin D2 |
| 27 | 346.21441 | Corticosterone |
| 27 | 376.29775 | Lithocholate |
| 27 | 562.25801 | Protoporphyrin |
| 27 | 410.39125 | Squalene |
| 27 | 272.17763 | Estradiol-17alpha |
| 27 | 392.29266 | Ursodeoxycholate |
| 27 | 282.25588 | Petroselinate |
| 27 | 568.50668 | Dipalmitoylglycerol |
| 27 | 483.30184 | Lithocholyltaurine |
| 27 | 286.22967 | Retinol |
| 27 | 444.30280 | Menaquinone |
| 27 | 228.20893 | Myristate |
| 27 | 650.60018 | Cholesteryl Oleate |
| 27 | 360.08452 | Rosmarinate |
| 27 | 806.73634 | Glyceryl Tripalmitate |
| 28 | 346.21441 | Cortexolone |
| 28 | 254.22458 | Palmitoleate |
| 28 | 282.25588 | Elaidate |
| 28 | 272.17763 | Chenodeoxycholate |
| 28 | 256.24023 | Palmitate |
| 28 | 302.22458 | Eicosapentaenoate |

| VIAL | MMI | PRIMARY NAME |
|---|---|---|
| 28 | 270.25588 | Heptadecanoate |
| 28 | 722.64244 | Glyceryl Trimyristate |
| 28 | 301.29808 | Sphinganine |
| 28 | 426.38617 | Lanosterol |
| 28 | 312.30283 | Arachidate |
| 28 | 338.31848 | Erucate |
| 28 | 392.29266 | Deoxycholate |
| 28 | 280.24023 | Linoleate |
| 28 | 730.60670 | Sphingomyelin |
| 28 | 384.33922 | 7-Dehydrocholesterol |
| 28 | 390.27701 | Bis(2-Ethylhexyl)Phthalate |
| 28 | 278.22458 | Gamma-Linolenate |
| 28 | 216.17254 | Omega-Hydroxydodecanoate |
| 28 | 224.14124 | Methyl Jasmonate |
| 28 | 733.56216 | Dipalmitoyl-Phosphatidylcholine |
| 29 | 242.26097 | Hexadecanol |
| 29 | 300.20893 | Retinoate |
| 29 | 691.51520 | Dipalmitoyl-Phosphoethanolamine |
| 29 | 408.28757 | Cholate |
| 29 | 450.34978 | Phylloquinone |
| 29 | 624.58453 | Cholesteryl Palmitate |
| 29 | 328.24023 | Docosahexaenoate |
| 29 | 524.45933 | Retinyl Palmitate |
| 29 | 430.38108 | Alpha-Tocopherol |
| 29 | 302.24571 | Glycerol-Myristate |
| 29 | 354.34978 | Tricosanoate |
| 29 | 282.25588 | Oleate |
| 29 | 284.27153 | Stearate |
| 29 | 536.43820 | Beta-Carotene |
| 29 | 402.34978 | 25-Hydroxycholesterol |
| 29 | 366.34978 | Nervonate |
| 29 | 384.33922 | Desmosterol |
| 29 | 372.23006 | Deoxycorticosterone Acetate |
| 29 | 356.29266 | Oleoyl-Glycerol |
| 29 | 862.68391 | Coenzyme Q10 |
| 29 | 360.19367 | Cortisone |

**Table S3**. Final in-house built library, indicating retention times and most abundant adduct (positive or negative) for each metabolite. Except for lipids panel (see paragraph 2.6.2), all the other metabolites were analysed with the conditions reported in paragraph 2.6.1.

| PRIMARY NAME | ADDUCT | RT |
|---|---|---|
| | **ESI+** | |
| (2-Aminoethyl)Phosphonate | 126.031 | 0.68 |
| 1-Aminocyclopropanecarboxylic acid | 102.054 | 0.74 |
| 1-Methyladenosine | 282.122 | 5.64 |
| 1-Methyl-L-Histidine | 170.092 | 0.66 |
| 1-Methylnicotinamide | 137.071 | 0.73 |
| 1-Phenylethanol | 123.079 | 10.07 |
| 2,3-Butanediol | 135.042 | 1.44 |
| 2',4'-Dihydroxyacetophenone | 153.055 | 8.99 |
| 2,4-Dihydroxypteridine | 165.040 | 3.3 |
| 2,5-Dimethylpyrazine | 126.101 | 0.58 |
| 2,6-Dihydroxypyridine | 112.038 | 1.72 |
| 2-Acetamido-2-Deoxy-Beta-D-Glucosylamine | 204.086 | 0.82 |
| 2-Aminoisobutyrate | 104.069 | 0.79 |
| 2'-Deoxyguanosine 5'-monophosphate | 348.070 | 1.01 |
| 2-Hydroxypyridine | 96.042 | 2.97 |
| 2-Methylpropanal | 95.048 | 0.77 |
| 2-Phosphoglycerate | 204.026 | 1.01 |
| 2-Undecanone | 193.160 | 10.78 |
| 3-(4-Hydroxyphenyl)Pyruvate | 163.038 | 10.59 |
| 3,5-Diiodo-L-Thyronine | 215.139 | 8.82 |
| 3,5-Diiodo-L-Tyrosine | 433.876 | 8.3 |
| 3-Amino-4-Hydroxybenzoate | 154.050 | 1.03 |
| 3-Aminoisobutanoic acid | 104.069 | 0.8 |
| 3-Hydroxybenzaldehyde | 105.032 | 11.34 |
| 3-Hydroxybenzenemethanol | 107.048 | 6.14 |
| 3-Hydroxyphenylacetate | 151.040 | 7.81 |
| 3-Methoxytyramine | 151.076 | 3.84 |
| 3-Methoxytyrosine | 212.091 | 4.35 |
| 3-Methyl-2-Oxindole | 148.076 | 9.42 |
| 3-Methylhistamine | 126.102 | 0.57 |
| 3-Methyl-L-Histidine | 170.093 | 0.63 |
| 3-Sulfinoalanine | 154.016 | 0.71 |
| 4-Aminobenzoate | 138.055 | 5.01 |
| 4-Guanidinobutanoate | 146.091 | 1.02 |
| 4-Hydroxy-3-Methoxyphenylglycol | 167.070 | 4.64 |
| 4-Hydroxy-L-Phenylglycine | 151.036 | 0.77 |
| 4-Imidazoleacetate | 127.050 | 0.8 |
| 4-Methylcatechol | 107.048 | 7.85 |
| 4-Pyridoxate | 184.061 | 4.08 |
| 5-Aminolevulinate | 114.054 | 0.73 |
| 5-Aminopentanoate | 101.059 | 0.84 |
| 5'-Deoxyadenosine | 252.109 | 5 |
| 5-Hydroxyindoleacetate | 192.066 | 7.07 |
| 5-Hydroxy-L-Tryptophan | 204.066 | 4.32 |
| 5-Methylcytosine | 126.066 | 0.85 |
| 6-Hydroxydopamine | 153.053 | 3.69 |
| Acetylcholine | 146.116 | 0.85 |
| Adenine | 136.061 | 1 |
| Adenosine | 268.104 | 4.07 |
| Adenosine 2',3'-cyclic phosphate | 330.061 | 2.95 |
| Adenosine 3',5'-Diphosphate | 428.034 | 1.21 |
| Adenosine Diphosphate Ribose | 560.071 | 1.19 |
| Adenosine-Monophosphate | 348.072 | 1.02 |
| Agmatine | 131.128 | 0.61 |
| AICAR | 339.068 | 1.03 |
| Allantoin | 159.049 | 0.77 |
| Alpha-D-Glucose | 203.052 | 0.73 |
| Aniline | 77.037 | 11.34 |
| Anserine | 241.130 | 0.66 |
| Arabinose | 173.040 | 0.76 |
| Arginine | 175.119 | 0.66 |
| Benzaldehyde | 91.053 | 4.3 |
| Benzyl Alcohol | 109.063 | 10.06 |
| Beta-Alanine | 90.054 | 0.67 |
| Betaine | 118.086 | 0.73 |
| Beta-Nicotinamide Adenine Dinucleotide | 332.562 | 1.03 |
| Biliverdin | 292.139 | 1.12 |
| Biotin | 245.097 | 8.14 |
| Cadaverine | 89.095 | 0.58 |
| Caffeine | 195.088 | 7.9 |
| CAMP | 330.060 | 4.56 |
| Carnosine | 227.114 | 0.65 |
| CDP-Ethanolamine | 447.065 | 0.79 |
| Choline | 60.080 | 104.107 |
| Cis-4-Hydroxy-D-Proline | 132.066 | 0.69 |
| Citicoline | 489.114 | 0.81 |
| Citrulline | 159.077 | 0.75 |
| Cortisol | 363.219 | 10.34 |
| Cortisol 21-Acetate | 405.230 | 10.74 |
| Creatine | 132.077 | 0.8 |
| Creatinine | 114.066 | 0.75 |
| Cyclic GMP | 346.055 | 4.64 |
| Cys-Gly | 179.048 | 0.66 |
| Cystine | 241.031 | 0.67 |
| Cytidine 2',3'-Cyclic Phosphate | 306.048 | 0.85 |
| Cytidine Diphosphate | 202.518 | 0.83 |
| Cytidine Monophosphate | 324.059 | 0.84 |
| Cytosine | 112.049 | 0.77 |
| dCDP | 388.035 | 0.84 |
| delta-Trimethyllysine | 189.160 | 0.63 |
| Deoxyadenosine | 252.109 | 3.57 |
| Deoxyadenosine Monophosphate | 332.075 | 1.03 |
| Deoxycarnitine | 146.118 | 0.8 |
| Deoxycytidine | 250.079 | 1.03 |
| Deoxycytidine Monophosphate | 308.062 | 1.03 |
| Deoxyguanosine | 290.086 | 4.66 |
| Deoxyribose | 157.045 | 0.9 |
| Deoxyuridine | 251.064 | 3.78 |

| PRIMARY NAME | ADDUCT | RT |
|---|---|---|
| Deoxyuridine Triphosphate | 451.964 | 2.95 |
| Dethiobiotin | 215.139 | 8.82 |
| dGTP | 525.040 | 2.74 |
| Diaminopimelate | 191.102 | 0.66 |
| Didecanoyl-Glycerophosphocholine | 588.365 | 14.18 |
| Diethanolamine | 106.085 | 0.67 |
| Dihydroorotate | 159.039 | 0.98 |
| Dihydrouracil | 115.049 | 1.03 |
| Dimethylbenzimidazole | 147.093 | 7.95 |
| D-Mannosamine | 162.075 | 0.65 |
| Dopamine | 118.060 | 4.34 |
| D-Ornithine | 116.070 | 0.61 |
| D-Pantolactone | 175.034 | 6.55 |
| D-Pinitol | 217.069 | 0.73 |
| D-Ribose 5-Phosphate | 166.086 | 0.86 |
| Dtdp-D-Glucose | 547.046 | 0.83 |
| Epinephrine | 166.086 | 0.86 |
| Erythritol | 145.046 | 0.75 |
| Ethanolamine | 84.079 | 1.03 |
| Ethyl 3-Indoleacetate | 226.084 | 10.46 |
| Fucose | 187.057 | 0.91 |
| Galactosamine | 202.069 | 0.63 |
| Galactose | 203.052 | 0.69 |
| Gamma-Aminobutyrate | 104.070 | 0.71 |
| Glucosaminate | 196.081 | 0.68 |
| Glucosamine | 162.076 | 0.65 |
| Glucosamine 6-Phosphate | 260.052 | 0.66 |
| Glucosamine 6-Sulfate | 260.043 | 0.68 |
| Glutamine | 130.490 | 0.69 |
| Glutarylcarnitine | 276.146 | 3.84 |
| Glycerol | 115.035 | 0.78 |
| Glycine | 76.040 | 0.7 |
| Guaiacol | 107.047 | 6.55 |
| Guanidinoacetate | 118.061 | 0.72 |
| Guanidinosuccinate | 176.067 | 0.76 |
| Guanine | 152.056 | 4.36 |
| Guanosine | 306.081 | 4.36 |
| Guanosine Diphosphate Mannose | 606.081 | 1.9 |
| Gulose | 203.053 | 0.72 |
| Histamine | 112.086 | 0.59 |
| Histidine | 156.076 | 0.64 |
| Histidinol | 142.096 | 0.58 |
| Homocysteine | 136.042 | 0.85 |
| Homocysteine Thiolactone | 118.031 | 0.85 |
| Homocystine | 269.062 | 0.86 |
| Homoserine | 120.065 | 0.69 |
| Hypotaurine | 110.024 | 0.71 |
| Hypoxanthine | 137.045 | 1.04 |
| Indole-3-Ethanol | 144.080 | 9.14 |
| Indole-3-Methyl Acetate | 212.069 | 9.97 |
| Indole-3-Pyruvate | 187.040 | 2.95 |
| Indoleacetaldehyde | 160.074 | 6.06 |
| Inosine | 291.071 | 4.32 |
| Isoleucine | 132.101 | 1.72 |
| Kynurenine | 209.092 | 4.34 |
| Lactose | 365.106 | 0.77 |
| L-Alanine | 90.054 | 0.7 |
| Lauroylcarnitine | 344.280 | 11.09 |
| L-Carnitine | 162.112 | 0.72 |
| L-Dopa | 198.076 | 1.03 |

| PRIMARY NAME | ADDUCT | RT |
|---|---|---|
| Leucine | 132.101 | 2.41 |
| Liothyronine | 651.800 | 10.34 |
| Lipoamide | 228.049 | 9.62 |
| L-Ornithine | 116.070 | 0.6 |
| Lumichrome | 243.089 | 9.71 |
| Lysine | 147.112 | 0.62 |
| Mannose | 203.052 | 0.73 |
| Melatonin | 233.130 | 9.06 |
| Methionine | 150.058 | 1.01 |
| Methionine Sulfoximine | 181.064 | 0.68 |
| Methyl vanillic acid | 183.060 | 9.39 |
| Methylguanidine | 74.070 | 0.75 |
| Methylthioadenosine | 298.099 | 6.9 |
| Mevalonolactone | 153.053 | 3.69 |
| Monoethyl malonic acid | 150.077 | 1 |
| N,N-Dimethyl-1,4-Phenylenediamine | 137.107 | 1.91 |
| N,N-Dimethylarginine | 203.150 | 0.86 |
| N1-Acetylspermine | 245.234 | 0.54 |
| N6-(Delta2-Isopentenyl)-Adenine | 204.122 | 8.74 |
| N-Acetylalanine | 154.046 | 2.35 |
| N-Acetylgalactosamine | 244.079 | 0.7 |
| N-Acetylglucosamine | 244.079 | 0.82 |
| N-Acetylglycine | 118.048 | 1.04 |
| N-Acetylmannosamine | 244.081 | 0.84 |
| N-Acetylmethionine | 214.050 | 6.59 |
| N-Acetylproline | 180.063 | 5.62 |
| N-Acetylputrescine | 131.117 | 0.84 |
| N-Acetylserotonin | 241.096 | 7.43 |
| N-Acetyltryptophan | 247.109 | 8.9 |
| N-Alpha-Acetyllysine | 189.123 | 0.84 |
| N-Formylglycine | 147.995 | 0.91 |
| Nicotinamide | 123.055 | 1.03 |
| Nicotinamide Hypoxanthine Dinucleotide | 665.095 | 1.04 |
| Nicotine | 161.105 | 6.5 |
| N-Methyl-4-aminobutyric acid | 101.058 | 1.03 |
| N-Methylalanine | 104.070 | 0.75 |
| N-Methyltryptamine | 175.120 | 8.03 |
| Noradrenaline | 152.070 | 0.78 |
| Norleucine | 132.101 | 2.6 |
| Normetanephrine | 166.086 | 1 |
| Norspermidine | 132.149 | 0.54 |
| Norvaline | 118.086 | 1.02 |
| O-Acetylcarnitine | 204.123 | 1.01 |
| O-Acetylserine | 130.048 | 1.79 |
| O-Phosphoethanolamine | 142.024 | 0.68 |
| O-Phosphoserine | 186.015 | 0.71 |
| O-Succinyl-Homoserine | 220.081 | 1.53 |
| Oxoproline | 130.049 | 1.77 |
| Palmitoylcarnitine | 400.342 | 12.31 |
| Paraxanthine | 181.072 | 6.83 |
| Phenol | 112.074 | 5.62 |
| Phenylalanine | 166.086 | 4.76 |
| Phenylethanolamine | 120.080 | 8.83 |
| Phosphoribosyl Pyrophosphate | 412.934 | 0.71 |
| Phosphorylcholine | 184.074 | 0.7 |
| Phosphoserine | 188.016 | 0.71 |
| Picolinate | 124.038 | 1.03 |
| Pipecolate | 130.085 | 1.03 |
| P-Octopamine | 136.075 | 0.84 |

| PRIMARY NAME | ADDUCT | RT |
|---|---|---|
| Prenol | 104.105 | 0.7 |
| Proline | 116.070 | 0.8 |
| Propanoate | 97.026 | 0.75 |
| Psicose | 203.053 | 0.8 |
| Pterin | 164.057 | 2.95 |
| Purine | 121.050 | 1.76 |
| Putrescine | 72.080 | 0.57 |
| Pyridoxal | 168.064 | 1.01 |
| Pyridoxamine | 169.096 | 0.7 |
| Pyridoxine | 170.081 | 2.06 |
| Pyruvate | 106.049 | 0.62 |
| Quinoline | 130.066 | 9.16 |
| Resorcinol Monoacetate | 175.033 | 7.28 |
| Riboflavin | 377.146 | 8.47 |
| Ribose | 173.042 | 0.76 |
| S-Adenosylhomocysteine | 385.128 | 3.73 |
| S-Adenosylmethionine | 399.144 | 0.73 |
| Salicylamide | 138.055 | 7.95 |
| Salsolinol | 180.102 | 2.42 |
| Sarcosine | 90.054 | 0.7 |
| Selenocystamine | 248.935 | 0.64 |
| Serine | 106.049 | 0.68 |
| Serotonin | 160.076 | 3.45 |
| Sorbose | 203.051 | 0.73 |
| Spermidine | 146.164 | 0.52 |
| Spermine | 203.223 | 0.5 |
| Tagatose | 203.052 | 0.77 |
| Taurine | 126.021 | 0.7 |
| Theobromine | 181.072 | 6.11 |
| Theophylline | 181.072 | 7.03 |
| Thiamine | 266.116 | 0.77 |
| Thiamine Monophosphate | 345.078 | 0.74 |
| Thiamine Pyrophosphate | 345.041 | 3.22 |
| Thiopurine S-Methylether | 167.038 | 7.96 |
| Thiourea | 77.0160 | 0.82 |
| Threitol | 145.045 | 0.75 |
| Threonine | 120.065 | 0.71 |
| Thymidine | 265.080 | 5.41 |
| Thymine | 127.049 | 2.59 |
| Thyroxine | 196.095 | 8.6 |
| Trans-4-Hydroxy-L-Proline | 132.065 | 0.7 |
| Trans-Cinnamaldehyde | 115.053 | 11.09 |
| TRH | 363.177 | 2.26 |
| Trigonelline | 138.055 | 0.8 |
| Trimethylamine | 60.080 | 0.69 |
| Tryptamine | 144.081 | 6.5 |
| Tryptophan | 188.070 | 6.42 |
| Tyramine | 121.064 | 1.71 |
| Tyrosine | 182.080 | 2.4 |
| Uracil | 113.030 | 1.03 |
| Ureidopropionate | 115.048 | 1.02 |
| Uridine | 267.058 | 2.56 |
| Uridine Monophosphate | 325.042 | 1.03 |
| Valine | 118.085 | 0.73 |
| Vanillin | 175.034 | 6.79 |
| Xanthine | 153.040 | 1.29 |
| Xylose | 151.058 | 1.49 |

| PRIMARY NAME | ADDUCT | RT |
|---|---|---|
| | **ESI-** | |
| 10-Hydroxydecanoate | 187.134 | 10.3 |
| 1-Hydroxy-2-Naphthoate | 187.041 | 11.09 |
| 2,3-Diaminopropionate | 103.051 | 0.62 |
| 2,3-Dihydroxybenzoate | 153.020 | 7.71 |
| 2,5-Dihydroxybenzoate | 153.019 | 6.72 |
| 2-Deoxyglucose | 181.073 | 0.71 |
| 2-Hydroxy-4-(methylthio) butanoic acid | 149.030 | 5.78 |
| 2-Hydroxybutyrate | 103.040 | 2.67 |
| 2-Hydroxyphenylacetate | 151.042 | 6.5 |
| 2-Keto-3-Deoxy-D-Gluconic Acid | 177.040 | 0.82 |
| 2-Methylglutarate | 145.051 | 6.47 |
| 2-Propenoate | 89.0240 | 1 |
| 2-Quinolinecarboxylate | 172.041 | 7.39 |
| 3-(2-Hydroxyphenyl)Propanoate | 165.056 | 8.98 |
| 3,4 Dihydroxymandelate | 183.029 | 1.48 |
| 3,4-Dihydroxybenzoate | 153.020 | 5.41 |
| 3,4-Dihydroxyphenylacetate | 167.036 | 6.2 |
| 3,4-Dihydroxyphenylglycol | 151.040 | 1.82 |
| 3-Amino-5-Hydroxybenzoate | 152.036 | 1.56 |
| 3-Dehydroshikimate | 171.030 | 1.01 |
| 3-Hydroxyanthranilate | 152.036 | 5.15 |
| 3-Hydroxybenzoate | 137.025 | 7.78 |
| 3-Hydroxybutanoate | 102.945 | 0.54 |
| 3-Hydroxymethylglutarate | 161.046 | 2.88 |
| 3-Methoxy-4-Hydroxymandelate | 197.045 | 3.8 |
| 3-Methyl-2-Oxovalerate | 147.067 | 2.59 |
| 3-Methylglutaconate | 143.034 | 6.53 |
| 3-Nitro-L-Tyrosine | 225.052 | 5.45 |
| 4-Acetamidobutanoate | 144.068 | 3.55 |
| 4-Coumarate | 163.041 | 8.63 |
| 4-Hydroxybenzaldehyde | 121.030 | 7.63 |
| 4-Hydroxybenzoate | 137.026 | 6.92 |
| 4-Quinolinecarboxylic acid | 172.041 | 3.2 |
| 5-Hydroxylysine | 161.094 | 0.59 |
| 5-Thymidylic acid | 321.048 | 3.2 |
| 6-Carboxyhexanoate | 159.067 | 8 |
| 6-Hydroxynicotinate | 138.020 | 3.59 |
| 6-Phosphogluconate | 275.016 | 0.83 |
| Adipate | 145.051 | 6.26 |
| Adp-Glucose | 588.075 | 1.01 |
| Allose | 178.980 | 0.58 |
| Alpha-Hydroxyisobutyrate | 127.074 | 0.7 |
| Aminoadipate | 160.063 | 0.84 |
| Aniline-2-Sulfonate | 172.008 | 2.16 |
| Arabitol | 151.062 | 0.74 |
| Ascorbate | 174.954 | 0.58 |
| Asparagine | 131.046 | 0.67 |
| Aspartate | 132.031 | 0.69 |
| Azelate | 187.097 | 9.64 |
| Benzoate | 121.029 | 9.19 |
| Beta-Glycerophosphate | 171.007 | 0.76 |
| Butanoate | 87.046 | 5.11 |
| Caffeate | 179.036 | 7.87 |
| Caprylate | 125.097 | 9.66 |
| Cellobiose | 341.109 | 0.81 |
| Citramalate | 147.030 | 2.16 |
| Citrate | 191.020 | 1.57 |
| Cystathionine | 221.061 | 0.67 |
| Cysteate | 167.998 | 0.7 |

| PRIMARY NAME | ADDUCT | RT |
|---|---|---|
| Cysteine | 120.012 | 0.67 |
| Cytidine | 242.078 | 1.02 |
| D-Alanine | 88.040 | 0.68 |
| Deoxyuridine-Monophosphate | 307.034 | 1.64 |
| D-Glucuronolactone | 193.037 | 0.71 |
| D-Glyceraldehyde 3-phosphate | 168.990 | 0.74 |
| Dihydrobiopterin | 220.084 | 7.52 |
| Dihydrofolate | 442.147 | 7.68 |
| Dihydroxyfumarate | 146.939 | 0.55 |
| D-Ribose 5-Phosphate | 229.011 | 0.73 |
| Ethyl 3-Ureidopropionate | 158.978 | 0.77 |
| Ethylmalonate | 131.035 | 5.11 |
| FAD | 784.148 | 7.92 |
| Ferulate | 193.052 | 8.85 |
| Folate | 440.131 | 7.76 |
| Fructose 6-Phosphate | 259.022 | 0.72 |
| Fumarate | 115.004 | 1.81 |
| Galactarate | 209.030 | 0.74 |
| Galactitol | 181.073 | 0.71 |
| Galactose 1-Phosphate | 259.023 | 0.72 |
| Galacturonate | 193.036 | 0.73 |
| Gentisic acid | 153.020 | 6.69 |
| Geranyl-PP | 313.088 | 6.26 |
| Gluconate | 195.053 | 0.73 |
| Gluconolactone | 195.052 | 0.74 |
| Glucose 1-Phosphate | 259.022 | 0.72 |
| Glucose 6-Phosphate | 259.022 | 0.72 |
| Glucuronate | 193.037 | 0.71 |
| Glutamate | 146.046 | 0.72 |
| Glutarate | 131.036 | 4.08 |
| Gluthatione | 306.074 | 1.02 |
| Glyceraldehyde | 89.025 | 1.02 |
| Glycerate | 105.019 | 0.81 |
| Glycerol 3-Phosphate | 171.008 | 0.72 |
| Glycine | 74.025 | 0.72 |
| Glycochenodeoxycholate | 448.307 | 12.08 |
| Glycocholate | 464.302 | 11.54 |
| Guanosine Monophosphate | 362.048 | 1.66 |
| Hippurate | 178.051 | 7.32 |
| Homogentisate | 167.035 | 4.6 |
| Homovanillate | 181.050 | 8 |
| Hydroxyphenyllactate | 181.050 | 6.59 |
| Hydroxyphenyllactic Acid | 181.051 | 6.59 |
| Hydroxypyruvate | 102.948 | 0.54 |
| Indole-3-Acetamide | 173.073 | 8 |
| Indole-3-Acetate | 174.057 | 9.14 |
| Indoxyl Sulfate | 212.002 | 6.1 |
| Inosinic acid | 347.038 | 1.65 |
| Isobutyrate | 87.046 | 4.08 |
| Isocitrate | 191.020 | 1.6 |
| Isopentenyl Pyrophosphate | 245.0416 | 0.73 |
| Itaconate | 129.019 | 4.57 |
| Ketoleucine | 129.057 | 7.19 |
| Kynurenate | 188.037 | 7.54 |
| Lactate | 89.025 | 1.04 |
| L-Allothreonine | 118.052 | 0.68 |
| Laurate | 199.169 | 13.02 |
| L-Gulonolactone | 177.042 | 0.76 |
| L-Tryptophanamide | 202.099 | 5.55 |
| Lyxose | 131.035 | 0.71 |
| Malate | 133.015 | 0.96 |

| PRIMARY NAME | ADDUCT | RT |
|---|---|---|
| Maleate | 115.004 | 1.47 |
| Malonate | 103.044 | 1 |
| Maltose | 341.110 | 0.8 |
| Mandelate | 151.042 | 6.52 |
| Mannitol | 181.072 | 0.73 |
| Mannose 6-Phosphate | 259.022 | 0.72 |
| Melibiose | 341.108 | 0.77 |
| Meso-Tartrate | 149.009 | 0.78 |
| Methyglutarate | 145.052 | 6.29 |
| Methyl Galactoside | 193.070 | 0.93 |
| Methylmalonate | 117.020 | 2.37 |
| Mevalonate | 147.067 | 2.59 |
| Monomethyl glutaric acid | 145.053 | 7.07 |
| Myoinositol | 179.056 | 0.68 |
| N-Acetylasparagine | 173.058 | 0.97 |
| N-Acetylaspartate | 174.041 | 1.03 |
| N-Acetylcysteine | 162.023 | 6.12 |
| N-Acetylglutamate | 188.057 | 2.05 |
| N-Acetylleucine | 172.099 | 8.6 |
| N-Acetylneuraminate | 308.099 | 0.79 |
| N-Acetylphenylalanine | 206.083 | 8.80 |
| N-Acetylserine | 146.047 | 1 |
| N-Formyl-L-Methionine | 176.039 | 5.88 |
| Nicotinate | 122.025 | 1.02 |
| N-Methylaspartate | 146.046 | 0.72 |
| N-methyl-L-glutamic Acid | 160.061 | 0.77 |
| Nonanoate | 157.012 | 1.02 |
| Ophthalmate | 288.119 | 1.87 |
| Orotate | 155.010 | 1.01 |
| Oxalate | 89.020 | 1 |
| Oxaloacetate | 112.986 | 0.62 |
| Oxoadipate | 159.030 | 1.98 |
| Oxoglutarate | 145.015 | 1.03 |
| Palatinose | 341.106 | 0.81 |
| Pantothenate | 218.105 | 5.82 |
| Phenylacetaldehyde | 119.051 | 8.63 |
| Phenylpyruvate | 181.052 | 6.6 |
| Phosphonoacetate | 138.980 | 0.84 |
| P-Hydroxyphenylacetate | 151.040 | 7.46 |
| Porphobilinogen | 225.088 | 4.81 |
| Pregnenolone Sulfate | 395.191 | 11.44 |
| Pyridoxal 5'-phosphate | 246.017 | 1.93 |
| Pyrocatechol | 109.030 | 7.7 |
| Pyroglutamate | 128.036 | 1.79 |
| Pyrrole-2-Carboxylate | 110.025 | 5.82 |
| Quinate | 191.057 | 0.83 |
| Quinolinate | 166.015 | 1.5 |
| Raffinose | 503.156 | 1.02 |
| Rhamnose | 181.072 | 0.73 |
| Ribitol | 151.062 | 0.73 |
| Ribose 1,5-Bisphosphate | 308.861 | 0.55 |
| Saccharate | 209.031 | 0.75 |
| Salicylate | 137.025 | 9.33 |
| S-Carboxymethylcysteine | 178.018 | 0.82 |
| Sebacate | 201.113 | 10.17 |
| Sedoheptulose | 209.065 | 0.73 |
| Shikimate | 173.047 | 1.02 |
| Sorbate | 93.036 | 6.92 |
| Sorbitol | 181.071 | 0.73 |
| Stachyose | 665.213 | 0.96 |
| Suberate | 173.082 | 8.98 |

| PRIMARY NAME | ADDUCT | RT |
|---|---|---|
| Succinate | 117.019 | 2.03 |
| Sucrose | 341.110 | 0.84 |
| Tartrate | 149.010 | 0.84 |
| Trans-Aconitate | 191.020 | 1.57 |
| Trans-Cinnamate | 147.046 | 9.97 |
| Trehalose | 341.109 | 0.8 |
| Uracil 5-carboxylic acid | 155.010 | 1.95 |
| Urate | 167.022 | 1.02 |
| Uridine Diphosphate Glucose | 565.046 | 0.98 |
| Uridine Diphosphategalactose | 322.045 | 0.95 |
| Uridine Diphosphate-N-Acetylgalactosamine | 606.074 | 1 |
| Uridine Diphosphate-N-Acetylglucosamine | 606.069 | 1 |
| Urocanate | 139.050 | 1.02 |
| Uroxanthin | 294.100 | 6.88 |
| Xanthosine | 283.068 | 5.09 |
| Xanthurenate | 204.032 | 7.33 |
| Xanthylic acid | 363.034 | 1.96 |
| Xylitol | 151.062 | 0.74 |

| PRIMARY NAME | ADDUCT | RT |
|---|---|---|
| **LIPIDS** | **ESI+** | |
| 25-Hydroxycholesterol | 385.346 | 14.30 |
| 3-Hydroxy-3-Methylglutaryl-CoA | 912.164 | 4.46 |
| 5alpha-Tetrahydrocortisol | 389.229 | 0.80 |
| 7-Dehydrocholesterol | 385.346 | 14.26 |
| Alpha-Tocopherol | 431.388 | 14.76 |
| Beta-Carotene | 537.445 | 17.79 |
| Bis(2-Ethylhexyl)Phthalate | 391.284 | 6.46 |
| Chenodeoxycholate | 410.326 | 1.60 |
| Cholate | 426.321 | 0.97 |
| Cholesterol | 369.350 | 13.81 |
| Coenzyme Q10 | 863.698 | 18.08 |
| Cortexolone | 347.221 | 0.83 |
| Corticosterone | 347.221 | 0.86 |
| Cortisone | 361.201 | 0.77 |
| Cyanocobalamin | 1356.583 | 0.63 |
| Deoxycholate | 410.326 | 1.60 |
| Deoxycorticosterone Acetate | 373.237 | 1.08 |
| Desmosterol | 367.336 | 10.82 |
| Estradiol-17Alpha | 255.174 | 0.98 |
| Lanosterol | 409.383 | 14.39 |
| Lithocholate | 359.294 | 2.60 |
| Lithocholyltaurine | 466.298 | 1.01 |
| Menaquinone | 445.310 | 12.40 |
| Methyl Jasmonate | 207.138 | 0.81 |
| Omega-Hydroxydodecanoate | 199.169 | 0.93 |
| Phylloquinone | 451.357 | 15.56 |
| Protoporphyrin | 563.265 | 3.99 |
| Retinoate | 301.216 | 3.96 |
| Rosmarinate | 383.073 | 0.69 |
| Sphinganine | 302.305 | 2.70 |
| Squalene | 433.380 | 4.81 |
| Ursodeoxycholate | 410.326 | 1.65 |
| Vitamin D2 | 397.346 | 11.58 |

**Table S4**. Table figuring all the detected metabolites (n=146). Concentrations are displayed as fold-change over time 0.

| Metabolites | 0 | 90 | MW | END |
|---|---|---|---|---|
| 1,3 Cyclohexanedione | 1.00 | 1.58 | 2.15 | 1.73 |
| 1,7-Dimethyluric acid | 1.00 | 5.84 | 5.29 | 3.30 |
| 1-Methyladenosine | 1.00 | 2.61 | 3.78 | 3.14 |
| 1-Methylhistidine | 1.00 | 0.81 | 1.25 | 1.04 |
| 1-Methylnicotinamide | 1.00 | 6.58 | 10.24 | 6.77 |
| 2'-Deoxyguanosine 5'-monophosphate | 1.00 | 0.01 | 0.01 | 0.01 |
| 2-Hydroxy-4-methylpentanoic acid | 1.00 | 0.71 | 1.22 | 0.78 |
| 2-Phenylacetamide | 1.00 | 1.59 | 1.52 | 1.29 |
| 3-(1-Pyrazolyl)-alanine | 1.00 | 3.94 | 3.67 | 3.21 |
| 3-(2-Hydroxyphenyl)propanoic acid | 1.00 | 1.24 | 1.46 | 0.99 |
| 3-Hydroxybenzaldehyde | 1.00 | 1.76 | 1.54 | 1.66 |
| 3-Indoleacetic acid | 1.00 | 2.18 | 4.04 | 2.77 |
| 3-Indolepropionic acid | 1.00 | 1.97 | 1.85 | 1.53 |
| 3-Methoxytyrosine | 1.00 | 2.00 | 2.15 | 2.17 |
| 4-Aminophenol | 1.00 | 1.54 | 194.09 | 1.85 |
| 4-Guanidinobutanoate | 1.00 | 2.94 | 2.93 | 2.57 |
| 4-Hydroxyhippuric acid | 1.00 | 79.93 | 5.61 | 10.68 |
| 4-Hydroxyphenylglycine | 1.00 | 1.97 | 1.94 | 1.69 |
| 4-Hydroxyproline | 1.00 | 1.05 | 0.82 | 0.67 |
| 4-Hydroxyquinoline | 1.00 | 1.66 | 2.16 | 1.67 |
| 4-Imidazoleacetate | 1.00 | 2.58 | 2.99 | 3.41 |
| 4-Methylcatechol | 1.00 | 0.88 | 1.29 | 0.49 |
| 5-Aminosalicylic acid | 1.00 | 87.06 | 105.66 | 78.22 |
| Acetaminophen glucuronide | 1.00 | 0.76 | 2274.00 | 1.64 |
| Acetominophen | 1.00 | 1.44 | 1017.33 | 1.53 |
| Acetylcholine | 1.00 | 1.77 | 1.93 | 1.91 |
| Adenosine | 1.00 | 1.40 | 1.05 | 2.33 |
| Adenosine 3',5'-Diphosphate | 1.00 | 0.00 | 0.00 | 0.00 |
| Adenosine-3-monophosphate | 1.00 | 0.00 | 0.00 | 0.00 |
| Adipic acid | 1.00 | 0.25 | 0.01 | 0.00 |
| Alanine | 1.00 | 0.87 | 1.03 | 0.98 |
| Arabinose | 1.00 | 89.00 | 113.58 | 95.14 |
| Arginine | 1.00 | 3.36 | 5.17 | 4.02 |
| Asp-Phe | 1.00 | 2.31 | 1.98 | 2.49 |
| Benzoic acid | 1.00 | 1.11 | 3.20 | 1.39 |
| Betaine | 1.00 | 0.92 | 0.90 | 1.11 |
| Biliverdin | 1.00 | 270.26 | 301.37 | 575.29 |
| Cadaverine | 1.00 | 1.53 | 1.72 | 1.62 |
| Caffeine | 1.00 | 6.05 | 5.04 | 2.56 |
| Carnitine | 1.00 | 1.24 | 1.85 | 1.54 |
| Cholic acid | 1.00 | 3.49 | 2.52 | 1.23 |
| Choline | 1.00 | 1.58 | 1.59 | 1.64 |
| Citrulline | 1.00 | 1.51 | 1.43 | 1.51 |
| CMPF | 1.00 | 2.31 | 3.14 | 2.03 |
| Cortisol | 1.00 | 1.98 | 3.23 | 2.94 |
| Cortisone | 1.00 | 1.60 | 2.17 | 2.03 |
| Creatine | 1.00 | 0.85 | 0.99 | 0.84 |
| Creatinine | 1.00 | 1.78 | 1.78 | 1.69 |
| Cyclo(Leu-Pro) | 1.00 | 3.71 | 4.21 | 3.31 |
| Cyclohexanamine | 1.00 | 1.79 | 2.19 | 3.30 |
| Cystine | 1.00 | 1.20 | 1.63 | 1.48 |
| Dehydroisoandrosterone sulfate | 1.00 | 0.80 | 0.87 | 0.70 |

| Metabolites | 0 | 90 | MW | END |
|---|---|---|---|---|
| delta-Trimethyllysine | 1.00 | 0.98 | 1.13 | 1.06 |
| Docosahexanoic acid | 1.00 | 0.88 | 1.04 | 0.89 |
| Dopamine | 1.00 | 1.47 | 1.85 | 1.46 |
| Eicosenoic acid | 1.00 | 0.79 | 1.26 | 1.20 |
| Ergothioneine | 1.00 | 0.25 | 0.32 | 0.35 |
| Ethanolamine | 1.00 | 1.56 | 2.71 | 1.81 |
| Ethyl myristate | 1.00 | 0.69 | 0.89 | 0.89 |
| Galactosamine | 1.00 | 0.80 | 0.79 | 0.94 |
| gamma-Glutamylleucine | 1.00 | 3.03 | 3.04 | 2.92 |
| gamma-Glutamyltyrosine | 1.00 | 2.98 | 2.73 | 3.24 |
| Glutamic acid | 1.00 | 0.75 | 0.83 | 0.91 |
| Glutamine | 1.00 | 1.41 | 1.57 | 1.62 |
| Glutarylcarnitine | 1.00 | 0.84 | 1.08 | 0.82 |
| Glu-Thr | 1.00 | 0.15 | 0.20 | 0.17 |
| Glyceraldehyde | 1.00 | 0.42 | 0.29 | 0.24 |
| Glycochenodeoxycholate | 1.00 | 3.64 | 2.58 | 1.71 |
| Glycocholate | 1.00 | 3.92 | 3.86 | 1.09 |
| Guanosine | 1.00 | 12.95 | 1.64 | 14.38 |
| Histamine | 1.00 | 0.90 | 0.91 | 0.88 |
| Histidine | 1.00 | 1.12 | 1.59 | 1.45 |
| Homocysteine | 1.00 | 39.32 | 39.18 | 30.45 |
| Hypoxanthine | 1.00 | 0.14 | 0.08 | 0.20 |
| Indole-3-carboxaldehyde | 1.00 | 1.94 | 2.53 | 2.28 |
| Indolelactic acid | 1.00 | 1.13 | 1.54 | 0.96 |
| Inosine-5-monophosphate | 1.00 | 0.01 | 0.01 | 0.01 |
| Kynurenic acid | 1.00 | 1.57 | 1.99 | 1.59 |
| Kynurenine | 1.00 | 1.66 | 2.34 | 1.67 |
| Lactose | 1.00 | 659.17 | 702.41 | 513.38 |
| Lauroylcarnitine | 1.00 | 2.87 | 4.47 | 3.92 |
| Leucine | 1.00 | 1.35 | 1.41 | 1.31 |
| Linoleic acid | 1.00 | 1.36 | 1.61 | 1.93 |
| Lysine | 1.00 | 1.42 | 1.34 | 1.34 |
| Mannose | 1.00 | 4.02 | 4.36 | 4.05 |
| Methionine | 1.00 | 2.67 | 2.76 | 3.32 |
| Methyl Heptadecanoic acid | 1.00 | 0.67 | 0.81 | 0.81 |
| Methylguanidine | 1.00 | 0.94 | 0.81 | 0.95 |
| Methyltestosterone | 1.00 | 1.33 | 1.52 | 1.77 |
| Methylthioadenosine | 1.00 | 4.14 | 3.92 | 4.29 |
| N,N-Dimethylarginine | 1.00 | 0.16 | 0.20 | 0.27 |
| N2,N2-Dimethylguanosine | 1.00 | 1.76 | 2.36 | 2.01 |
| N-Acetylglucosamine | 1.00 | 159.96 | 158.06 | 154.76 |
| N-Acetylputrescine | 1.00 | 0.80 | 0.77 | 0.64 |
| N-epsilon-Acetyllysine | 1.00 | 2.64 | 2.48 | 2.36 |
| Nicotinamide | 1.00 | 0.79 | 0.61 | 0.30 |
| N-Methylproline | 1.00 | 9.78 | 9.33 | 4.41 |
| Noradrenaline | 1.00 | 1.75 | 1.83 | 2.02 |
| Norleucine | 1.00 | 1.50 | 1.56 | 1.48 |
| Norvaline | 1.00 | 1.80 | 1.77 | 1.74 |
| O-Acetylcarnitine | 1.00 | 1.49 | 1.76 | 1.65 |
| O-Acetylserine | 1.00 | 1.69 | 1.83 | 1.48 |
| Octopine | 1.00 | 2.87 | 2.93 | 2.93 |
| Olmesartan | 1.00 | 1.03 | 0.84 | 0.67 |
| Ornithine | 1.00 | 1.06 | 0.75 | 0.68 |
| Palmitoylcarnitine | 1.00 | 1.57 | 2.81 | 2.57 |
| Pantothenic acid | 1.00 | 2.40 | 3.35 | 2.77 |
| Paraxanthine | 1.00 | 3.40 | 2.99 | 1.95 |
| Phenylacetaldehyde | 1.00 | 1.82 | 1.37 | 1.49 |
| Phenylacetylglutamine | 1.00 | 2.32 | 4.16 | 2.95 |
| Phenylalanine | 1.00 | 1.53 | 1.71 | 1.62 |

| Metabolites | 0 | 90 | MW | END |
|---|---|---|---|---|
| Phenylethanolamine | 1.00 | 2.83 | 3.28 | 3.38 |
| Phosphocholine | 1.00 | 0.73 | 0.83 | 0.87 |
| Phosphoric acid | 1.00 | 2.73 | 2.29 | 2.71 |
| p-Hydroxyphenylacetate | 1.00 | 3.07 | 1.65 | 1.78 |
| Pipecolate | 1.00 | 2.10 | 3.97 | 2.51 |
| Piperine | 1.00 | 2.07 | 6.43 | 3.03 |
| Proline | 1.00 | 2.28 | 1.69 | 1.69 |
| Propanoate | 1.00 | 3.60 | 3.81 | 4.55 |
| Propionylcarnitine | 1.00 | 2.04 | 1.84 | 0.97 |
| Putrescine | 1.00 | 0.96 | 0.87 | 0.94 |
| Pyridoxamine | 1.00 | 1.13 | 1.11 | 1.23 |
| Pyroglutamic acid | 1.00 | 1.42 | 1.49 | 1.60 |
| Quinoline | 1.00 | 2.12 | 4.11 | 2.80 |
| Resorcinol Monoacetate | 1.00 | 1.80 | 2.30 | 2.30 |
| Riboflavin | 1.00 | 2.52 | 2.64 | 1.93 |
| Ribose 5-Phosphate | 1.00 | 14.06 | 13.57 | 15.67 |
| Sedoheptulose | 1.00 | 0.16 | 0.16 | 0.17 |
| Serine | 1.00 | 1.31 | 1.25 | 1.25 |
| Spermine | 1.00 | 0.30 | 0.39 | 0.59 |
| Taurine | 1.00 | 0.67 | 0.67 | 0.91 |
| Theobromine | 1.00 | 4.56 | 6.27 | 2.83 |
| Theophylline | 1.00 | 3.34 | 3.10 | 1.75 |
| Thiazolidine-4-carboxylic acid | 1.00 | 1.01 | 0.88 | 1.02 |
| Threitol | 1.00 | 2.19 | 2.29 | 2.22 |
| Threonic acid | 1.00 | 8.44 | 8.94 | 7.04 |
| Threonine | 1.00 | 1.01 | 0.98 | 1.07 |
| Trigonelline | 1.00 | 3.70 | 2.16 | 3.57 |
| Trimethylamine N-oxide | 1.00 | 2.19 | 2.43 | 3.01 |
| Tryptamine | 1.00 | 1.98 | 2.40 | 2.23 |
| Tryptophan | 1.00 | 1.99 | 2.38 | 2.24 |
| Tyrosine | 1.00 | 1.78 | 1.69 | 1.34 |
| Uracil | 1.00 | 0.56 | 0.76 | 0.55 |
| Uridine | 1.00 | 0.39 | 0.46 | 0.35 |
| Urocanic acid | 1.00 | 3.00 | 2.88 | 3.58 |
| Vanillin | 1.00 | 1.46 | 1.90 | 1.79 |

**Table S5.** VIP metabolites (VIP score >1) alongside both PC1, which describes immediate responses to change in environment, and PC2, which shows the progressive change of the polar metabolome once the subjects are living at the research station. The "x" indicates in which components the metabolites are implied in.

| Metabolites | PC1 | PC2 | Metabolites | PC1 | PC2 |
|---|---|---|---|---|---|
| 1,3 Cyclohexanedione | x | x | Lauroylcarnitine | x | x |
| 1,7-Dimethyluric acid | | x | Leucine | | x |
| 1-Methyladenosine | x | x | Mannose | x | x |
| 1-Methylnicotinamide | x | x | Methionine | x | x |
| 2'-Deoxyguanosine 5'-monophosphate | x | x | Methylthioadenosine | x | x |
| 2-Phenylacetamide | | x | N,N-Dimethylarginine | x | |
| 3-(1-Pyrazolyl)-alanine | x | x | N2,N2-Dimethylguanosine | x | x |
| 3-Indoleacetic acid | x | | N-Acetylglucosamine | x | x |
| 3-Methoxytyrosine | x | | N-Acetylputrescine | x | x |
| 4-Guanidinobutyric acid | | x | N-epsilon-Acetyllysine | x | x |
| 4-Hydroxy-L-Phenylglycine | | x | Nicotinamide | x | x |
| 4-Imidazoleacetate | x | x | N-Methylproline | | x |
| 5-Aminosalicylic acid | x | x | Noradrenaline | x | x |
| Acetylcarnitine | x | x | Norleucine | x | x |
| Acetylcholine | x | | Norvaline | x | x |
| Adenosine 3',5'-Diphosphate | x | x | O-Acetylcarnitine | x | x |
| Adenosine-3-monophosphate | x | x | Octopine | x | x |
| Adipic acid | x | x | Oxoproline | | x |
| Arabinose | x | x | Palmitoylcarnitine | x | x |
| Arginine | x | x | Phenylethanolamine | x | x |
| Asp-Phe | x | x | Phosphocholine | | x |
| Biliverdin | x | x | Phosphoric acid | x | x |
| Cadaverine | x | x | Proline | | x |
| Caffeine | | x | Propanoate | x | x |
| Carnitine | x | | Propionylcarnitine | | x |
| Choline | x | x | Pyroglutamic acid | x | x |
| Cortisol | x | x | Quinoline | x | |
| Cortisone | x | x | Resorcinol Monoacetate | x | x |
| Creatinine | x | x | Riboflavin | | x |
| Cyclohexanamine | x | x | Sedoheptulose | x | x |
| Cystine | x | x | Theobromine | | x |
| D-Alanine | x | | Threitol | x | x |
| D-Ornithine | x | x | Threonic acid | x | x |
| D-Ribose 5-Phosphate | x | x | Trans-4-Hydroxy-L-Proline | | x |
| gamma-Glutamylleucine | x | x | Tryptamine | x | x |
| gamma-Glutamyltyrosine | x | x | Tryptophan | x | x |
| Glutamine | x | x | Tyrosine | | x |
| Glu-Thr | x | x | Uridine | x | x |
| Glyceraldehyde | x | x | Urocanic acid | x | x |
| Glycocholate | | x | Vanillin | x | x |
| Guanosine | x | | | | |
| Homocysteine | x | x | | | |
| Indole-3-carboxaldehyde | x | x | | | |
| Inosine-5-monophosphate | x | x | | | |
| Lactose | x | x | | | |

**Figure S1.** Heatmap of all lipids identified in the subjects alongside time (0, 90, MW, END), after transformation to z-values.

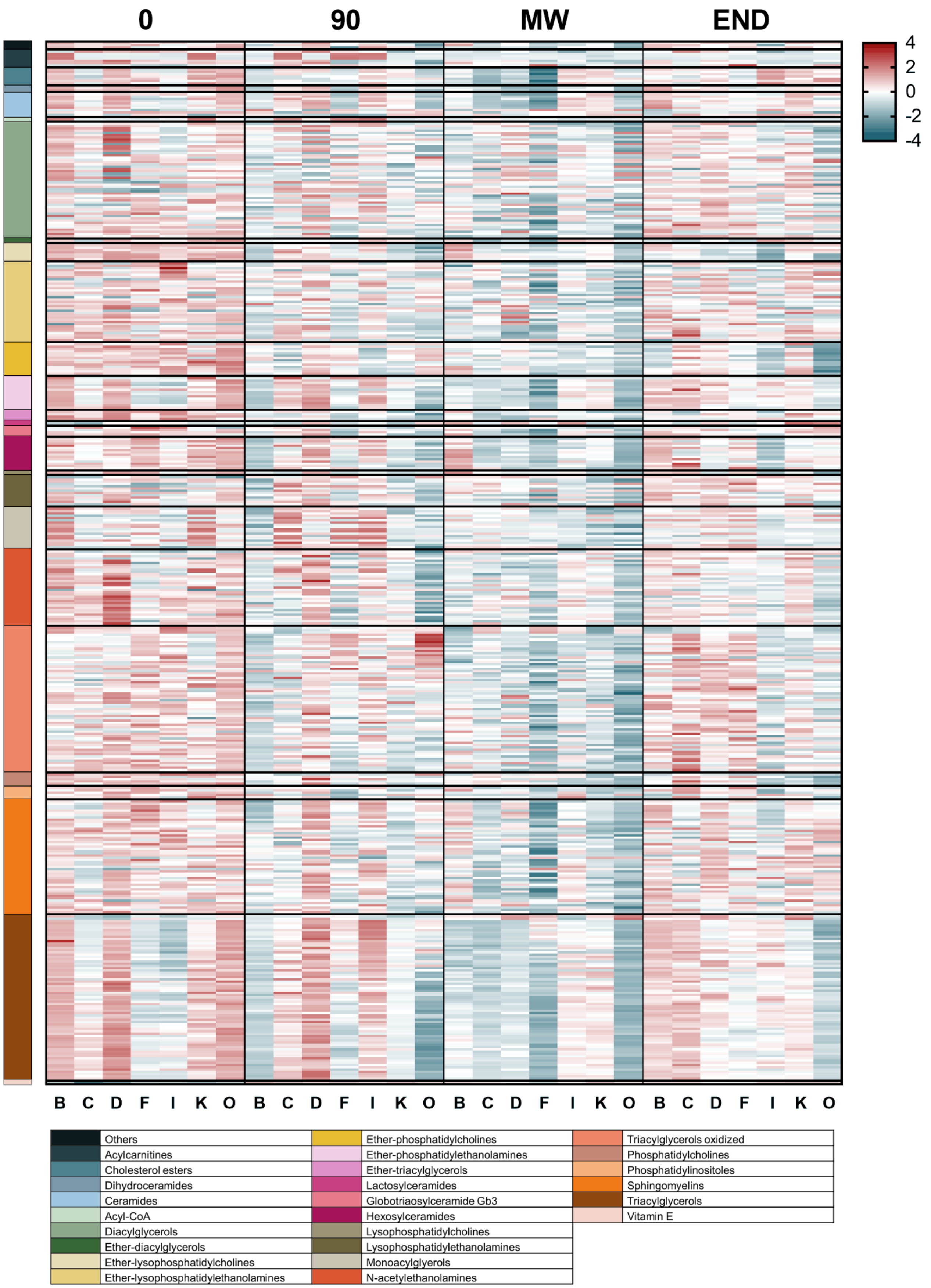

**Figure S2.** Boxplots figuring the fluctuations of the proteogenic aminoacidic pool, expressed as sum of each mass intensity, over time-points in all subjects.

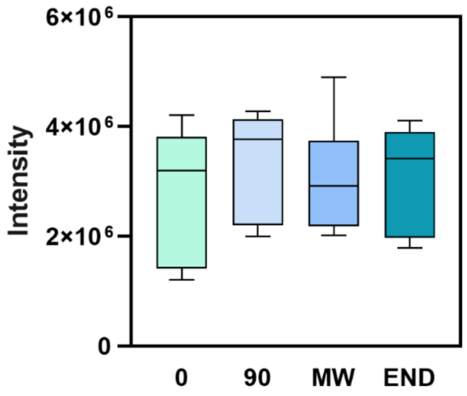

**Figure S3.** Fluctuations of the amino acids, expressed as mass intensities, involved in modulated pathways for each subject.

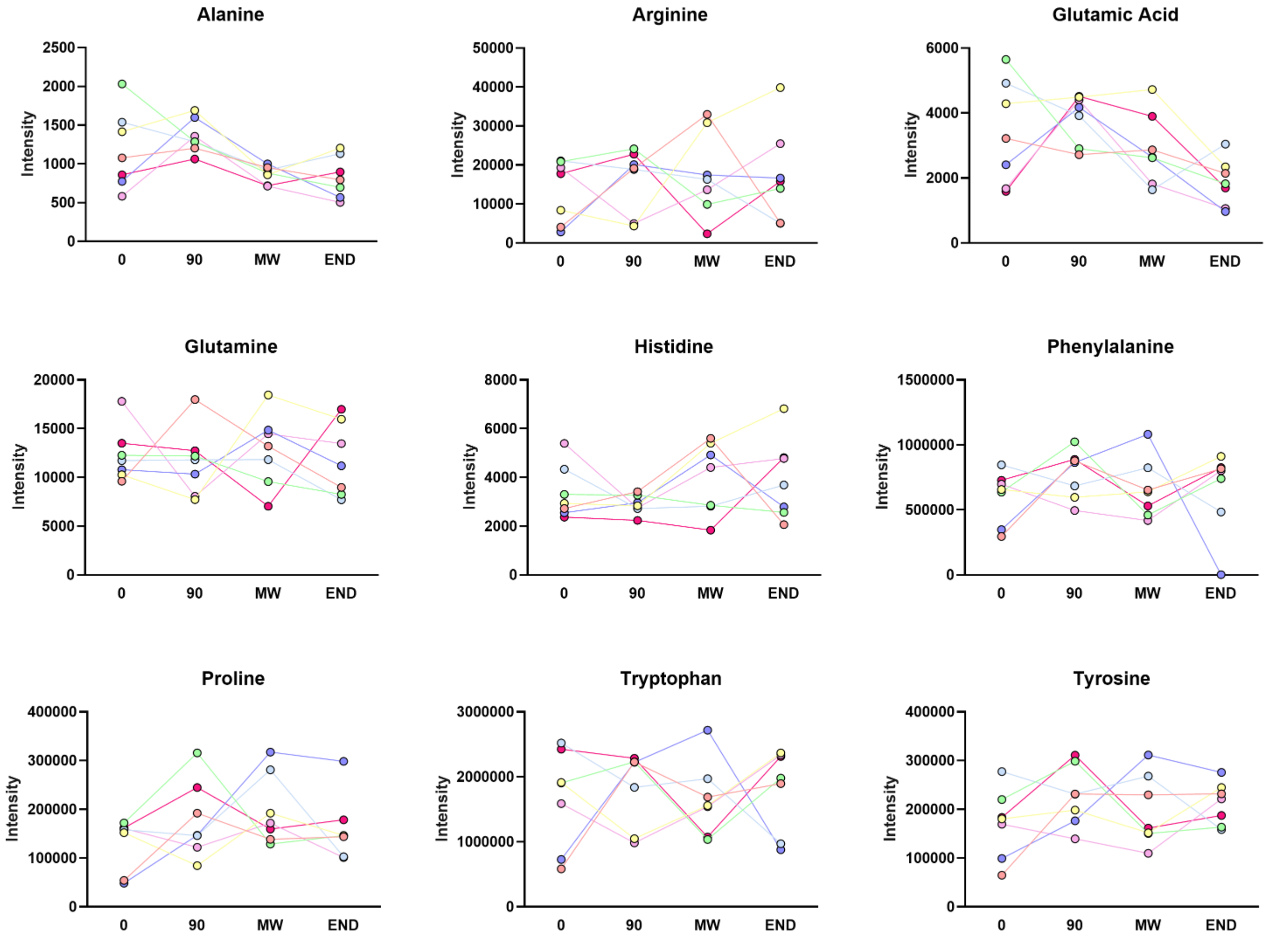

**Figure S4.** Fluctuations of metabolites central to nitrogen metabolism, expressed as mass intensities, involved in modulated pathways for each subject.

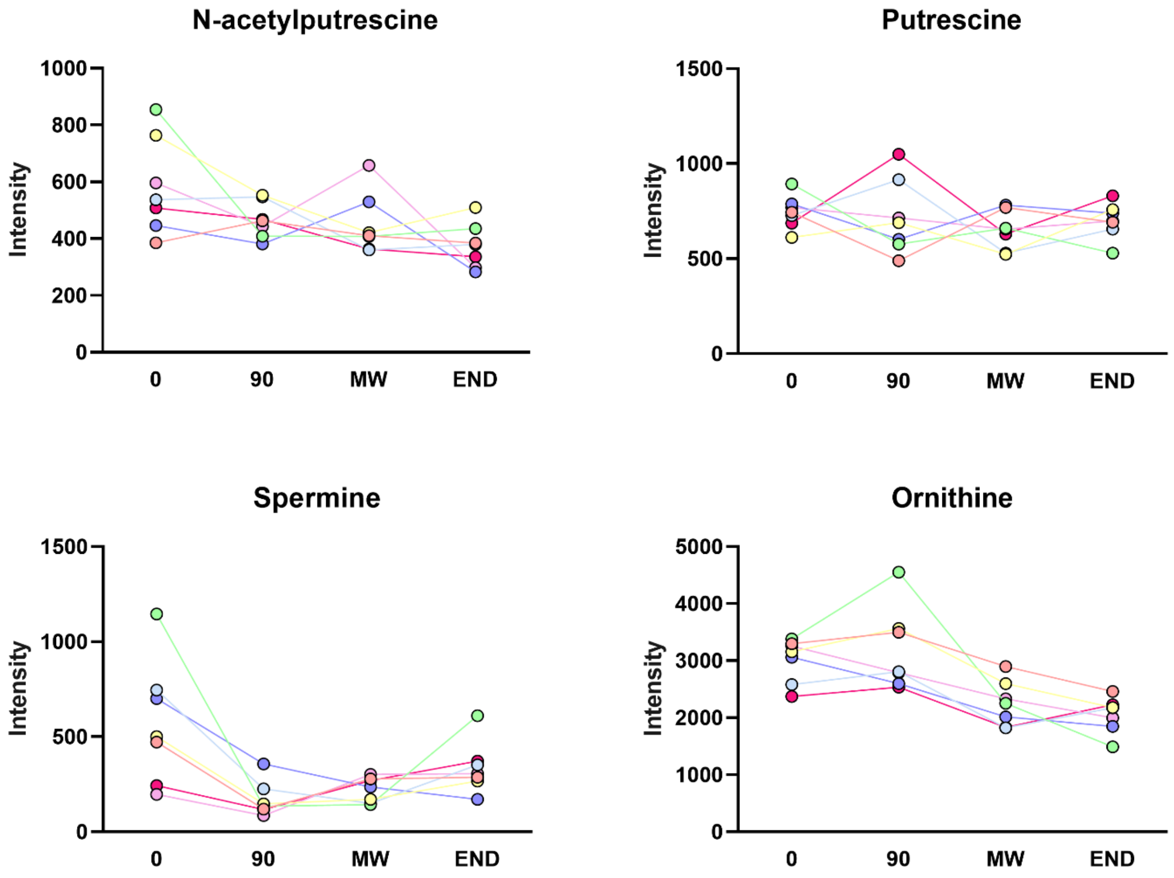

# Raw data table



| Subject | B | B | B | B |
|---|---|---|---|---|
| Time Point | 0 | 90 | MW | END |
| **metabolites** | Sample 1 | Sample 2 | Sample 3 | Sample 4 |
| 1,3 Cyclohexanedione | 612.74 | 1826.28 | 2507.78 | 3063.10 |
| 1,7-Dimethyluric acid | 187.11 | 2854.14 | 4363.10 | 6917.18 |
| 1-Methyladenosine | 1410.19 | 4737.76 | 6012.82 | 6662.20 |
| 1-Methylhistidine | 3392.13 | 2733.34 | 3606.35 | 3028.44 |
| 1-Methylnicotinamide | 291.89 | 679.31 | 1520.49 | 757.37 |
| 2'-Deoxyguanosine 5'-monophosphate | 5788.85 | 108.26 | 95.74 | 101.70 |
| 2-Hydroxy-4-methylpentanoic acid | 6126.27 | 3767.83 | 5492.20 | 5439.46 |
| 2-Phenylacetamide | 26147.99 | 63292.98 | 69501.40 | 104161.85 |
| 3-(1-Pyrazolyl)-alanine | 120.61 | 454.54 | 549.90 | 539.46 |
| 3-(2-Hydroxyphenyl)propanoic acid | 5335.91 | 8001.49 | 5842.85 | 12017.98 |
| 3-Hydroxybenzaldehyde | 3892.08 | 13659.37 | 15095.07 | 22288.64 |
| 3-Indoleacetic acid | 6366.34 | 18459.63 | 46262.07 | 28412.22 |
| 3-Indolepropionic acid | 2153.21 | 2300.31 | 11069.35 | 2927.57 |
| 3-Methoxytyrosine | 344.31 | 860.55 | 817.07 | 1114.34 |
| 4-Aminophenol | 34.39 | 40.72 | 37.89 | 53.09 |
| 4-Guanidinobutanoate | 60.05 | 158.37 | 295.75 | 146.84 |
| 4-Hydroxyhippuric acid | 25.40 | 461.62 | 97.82 | 373.41 |
| 4-Hydroxyphenylglycine | 2046.96 | 3264.02 | 3696.31 | 3754.18 |
| 4-Hydroxyproline | 1061.60 | 1043.06 | 1120.44 | 708.09 |
| 4-Hydroxyquinoline | 441.66 | 1205.00 | 1418.33 | 1891.10 |
| 4-Imidazoleacetate | 1917.68 | 3738.01 | 5325.82 | 4914.23 |
| 4-Methylcatechol | 416.12 | 27.45 | 866.92 | 90.71 |
| 5-Aminosalicylic acid | 4.90 | 2656.41 | 4406.16 | 3074.86 |
| Acetaminophen glucuronide | 0.00 | 0.00 | 0.00 | 6.24 |
| Acetominophen | 0.00 | 5.27 | 5.66 | 3.97 |
| Acetylcholine | 9779.95 | 17568.40 | 21452.90 | 19294.01 |
| Adenosine | 366.08 | 355.58 | 322.43 | 856.57 |
| Adenosine 3',5'-Diphosphate | 919.08 | 29.63 | 16.96 | 17.63 |
| Adenosine-3-monophosphate | 8772.40 | 13.97 | 25.79 | 10.18 |
| Adipic acid | 11581.42 | 38.96 | 45.10 | 43.39 |
| Alanine | 1078.31 | 1414.79 | 2033.23 | 1538.71 |
| Arabinose | 9.76 | 1332.19 | 1930.02 | 2119.57 |
| Arginine | 4052.76 | 8391.74 | 20866.69 | 21043.50 |
| Asp-Phe | 128.82 | 348.98 | 293.98 | 379.76 |
| Benzoic acid | 450.55 | 828.53 | 371.74 | 2639.85 |
| Betaine | 59382.16 | 52321.44 | 56971.55 | 65580.38 |
| Biliverdin | 5.29 | 587.14 | 594.44 | 1086.77 |
| Cadaverine | 585.64 | 1016.47 | 1389.16 | 948.14 |
| Caffeine | 6003.90 | 101171.13 | 284126.85 | 427874.80 |
| Carnitine | 3778.35 | 3114.61 | 5230.47 | 6426.72 |
| Cholic acid | 81928.99 | 4860.15 | 101953.11 | 32644.05 |
| Choline | 163826.15 | 203858.25 | 221488.70 | 235512.35 |
| Citrulline | 2708.77 | 3699.96 | 4621.91 | 4270.00 |
| CMPF | 106772.05 | 513986.35 | 484160.75 | 396368.15 |
| Cortisol | 8615.34 | 55271.81 | 43731.70 | 78275.56 |
| Cortisone | 1452.18 | 4038.02 | 6031.49 | 6165.03 |
| Creatine | 61079.54 | 93009.29 | 117847.46 | 87100.26 |

| Subject | B | B | B | B |
|---|---|---|---|---|
| Time Point | 0 | 90 | MW | END |
| **metabolites** | Sample 1 | Sample 2 | Sample 3 | Sample 4 |
| Creatinine | 31974.36 | 60428.62 | 77853.05 | 70959.84 |
| Cyclo(Leu-Pro) | 2507.86 | 10599.66 | 14604.28 | 15024.42 |
| Cyclohexanamine | 374.56 | 866.36 | 866.56 | 960.32 |
| Cystine | 817.34 | 822.56 | 1392.21 | 1206.92 |
| Dehydroisoandrosterone sulfate | 675547.95 | 550490.60 | 549918.95 | 527185.50 |
| delta-Trimethyllysine | 740.77 | 848.76 | 1156.88 | 698.97 |
| Docosahexanoic acid | 65748.01 | 59045.96 | 138551.55 | 48725.12 |
| Dopamine | 230.96 | 477.89 | 558.97 | 662.69 |
| Eicosenoic acid | 3338.00 | 2853.38 | 5841.36 | 1247.07 |
| Ergothioneine | 4836.68 | 1003.56 | 1212.14 | 1940.82 |
| Ethanolamine | 857.77 | 1544.72 | 2492.82 | 1664.62 |
| Ethyl myristate | 399686.30 | 179993.50 | 426094.05 | 178990.00 |
| Galactosamine | 272.43 | 252.34 | 308.67 | 400.91 |
| gamma-Glutamylleucine | 10257.81 | 33352.62 | 38998.37 | 47088.54 |
| gamma-Glutamyltyrosine | 1707.06 | 5012.15 | 6455.53 | 8887.24 |
| Glutamic acid | 3216.32 | 4287.66 | 5645.38 | 4915.03 |
| Glutamine | 9603.90 | 10270.94 | 12280.38 | 11719.51 |
| Glutarylcarnitine | 923.55 | 1774.39 | 1858.99 | 2380.65 |
| Glu-Thr | 3389.47 | 538.93 | 363.55 | 665.05 |
| Glyceraldehyde | 194294.35 | 99030.70 | 47810.07 | 62060.29 |
| Glycochenodeoxycholate | 51082.18 | 231897.75 | 327944.85 | 64755.42 |
| Glycocholate | 10048.38 | 104205.75 | 183138.65 | 8667.36 |
| Guanosine | 10.24 | 195.59 | 30.38 | 160.46 |
| Histamine | 430.87 | 376.50 | 560.29 | 474.89 |
| Histidine | 2724.20 | 2934.97 | 3303.29 | 4333.68 |
| Homocysteine | 303.65 | 11651.60 | 18673.63 | 15431.65 |
| Hypoxanthine | 24222.96 | 35980.21 | 23698.33 | 49791.10 |
| Indole-3-carboxaldehyde | 16031.83 | 49307.21 | 52413.44 | 68698.25 |
| Indolelactic acid | 19249.16 | 24454.54 | 23034.37 | 24117.59 |
| Inosine-5-monophosphate | 4643.89 | 18.06 | 29.16 | 54.85 |
| Kynurenic acid | 565.55 | 968.33 | 1215.97 | 1289.37 |
| Kynurenine | 5254.69 | 15795.05 | 18822.89 | 25622.51 |
| Lactose | 0.00 | 1514.91 | 1633.04 | 1730.84 |
| Lauroylcarnitine | 1523.24 | 4559.69 | 6449.16 | 4752.41 |
| Leucine | 174521.85 | 237551.15 | 286151.15 | 307290.40 |
| Linoleic acid | 149798.90 | 169588.90 | 410994.05 | 186671.85 |
| Lysine | 4331.02 | 4567.54 | 5426.83 | 5171.02 |
| Mannose | 130141.10 | 418478.85 | 540684.15 | 526687.60 |
| Methionine | 7912.58 | 23590.79 | 26229.75 | 45004.98 |
| Methyl Heptadecanoic acid | 148044.55 | 94395.03 | 163383.20 | 81755.75 |
| Methylguanidine | 688.85 | 638.15 | 594.89 | 630.23 |
| Methyltestosterone | 12867.27 | 11898.58 | 22642.72 | 11589.99 |
| Methylthioadenosine | 216.36 | 1131.36 | 1053.27 | 1149.34 |
| N,N-Dimethylarginine | 11399.01 | 1385.15 | 1346.88 | 1445.18 |
| N2,N2-Dimethylguanosine | 444.43 | 1371.84 | 1640.48 | 1694.77 |
| N-Acetylglucosamine | 7.62 | 583.98 | 795.13 | 858.77 |
| N-Acetylputrescine | 385.24 | 462.87 | 410.53 | 384.53 |

| Subject | B | B | B | B |
|---|---|---|---|---|
| **Time Point** | **0** | **90** | **MW** | **END** |
| **metabolites** | Sample 1 | Sample 2 | Sample 3 | Sample 4 |
| N-epsilon-Acetyllysine | 617.25 | 1859.98 | 2347.52 | 2319.88 |
| Nicotinamide | 1193.28 | 3273.24 | 3873.71 | 1997.71 |
| N-Methylproline | 92.47 | 6682.76 | 13503.95 | 2233.95 |
| Noradrenaline | 958.67 | 1507.91 | 1384.24 | 1318.36 |
| Norleucine | 88428.41 | 143620.10 | 188046.75 | 197842.95 |
| Norvaline | 63062.98 | 132065.60 | 172737.50 | 152875.95 |
| O-Acetylcarnitine | 70440.02 | 192308.25 | 235696.95 | 229301.75 |
| O-Acetylserine | 5735.62 | 24679.95 | 32125.07 | 36477.03 |
| Octopine | 84.72 | 763.59 | 950.95 | 1441.54 |
| Olmesartan | 30.76 | 50.74 | 64.09 | 28.83 |
| Ornithine | 3298.08 | 3498.42 | 2898.03 | 2460.54 |
| Palmitoylcarnitine | 2391.34 | 6525.45 | 13554.43 | 17065.99 |
| Pantothenic acid | 612.83 | 3521.12 | 2551.94 | 3828.31 |
| Paraxanthine | 14401.89 | 190256.45 | 301001.60 | 418660.05 |
| Phenylacetaldehyde | 1083.45 | 1349.46 | 467.98 | 2093.37 |
| Phenylacetylglutamine | 17249.29 | 11411.62 | 123334.38 | 24476.03 |
| Phenylalanine | 295581.90 | 657061.95 | 637386.50 | 846808.20 |
| Phenylethanolamine | 72.53 | 291.49 | 344.89 | 531.45 |
| Phosphocholine | 79825.41 | 89645.51 | 56689.07 | 79553.91 |
| Phosphoric acid | 13127.87 | 36864.21 | 46412.63 | 46678.92 |
| P-Hydroxyphenylacetate | 334.60 | 377.77 | 691.66 | 398.50 |
| Pipecolate | 5389.38 | 12080.31 | 23624.29 | 11620.11 |
| Piperine | 357.09 | 103.22 | 11835.80 | 244.13 |
| Proline | 54122.78 | 152044.65 | 172089.70 | 158248.60 |
| Propanoate | 4402.47 | 13041.71 | 15528.22 | 14138.58 |
| Propionylcarnitine | 5744.60 | 25108.43 | 23966.95 | 21880.95 |
| Putrescine | 744.89 | 489.17 | 769.85 | 691.31 |
| Pyridoxamine | 2329.58 | 2510.38 | 2198.47 | 2684.96 |
| Pyroglutamic acid | 17416.00 | 19647.60 | 22343.51 | 22519.86 |
| Quinoline | 4467.97 | 13676.42 | 36020.50 | 20942.03 |
| Resorcinol Monoacetate | 1806.20 | 4995.87 | 5528.18 | 7127.77 |
| Riboflavin | 137.73 | 442.11 | 372.16 | 453.57 |
| Ribose 5-Phosphate | 318.08 | 3812.66 | 5043.36 | 6104.61 |
| Sedoheptulose | 993.31 | 106.26 | 168.96 | 177.61 |
| Serine | 573.22 | 635.80 | 1060.79 | 719.95 |
| Spermine | 471.62 | 119.33 | 277.96 | 287.14 |
| Taurine | 4464.00 | 3390.40 | 1938.94 | 4287.68 |
| Theobromine | 16631.41 | 78318.56 | 127115.34 | 75583.87 |
| Theophylline | 1781.76 | 27097.59 | 45815.34 | 57057.45 |
| Thiazolidine-4-carboxylic acid | 2037.34 | 2510.62 | 4299.60 | 3766.96 |
| Threitol | 1240.55 | 2145.01 | 2895.48 | 2029.36 |
| Threonic acid | 2231.47 | 24851.24 | 18410.46 | 19178.49 |
| Threonine | 1143.59 | 1316.11 | 1397.34 | 1576.97 |
| Trigonelline | 6627.46 | 16558.04 | 14458.16 | 25629.84 |
| Trimethylamine N-oxide | 1751.87 | 3881.28 | 5607.74 | 2531.54 |
| Tryptamine | 9629.50 | 31719.31 | 31189.00 | 41757.48 |
| Tryptophan | 583178.25 | 1912672.00 | 1907466.00 | 2522181.00 |

| Subject | B | B | B | B |
|---|---|---|---|---|
| **Time Point** | **0** | **90** | **MW** | **END** |
| **metabolites** | Sample 1 | Sample 2 | Sample 3 | Sample 4 |
| Tyrosine | 64943.82 | 180396.30 | 220226.30 | 277594.75 |
| Uracil | 1754.62 | 3604.55 | 4592.15 | 5525.73 |
| Uridine | 20150.17 | 16315.55 | 18369.74 | 21391.87 |
| Urocanic acid | 565.87 | 1529.99 | 1128.53 | 2099.39 |
| Vanillin | 1566.28 | 3352.17 | 4300.15 | 5142.78 |

| Subject | C | C | C | C |
|---|---|---|---|---|
| **Time Point** | **0** | **90** | **MW** | **END** |
| **metabolites** | Sample 5 | Sample 6 | Sample 7 | Sample 8 |
| 1,3 Cyclohexanedione | 1044.29 | 1653.32 | 3398.01 | 2721.50 |
| 1,7-Dimethyluric acid | 87.02 | 2227.27 | 811.70 | 623.07 |
| 1-Methyladenosine | 1844.94 | 5323.97 | 11083.35 | 8395.57 |
| 1-Methylhistidine | 3051.46 | 3420.54 | 4705.07 | 3834.88 |
| 1-Methylnicotinamide | 104.89 | 1467.24 | 2221.58 | 1984.89 |
| 2'-Deoxyguanosine 5'-monophosphate | 6003.01 | 109.64 | 79.83 | 109.13 |
| 2-Hydroxy-4-methylpentanoic acid | 8926.18 | 4661.17 | 16402.05 | 5136.55 |
| 2-Phenylacetamide | 41407.20 | 62678.99 | 60219.11 | 87415.15 |
| 3-(1-Pyrazolyl)-alanine | 43.71 | 631.79 | 342.66 | 588.55 |
| 3-(2-Hydroxyphenyl)propanoic acid | 5679.24 | 10341.80 | 9617.90 | 5600.12 |
| 3-Hydroxybenzaldehyde | 7755.65 | 13544.54 | 12321.33 | 18569.67 |
| 3-Indoleacetic acid | 6913.16 | 10199.34 | 32191.42 | 21433.82 |
| 3-Indolepropionic acid | 7407.75 | 6520.62 | 25708.58 | 21668.06 |
| 3-Methoxytyrosine | 281.55 | 598.27 | 771.70 | 871.25 |
| 4-Aminophenol | 35.75 | 47.64 | 69.32 | 33.56 |
| 4-Guanidinobutanoate | 58.28 | 328.66 | 668.56 | 560.48 |
| 4-Hydroxyhippuric acid | 35.96 | 22.00 | 71.64 | 145.54 |
| 4-Hydroxyphenylglycine | 2264.10 | 6279.90 | 5220.73 | 5779.82 |
| 4-Hydroxyproline | 1005.47 | 1284.68 | 1293.08 | 1037.01 |
| 4-Hydroxyquinoline | 543.66 | 1058.55 | 1775.25 | 1321.59 |
| 4-Imidazoleacetate | 1460.71 | 4392.15 | 4195.86 | 6336.13 |
| 4-Methylcatechol | 243.76 | 258.39 | 1225.35 | 460.43 |
| 5-Aminosalicylic acid | 20.63 | 1380.17 | 1931.03 | 1713.24 |
| Acetaminophen glucuronide | 3.93 | 5.86 | 0.00 | 0.00 |
| Acetominophen | 6.48 | 2.64 | 2.86 | 8.69 |
| Acetylcholine | 12799.91 | 32713.30 | 34161.81 | 46076.68 |
| Adenosine | 299.60 | 500.60 | 389.47 | 2405.39 |
| Adenosine 3',5'-Diphosphate | 1842.63 | 16.19 | 16.62 | 10.66 |
| Adenosine-3-monophosphate | 5501.40 | 27.72 | 0.00 | 11.68 |
| Adipic acid | 10193.18 | 67.72 | 45.73 | 49.48 |
| Alanine | 774.22 | 581.31 | 858.98 | 1203.81 |
| Arabinose | 6.39 | 1837.84 | 2462.85 | 2951.79 |
| Arginine | 2799.17 | 19305.87 | 17765.31 | 19152.61 |
| Asp-Phe | 115.58 | 499.39 | 379.02 | 417.05 |
| Benzoic acid | 151.47 | 921.57 | 123.31 | 750.93 |
| Betaine | 68533.85 | 69737.98 | 53166.44 | 106986.35 |
| Biliverdin | 7.89 | 1065.79 | 9.75 | 3986.27 |
| Cadaverine | 566.10 | 1032.22 | 820.97 | 920.66 |
| Caffeine | 1295.32 | 53066.38 | 20094.12 | 7530.58 |
| Carnitine | 2713.58 | 4087.00 | 7036.89 | 11198.81 |
| Cholic acid | 721.11 | 33127.27 | 183469.65 | 621.22 |
| Choline | 146875.00 | 234724.70 | 201677.45 | 256492.40 |
| Citrulline | 3760.91 | 5847.76 | 5662.04 | 7778.58 |
| CMPF | 135527.50 | 163969.10 | 316247.45 | 151615.45 |
| Cortisol | 12049.68 | 32810.27 | 57750.82 | 50415.23 |
| Cortisone | 1631.59 | 3791.85 | 5733.98 | 5323.79 |
| Creatine | 37024.15 | 36753.78 | 47916.97 | 56068.84 |

| Subject | C | C | C | C |
| --- | --- | --- | --- | --- |
| Time Point | 0 | 90 | MW | END |
| **metabolites** | Sample 5 | Sample 6 | Sample 7 | Sample 8 |
| Creatinine | 26437.75 | 82278.97 | 80849.17 | 94565.74 |
| Cyclo(Leu-Pro) | 2449.18 | 17996.07 | 27253.63 | 19631.36 |
| Cyclohexanamine | 289.40 | 521.75 | 717.50 | 1655.82 |
| Cystine | 681.50 | 1043.44 | 780.41 | 989.82 |
| Dehydroisoandrosterone sulfate | 2347337.00 | 1988112.00 | 2543892.50 | 1914752.00 |
| delta-Trimethyllysine | 691.69 | 1060.29 | 887.29 | 843.92 |
| Docosahexanoic acid | 112861.09 | 154909.80 | 156115.15 | 60066.30 |
| Dopamine | 211.32 | 514.01 | 742.23 | 641.32 |
| Eicosenoic acid | 4283.19 | 7788.29 | 5341.43 | 4776.66 |
| Ergothioneine | 3570.66 | 1296.96 | 2060.28 | 3183.01 |
| Ethanolamine | 1062.62 | 3513.96 | 10572.62 | 6616.71 |
| Ethyl myristate | 473307.45 | 540113.15 | 280359.65 | 457181.20 |
| Galactosamine | 267.75 | 276.92 | 276.80 | 411.16 |
| gamma-Glutamylleucine | 10384.89 | 29169.94 | 35216.61 | 44854.23 |
| gamma-Glutamyltyrosine | 1441.49 | 4572.70 | 4528.58 | 7105.83 |
| Glutamic acid | 2408.05 | 1674.34 | 1587.96 | 2716.87 |
| Glutamine | 10794.77 | 17815.65 | 13500.90 | 17997.35 |
| Glutarylcarnitine | 1829.92 | 1632.71 | 1832.16 | 2234.73 |
| Glu-Thr | 2944.94 | 532.23 | 526.95 | 823.77 |
| Glyceraldehyde | 199284.35 | 92304.56 | 68601.66 | 38506.12 |
| Glycochenodeoxycholate | 131338.34 | 315021.40 | 94067.39 | 202149.75 |
| Glycocholate | 37730.84 | 93793.57 | 29825.92 | 23535.48 |
| Guanosine | 19.81 | 505.55 | 23.51 | 467.43 |
| Histamine | 506.16 | 764.23 | 355.27 | 529.68 |
| Histidine | 2548.83 | 5393.80 | 2371.52 | 3415.21 |
| Homocysteine | 106.11 | 14588.98 | 16703.60 | 16385.37 |
| Hypoxanthine | 78876.67 | 52986.01 | 30673.45 | 54823.56 |
| Indole-3-carboxaldehyde | 20010.92 | 41594.22 | 67142.43 | 61266.33 |
| Indolelactic acid | 21933.01 | 20024.51 | 49696.62 | 16537.98 |
| Inosine-5-monophosphate | 4274.99 | 44.94 | 47.03 | 39.83 |
| Kynurenic acid | 339.61 | 750.59 | 884.19 | 1106.98 |
| Kynurenine | 5398.51 | 14128.23 | 25982.39 | 22274.81 |
| Lactose | 0.00 | 1926.66 | 14784.70 | 2081.98 |
| Lauroylcarnitine | 1677.99 | 16635.05 | 9455.04 | 13929.17 |
| Leucine | 161385.75 | 210983.85 | 246913.80 | 308206.35 |
| Linoleic acid | 159380.55 | 497688.15 | 135313.00 | 422911.75 |
| Lysine | 3203.84 | 5207.85 | 3241.00 | 5144.96 |
| Mannose | 94747.14 | 456852.25 | 563711.05 | 627805.55 |
| Methionine | 9825.78 | 30540.17 | 25251.49 | 44617.29 |
| Methyl Heptadecanoic acid | 170513.82 | 173864.60 | 133570.85 | 123294.20 |
| Methylguanidine | 911.45 | 664.64 | 619.97 | 798.20 |
| Methyltestosterone | 16335.72 | 41710.37 | 9832.42 | 39376.79 |
| Methylthioadenosine | 225.77 | 1257.62 | 1228.66 | 1361.83 |
| N,N-Dimethylarginine | 13351.76 | 2049.31 | 2008.44 | 4958.40 |
| N2,N2-Dimethylguanosine | 487.80 | 1154.74 | 2184.87 | 1659.76 |
| N-Acetylglucosamine | 1.28 | 882.55 | 925.87 | 1134.58 |
| N-Acetylputrescine | 763.45 | 552.73 | 421.51 | 509.24 |

| Subject | C | C | C | C |
|---|---|---|---|---|
| **Time Point** | **0** | **90** | **MW** | **END** |
| **metabolites** | Sample 5 | Sample 6 | Sample 7 | Sample 8 |
| N-epsilon-Acetyllysine | 327.23 | 1622.14 | 2197.41 | 2005.19 |
| Nicotinamide | 2865.13 | 2723.41 | 3275.57 | 2082.01 |
| N-Methylproline | 449.94 | 5979.64 | 3334.64 | 757.11 |
| Noradrenaline | 663.61 | 1090.12 | 1329.20 | 2053.63 |
| Norleucine | 90190.14 | 118632.05 | 180638.25 | 200293.40 |
| Norvaline | 53010.90 | 117831.02 | 118683.24 | 161630.40 |
| O-Acetylcarnitine | 71864.21 | 194444.50 | 210898.75 | 241534.70 |
| O-Acetylserine | 15350.71 | 41489.94 | 51312.96 | 43319.79 |
| Octopine | 130.29 | 706.42 | 765.48 | 1118.42 |
| Olmesartan | 45.90 | 32.71 | 39.89 | 14.90 |
| Ornithine | 3157.74 | 3565.00 | 2597.76 | 2177.42 |
| Palmitoylcarnitine | 1812.52 | 7497.87 | 10495.11 | 11849.48 |
| Pantothenic acid | 846.20 | 1394.31 | 1860.26 | 2545.33 |
| Paraxanthine | 2136.78 | 108992.70 | 28379.25 | 24435.05 |
| Phenylacetaldehyde | 1692.98 | 2940.17 | 3148.12 | 3432.74 |
| Phenylacetylglutamine | 9269.68 | 28449.29 | 67579.06 | 23627.79 |
| Phenylalanine | 347593.45 | 698257.60 | 728261.30 | 878239.80 |
| Phenylethanolamine | 92.12 | 399.73 | 506.82 | 456.86 |
| Phosphocholine | 82340.22 | 54226.54 | 66956.98 | 62182.17 |
| Phosphoric acid | 18318.61 | 40381.16 | 31329.57 | 41170.76 |
| P-Hydroxyphenylacetate | 312.05 | 734.13 | 467.78 | 295.89 |
| Pipecolate | 5117.90 | 28330.44 | 95711.64 | 64785.44 |
| Piperine | 265.50 | 326.46 | 30832.15 | 7714.37 |
| Proline | 48619.20 | 160873.10 | 161837.25 | 192133.95 |
| Propanoate | 3698.46 | 14163.23 | 11790.12 | 18963.88 |
| Propionylcarnitine | 4141.38 | 14285.55 | 11120.18 | 12138.60 |
| Putrescine | 612.02 | 689.13 | 523.43 | 757.89 |
| Pyridoxamine | 1665.16 | 2227.84 | 1825.09 | 2959.94 |
| Pyroglutamic acid | 18055.33 | 32924.06 | 23068.57 | 31142.89 |
| Quinoline | 5013.47 | 7544.20 | 24283.86 | 15143.67 |
| Resorcinol Monoacetate | 2536.76 | 5005.35 | 5602.57 | 7479.02 |
| Riboflavin | 139.51 | 497.11 | 405.89 | 400.37 |
| Ribose 5-Phosphate | 365.53 | 4161.89 | 4340.71 | 3612.70 |
| Sedoheptulose | 1222.71 | 140.74 | 126.83 | 145.30 |
| Serine | 561.67 | 930.44 | 615.99 | 808.58 |
| Spermine | 500.84 | 147.35 | 170.88 | 267.31 |
| Taurine | 4501.90 | 4221.79 | 2579.01 | 4972.64 |
| Theobromine | 5916.48 | 82920.23 | 229986.55 | 147595.50 |
| Theophylline | 736.05 | 23052.64 | 5881.79 | 5054.65 |
| Thiazolidine-4-carboxylic acid | 3732.32 | 5939.27 | 4008.03 | 7276.73 |
| Threitol | 832.20 | 2234.25 | 2099.49 | 2521.91 |
| Threonic acid | 2510.47 | 22498.44 | 25107.11 | 14079.02 |
| Threonine | 1271.89 | 1529.01 | 1315.03 | 1588.80 |
| Trigonelline | 5387.25 | 18287.33 | 13999.89 | 21648.68 |
| Trimethylamine N-oxide | 751.14 | 2509.21 | 1579.97 | 7247.45 |
| Tryptamine | 12463.76 | 26324.67 | 41094.99 | 36589.90 |
| Tryptophan | 727529.70 | 1589631.00 | 2426292.00 | 2228497.50 |

| Subject | C | C | C | C |
|---|---|---|---|---|
| **Time Point** | **0** | **90** | **MW** | **END** |
| **metabolites** | Sample 5 | Sample 6 | Sample 7 | Sample 8 |
| Tyrosine | 99280.08 | 169445.20 | 183475.45 | 231639.60 |
| Uracil | 5033.75 | 3415.94 | 5197.17 | 5694.06 |
| Uridine | 36806.34 | 16192.18 | 18415.62 | 21861.97 |
| Urocanic acid | 210.40 | 1553.98 | 2352.60 | 3600.42 |
| Vanillin | 1926.48 | 3046.38 | 4812.70 | 4886.29 |

| Subject | D | D | D | D |
|---|---|---|---|---|
| **Time Point** | **0** | **90** | **MW** | **END** |
| **metabolites** | Sample 9 | Sample 10 | Sample 11 | Sample 12 |
| 1,3 Cyclohexanedione | 1451.70 | 2287.91 | 2239.95 | 2152.85 |
| 1,7-Dimethyluric acid | 106.42 | 3727.28 | 679.37 | 722.00 |
| 1-Methyladenosine | 2157.40 | 7235.59 | 5413.66 | 7451.92 |
| 1-Methylhistidine | 3826.67 | 3371.46 | 2602.73 | 2781.43 |
| 1-Methylnicotinamide | 234.40 | 467.56 | 1243.39 | 638.79 |
| 2'-Deoxyguanosine 5'-monophosphate | 17769.45 | 128.12 | 323.51 | 69.94 |
| 2-Hydroxy-4-methylpentanoic acid | 4522.90 | 4272.79 | 4457.38 | 5257.11 |
| 2-Phenylacetamide | 75857.61 | 102338.29 | 78338.78 | 68028.30 |
| 3-(1-Pyrazolyl)-alanine | 149.61 | 427.93 | 493.88 | 341.72 |
| 3-(2-Hydroxyphenyl)propanoic acid | 5061.98 | 12740.56 | 6725.59 | 9249.59 |
| 3-Hydroxybenzaldehyde | 15089.08 | 22176.45 | 15952.30 | 21030.34 |
| 3-Indoleacetic acid | 7062.11 | 24826.80 | 9904.76 | 26544.85 |
| 3-Indolepropionic acid | 2308.82 | 8203.33 | 5709.24 | 5820.72 |
| 3-Methoxytyrosine | 490.74 | 1352.59 | 951.70 | 882.13 |
| 4-Aminophenol | 28.91 | 48.34 | 56.98 | 44.14 |
| 4-Guanidinobutanoate | 109.21 | 403.59 | 208.79 | 288.93 |
| 4-Hydroxyhippuric acid | 216.71 | 33017.63 | 1151.77 | 2187.60 |
| 4-Hydroxyphenylglycine | 2044.11 | 4830.51 | 3520.60 | 3684.37 |
| 4-Hydroxyproline | 1169.67 | 4191.20 | 1259.68 | 1218.70 |
| 4-Hydroxyquinoline | 1097.43 | 2377.54 | 1628.10 | 1373.61 |
| 4-Imidazoleacetate | 1727.53 | 3588.53 | 6169.16 | 4788.74 |
| 4-Methylcatechol | 964.39 | 520.58 | 496.25 | 197.60 |
| 5-Aminosalicylic acid | 17.13 | 2785.66 | 3605.77 | 3716.61 |
| Acetaminophen glucuronide | 4.24 | 8.50 | 4.24 | 0.00 |
| Acetominophen | 6.52 | 2.66 | 2.94 | 3.15 |
| Acetylcholine | 18772.80 | 24888.82 | 31762.06 | 25140.67 |
| Adenosine | 474.71 | 627.37 | 819.68 | 290.98 |
| Adenosine 3',5'-Diphosphate | 6937.58 | 21.37 | 27.92 | 7.91 |
| Adenosine-3-monophosphate | 6348.70 | 18.23 | 93.96 | 17.71 |
| Adipic acid | 8706.34 | 34.01 | 68.51 | 25.58 |
| Alanine | 1690.46 | 1286.49 | 1286.67 | 1599.29 |
| Arabinose | 10.44 | 1298.86 | 1305.78 | 1273.31 |
| Arginine | 4370.97 | 24137.96 | 18874.43 | 20123.72 |
| Asp-Phe | 193.13 | 482.36 | 259.36 | 358.28 |
| Benzoic acid | 117.15 | 316.91 | 177.82 | 133.45 |
| Betaine | 76040.33 | 87526.18 | 66925.82 | 76423.90 |
| Biliverdin | 4.65 | 25.82 | 72.06 | 1771.13 |
| Cadaverine | 639.46 | 502.89 | 954.26 | 758.86 |
| Caffeine | 2626.45 | 273532.60 | 29498.46 | 20254.00 |
| Carnitine | 5495.37 | 4897.23 | 8561.21 | 5573.13 |
| Cholic acid | 1009.62 | 2052.11 | 4681.97 | 2268.33 |
| Choline | 143266.75 | 234192.15 | 208514.45 | 195219.70 |
| Citrulline | 3311.53 | 7970.48 | 5617.27 | 5458.08 |
| CMPF | 146978.05 | 593883.45 | 598679.35 | 542482.20 |
| Cortisol | 18300.42 | 27247.08 | 41452.42 | 33433.34 |
| Cortisone | 2078.34 | 3559.52 | 3981.31 | 3602.58 |
| Creatine | 122882.52 | 77030.57 | 102434.54 | 108002.02 |

| Subject | D | D | D | D |
|---|---|---|---|---|
| Time Point | 0 | 90 | MW | END |
| **metabolites** | Sample 9 | Sample 10 | Sample 11 | Sample 12 |
| Creatinine | 39172.13 | 80096.59 | 63325.75 | 62860.05 |
| Cyclo(Leu-Pro) | 3126.75 | 9811.25 | 4113.63 | 2900.19 |
| Cyclohexanamine | 303.40 | 1172.90 | 798.90 | 1456.77 |
| Cystine | 911.42 | 815.51 | 1042.97 | 862.05 |
| Dehydroisoandrosterone sulfate | 457790.85 | 546213.05 | 323776.80 | 313971.00 |
| delta-Trimethyllysine | 964.61 | 784.29 | 1831.55 | 621.10 |
| Docosahexanoic acid | 147373.90 | 68088.35 | 107058.55 | 129767.05 |
| Dopamine | 473.70 | 813.54 | 591.33 | 516.51 |
| Eicosenoic acid | 7345.73 | 1410.07 | 2969.59 | 3777.74 |
| Ergothioneine | 9836.72 | 2237.37 | 3078.42 | 2806.66 |
| Ethanolamine | 1327.17 | 2476.17 | 3509.27 | 2063.45 |
| Ethyl myristate | 729346.65 | 189021.45 | 422065.70 | 396988.75 |
| Galactosamine | 402.48 | 267.11 | 267.80 | 328.73 |
| gamma-Glutamylleucine | 13477.38 | 60002.63 | 29420.90 | 33888.40 |
| gamma-Glutamyltyrosine | 2498.66 | 10376.34 | 5399.58 | 7507.20 |
| Glutamic acid | 4486.13 | 2901.60 | 3918.82 | 4173.60 |
| Glutamine | 7720.50 | 12191.57 | 11814.70 | 10348.95 |
| Glutarylcarnitine | 3092.50 | 3019.79 | 2551.28 | 1786.63 |
| Glu-Thr | 3318.08 | 549.10 | 583.53 | 402.08 |
| Glyceraldehyde | 156297.90 | 113189.65 | 56528.98 | 55074.78 |
| Glycochenodeoxycholate | 37745.46 | 249061.80 | 41038.62 | 63434.70 |
| Glycocholate | 2367.80 | 22569.52 | 7060.58 | 13190.57 |
| Guanosine | 17.84 | 340.28 | 61.79 | 433.00 |
| Histamine | 727.25 | 427.03 | 476.27 | 478.85 |
| Histidine | 2837.44 | 3267.48 | 2720.12 | 2962.46 |
| Homocysteine | 253.03 | 17161.00 | 12910.79 | 12722.49 |
| Hypoxanthine | 510166.90 | 67573.82 | 28208.51 | 86174.96 |
| Indole-3-carboxaldehyde | 27900.60 | 56878.24 | 53845.59 | 62075.84 |
| Indolelactic acid | 19111.22 | 27232.68 | 23648.07 | 28059.54 |
| Inosine-5-monophosphate | 6308.72 | 79.11 | 44.73 | 20.27 |
| Kynurenic acid | 1229.47 | 1665.55 | 1155.92 | 1799.04 |
| Kynurenine | 17240.44 | 30046.08 | 20704.82 | 18424.93 |
| Lactose | 0.00 | 3627.85 | 1665.99 | 2023.06 |
| Lauroylcarnitine | 6187.47 | 4122.27 | 7750.99 | 7975.30 |
| Leucine | 234643.70 | 317211.05 | 247292.50 | 261397.15 |
| Linoleic acid | 414128.20 | 327821.75 | 258411.10 | 360113.35 |
| Lysine | 3885.89 | 5696.97 | 5806.90 | 5165.76 |
| Mannose | 119855.90 | 526081.15 | 541404.70 | 491907.00 |
| Methionine | 19187.51 | 39061.69 | 30230.00 | 46119.49 |
| Methyl Heptadecanoic acid | 218543.15 | 61651.56 | 186927.39 | 132674.19 |
| Methylguanidine | 855.07 | 720.30 | 672.77 | 705.88 |
| Methyltestosterone | 25044.30 | 30126.49 | 18678.02 | 18177.96 |
| Methylthioadenosine | 264.22 | 1604.85 | 788.70 | 1264.73 |
| N,N-Dimethylarginine | 28049.63 | 2403.39 | 4161.05 | 3292.07 |
| N2,N2-Dimethylguanosine | 702.23 | 1445.50 | 1088.85 | 1555.31 |
| N-Acetylglucosamine | 0.00 | 1056.86 | 788.61 | 992.33 |
| N-Acetylputrescine | 854.99 | 408.83 | 408.29 | 435.68 |

| Subject | D | D | D | D |
|---|---|---|---|---|
| Time Point | 0 | 90 | MW | END |
| **metabolites** | Sample 9 | Sample 10 | Sample 11 | Sample 12 |
| N-epsilon-Acetyllysine | 1042.03 | 3266.77 | 1740.98 | 2071.78 |
| Nicotinamide | 8827.48 | 6308.04 | 4813.47 | 2340.48 |
| N-Methylproline | 721.93 | 4201.45 | 835.15 | 579.14 |
| Noradrenaline | 786.47 | 1618.37 | 1407.16 | 1290.79 |
| Norleucine | 137777.80 | 218547.00 | 149014.65 | 160676.70 |
| Norvaline | 103887.24 | 163635.70 | 134681.10 | 134723.15 |
| O-Acetylcarnitine | 174165.50 | 163245.95 | 182716.80 | 167437.30 |
| O-Acetylserine | 14738.55 | 44893.11 | 40912.20 | 30059.64 |
| Octopine | 428.93 | 1213.49 | 623.31 | 883.22 |
| Olmesartan | 35.50 | 38.09 | 20.04 | 26.66 |
| Ornithine | 3381.76 | 4551.39 | 2253.92 | 1488.06 |
| Palmitoylcarnitine | 16477.40 | 12832.93 | 16627.68 | 17799.02 |
| Pantothenic acid | 1153.10 | 2270.69 | 2422.72 | 3565.13 |
| Paraxanthine | 2952.15 | 223262.95 | 30676.05 | 27105.24 |
| Phenylacetaldehyde | 695.00 | 786.73 | 363.77 | 1609.52 |
| Phenylacetylglutamine | 36081.20 | 100263.33 | 133404.69 | 207272.30 |
| Phenylalanine | 596722.50 | 1024940.00 | 684932.95 | 864997.15 |
| Phenylethanolamine | 166.78 | 402.96 | 368.44 | 357.97 |
| Phosphocholine | 84740.41 | 47265.53 | 68016.44 | 61494.24 |
| Phosphoric acid | 17741.86 | 56392.78 | 44348.34 | 42841.22 |
| P-Hydroxyphenylacetate | 222.69 | 550.54 | 333.25 | 683.80 |
| Pipecolate | 8288.59 | 19490.87 | 33142.93 | 16050.06 |
| Piperine | 2776.33 | 38989.89 | 79277.73 | 10090.71 |
| Proline | 84420.60 | 315698.95 | 146071.10 | 146889.80 |
| Propanoate | 3575.76 | 10914.37 | 17230.52 | 14175.63 |
| Propionylcarnitine | 16657.06 | 28453.48 | 29692.79 | 18268.00 |
| Putrescine | 892.96 | 576.53 | 661.11 | 529.94 |
| Pyridoxamine | 2759.59 | 2038.29 | 2679.13 | 2735.84 |
| Pyroglutamic acid | 14715.54 | 23042.00 | 21217.14 | 20327.87 |
| Quinoline | 5155.24 | 16698.90 | 7454.50 | 20252.07 |
| Resorcinol Monoacetate | 2799.62 | 4897.47 | 5544.94 | 6275.76 |
| Riboflavin | 218.10 | 790.60 | 751.14 | 348.80 |
| Ribose 5-Phosphate | 142.31 | 4363.00 | 2859.14 | 3362.42 |
| Sedoheptulose | 458.82 | 174.30 | 119.73 | 179.73 |
| Serine | 656.77 | 701.76 | 704.34 | 639.93 |
| Spermine | 1145.13 | 133.90 | 142.90 | 610.76 |
| Taurine | 6660.17 | 4732.93 | 4203.30 | 4760.57 |
| Theobromine | 7316.03 | 236391.20 | 68698.92 | 25003.60 |
| Theophylline | 682.69 | 25191.13 | 5218.72 | 5055.07 |
| Thiazolidine-4-carboxylic acid | 5383.83 | 5968.95 | 3820.18 | 4039.70 |
| Threitol | 1016.18 | 2023.37 | 2889.00 | 1781.30 |
| Threonic acid | 911.92 | 13682.05 | 9313.88 | 17041.22 |
| Threonine | 1328.04 | 1563.81 | 1439.51 | 1434.60 |
| Trigonelline | 2978.76 | 19030.32 | 7831.13 | 39000.61 |
| Trimethylamine N-oxide | 1455.56 | 3247.37 | 4566.58 | 6474.73 |
| Tryptamine | 17645.25 | 36211.24 | 31525.15 | 36926.80 |
| Tryptophan | 1047988.70 | 2230690.00 | 1838382.00 | 2223190.00 |

| Subject | D | D | D | D |
|---|---|---|---|---|
| Time Point | 0 | 90 | MW | END |
| **metabolites** | Sample 9 | Sample 10 | Sample 11 | Sample 12 |
| Tyrosine | 198530.25 | 298742.70 | 231034.30 | 176511.69 |
| Uracil | 9838.65 | 2303.29 | 3586.66 | 2837.84 |
| Uridine | 53464.24 | 9733.77 | 15943.64 | 10949.41 |
| Urocanic acid | 782.01 | 1971.81 | 1144.65 | 2312.15 |
| Vanillin | 2669.09 | 4133.11 | 4408.18 | 4006.80 |

| Subject | F | F | F | F |
|---|---|---|---|---|
| Time Point | 0 | 90 | MW | END |
| **metabolites** | Sample 13 | Sample 14 | Sample 15 | Sample 16 |
| 1,3 Cyclohexanedione | 1388.35 | 2434.05 | 2819.74 | 1933.09 |
| 1,7-Dimethyluric acid | 680.62 | 7578.52 | 9189.90 | 2225.10 |
| 1-Methyladenosine | 2061.76 | 7112.34 | 3855.41 | 3994.50 |
| 1-Methylhistidine | 3271.04 | 2750.21 | 3564.84 | 4210.55 |
| 1-Methylnicotinamide | 160.18 | 2961.30 | 1669.43 | 2268.69 |
| 2'-Deoxyguanosine 5'-monophosphate | 11616.53 | 191.45 | 77.23 | 100.65 |
| 2-Hydroxy-4-methylpentanoic acid | 8379.62 | 4793.85 | 12167.10 | 7953.46 |
| 2-Phenylacetamide | 56810.96 | 113647.35 | 81760.92 | 56406.18 |
| 3-(1-Pyrazolyl)-alanine | 298.89 | 695.58 | 497.00 | 423.10 |
| 3-(2-Hydroxyphenyl)propanoic acid | 11805.67 | 7549.82 | 30793.04 | 4908.48 |
| 3-Hydroxybenzaldehyde | 11381.34 | 25025.47 | 16701.81 | 19249.66 |
| 3-Indoleacetic acid | 8441.39 | 22465.19 | 18504.94 | 23052.82 |
| 3-Indolepropionic acid | 5066.15 | 22604.97 | 2559.89 | 140.40 |
| 3-Methoxytyrosine | 398.01 | 1261.64 | 691.46 | 1031.73 |
| 4-Aminophenol | 25.38 | 48.56 | 25804.98 | 41.43 |
| 4-Guanidinobutanoate | 144.90 | 284.39 | 248.03 | 154.23 |
| 4-Hydroxyhippuric acid | 23.20 | 91.13 | 122.52 | 70.06 |
| 4-Hydroxyphenylglycine | 2225.28 | 5145.24 | 3934.05 | 4135.10 |
| 4-Hydroxyproline | 1091.45 | 932.50 | 751.64 | 1212.50 |
| 4-Hydroxyquinoline | 734.50 | 1694.35 | 1286.65 | 1215.30 |
| 4-Imidazoleacetate | 1497.98 | 5741.90 | 4396.32 | 7120.09 |
| 4-Methylcatechol | 462.19 | 373.26 | 45.57 | 40.87 |
| 5-Aminosalicylic acid | 11.19 | 2089.30 | 1567.96 | 1252.42 |
| Acetaminophen glucuronide | 4.21 | 3.28 | 45306.27 | 3.89 |
| Acetominophen | 2.62 | 2.67 | 18129.02 | 6.28 |
| Acetylcholine | 14776.98 | 21191.64 | 15702.32 | 22276.46 |
| Adenosine | 416.31 | 1219.66 | 299.52 | 1170.14 |
| Adenosine 3',5'-Diphosphate | 4639.21 | 19.12 | 20.78 | 33.41 |
| Adenosine-3-monophosphate | 5405.29 | 19.40 | 20.95 | 49.68 |
| Adipic acid | 10617.08 | 48.96 | 76.81 | 38.82 |
| Alanine | 1357.70 | 1063.46 | 952.85 | 859.94 |
| Arabinose | 60.28 | 2144.64 | 2100.27 | 1546.53 |
| Arginine | 4998.03 | 22756.14 | 32988.43 | 30855.21 |
| Asp-Phe | 185.55 | 417.19 | 250.97 | 491.08 |
| Benzoic acid | 1186.81 | 566.51 | 7767.33 | 19.87 |
| Betaine | 61414.09 | 79574.20 | 68819.37 | 81967.43 |
| Biliverdin | 3.70 | 33.72 | 3.22 | 19.89 |
| Cadaverine | 624.08 | 895.57 | 1471.34 | 1131.10 |
| Caffeine | 31700.89 | 151863.85 | 310430.80 | 63534.33 |
| Carnitine | 4242.53 | 6262.95 | 6289.63 | 5464.37 |
| Cholic acid | 1987.68 | 2513.00 | 11736.59 | 9476.89 |
| Choline | 121608.10 | 245964.85 | 182511.10 | 217435.05 |
| Citrulline | 2723.59 | 6894.72 | 4498.62 | 5120.07 |
| CMPF | 82668.59 | 160903.25 | 137680.65 | 82981.01 |
| Cortisol | 25437.20 | 38645.05 | 33156.53 | 50058.58 |
| Cortisone | 2569.96 | 4215.41 | 4598.51 | 4233.32 |
| Creatine | 68613.08 | 64279.09 | 47784.82 | 36581.29 |

| Subject | F | F | F | F |
|---|---|---|---|---|
| Time Point | 0 | 90 | MW | END |
| **metabolites** | Sample 13 | Sample 14 | Sample 15 | Sample 16 |
| Creatinine | 53111.57 | 93497.83 | 63611.91 | 68720.28 |
| Cyclo(Leu-Pro) | 8803.60 | 37531.22 | 38149.28 | 32061.57 |
| Cyclohexanamine | 435.56 | 591.33 | 483.74 | 767.60 |
| Cystine | 463.78 | 893.64 | 1044.79 | 1078.29 |
| Dehydroisoandrosterone sulfate | 2925884.00 | 1169860.30 | 1181320.50 | 750586.35 |
| delta-Trimethyllysine | 740.17 | 970.43 | 1151.39 | 1798.08 |
| Docosahexanoic acid | 61654.88 | 52198.37 | 145274.64 | 97671.41 |
| Dopamine | 364.15 | 689.74 | 546.26 | 506.74 |
| Eicosenoic acid | 2703.78 | 2097.01 | 12646.69 | 7088.32 |
| Ergothioneine | 6364.39 | 1178.61 | 873.35 | 1866.37 |
| Ethanolamine | 1299.52 | 3160.40 | 1830.65 | 2380.11 |
| Ethyl myristate | 420616.65 | 195059.95 | 888822.30 | 695746.95 |
| Galactosamine | 282.05 | 263.11 | 206.61 | 296.95 |
| gamma-Glutamylleucine | 16775.07 | 48346.15 | 39158.74 | 40126.10 |
| gamma-Glutamyltyrosine | 2061.35 | 9374.82 | 6238.00 | 8893.58 |
| Glutamic acid | 4401.05 | 4512.30 | 2868.84 | 4724.71 |
| Glutamine | 8063.44 | 12740.82 | 13200.11 | 18453.01 |
| Glutarylcarnitine | 2641.95 | 2561.49 | 2164.81 | 1378.24 |
| Glu-Thr | 2701.91 | 682.41 | 808.56 | 735.75 |
| Glyceraldehyde | 190387.00 | 63368.26 | 47884.55 | 32027.51 |
| Glycochenodeoxycholate | 87507.28 | 139009.10 | 254619.60 | 178544.05 |
| Glycocholate | 7569.25 | 17446.17 | 42534.74 | 23792.58 |
| Guanosine | 41.96 | 431.59 | 35.29 | 272.45 |
| Histamine | 532.39 | 467.23 | 453.70 | 488.49 |
| Histidine | 2747.13 | 2238.16 | 5606.33 | 5402.33 |
| Homocysteine | 657.65 | 19514.12 | 9496.98 | 10320.19 |
| Hypoxanthine | 406148.49 | 39698.58 | 14945.10 | 38398.96 |
| Indole-3-carboxaldehyde | 25271.34 | 58442.49 | 48722.37 | 43118.21 |
| Indolelactic acid | 24801.39 | 24464.82 | 47979.48 | 18235.19 |
| Inosine-5-monophosphate | 4391.94 | 69.39 | 39.89 | 28.21 |
| Kynurenic acid | 646.22 | 1136.10 | 2106.85 | 1166.91 |
| Kynurenine | 10002.79 | 23914.69 | 18066.11 | 11469.72 |
| Lactose | 39.22 | 3509.95 | 1351.59 | 2926.44 |
| Lauroylcarnitine | 2391.77 | 10021.31 | 14939.97 | 17802.25 |
| Leucine | 223249.45 | 312367.80 | 256788.60 | 258938.70 |
| Linoleic acid | 135753.44 | 141654.63 | 689763.95 | 697454.25 |
| Lysine | 2865.82 | 5754.73 | 3951.56 | 5131.67 |
| Mannose | 137777.83 | 673122.30 | 396909.50 | 518744.75 |
| Methionine | 9574.20 | 42741.09 | 30442.50 | 49722.96 |
| Methyl Heptadecanoic acid | 172601.93 | 89216.91 | 251721.85 | 261331.35 |
| Methylguanidine | 898.41 | 885.75 | 489.36 | 739.36 |
| Methyltestosterone | 8461.01 | 13373.25 | 49877.69 | 39287.98 |
| Methylthioadenosine | 293.97 | 1631.52 | 1195.90 | 1169.38 |
| N,N-Dimethylarginine | 12827.22 | 1712.52 | 2574.31 | 1786.34 |
| N2,N2-Dimethylguanosine | 807.87 | 1591.34 | 1230.77 | 1076.37 |
| N-Acetylglucosamine | 13.03 | 1153.34 | 607.66 | 848.82 |
| N-Acetylputrescine | 537.22 | 547.05 | 360.04 | 379.43 |

| Subject | F | F | F | F |
|---|---|---|---|---|
| Time Point | 0 | 90 | MW | END |
| **metabolites** | Sample 13 | Sample 14 | Sample 15 | Sample 16 |
| N-epsilon-Acetyllysine | 530.56 | 2046.42 | 1179.16 | 1475.94 |
| Nicotinamide | 9322.51 | 10703.13 | 2223.02 | 982.85 |
| N-Methylproline | 798.34 | 5523.80 | 554.18 | 2409.47 |
| Noradrenaline | 918.87 | 1626.89 | 1138.12 | 1962.40 |
| Norleucine | 131892.74 | 198675.80 | 162331.30 | 183628.90 |
| Norvaline | 97200.71 | 189082.10 | 129531.60 | 152326.55 |
| O-Acetylcarnitine | 112591.02 | 167477.25 | 173796.65 | 161793.45 |
| O-Acetylserine | 18421.20 | 53471.12 | 32100.60 | 18154.56 |
| Octopine | 326.70 | 956.27 | 609.59 | 831.66 |
| Olmesartan | 40.78 | 31.08 | 17.36 | 23.62 |
| Ornithine | 2581.68 | 2804.25 | 1824.20 | 2174.20 |
| Palmitoylcarnitine | 3830.67 | 4864.70 | 8109.55 | 9198.25 |
| Pantothenic acid | 1271.41 | 3210.12 | 2681.30 | 1570.80 |
| Paraxanthine | 81637.39 | 461780.15 | 455905.00 | 184410.60 |
| Phenylacetaldehyde | 1775.09 | 1868.11 | 4107.30 | 1921.20 |
| Phenylacetylglutamine | 5371.40 | 84111.41 | 9874.32 | 25782.56 |
| Phenylalanine | 495654.85 | 889482.75 | 652531.55 | 639866.30 |
| Phenylethanolamine | 152.95 | 506.18 | 510.47 | 634.45 |
| Phosphocholine | 94350.68 | 51250.27 | 73172.07 | 65427.62 |
| Phosphoric acid | 19498.33 | 49777.07 | 31453.51 | 63493.35 |
| P-Hydroxyphenylacetate | 187.54 | 380.43 | 245.99 | 631.74 |
| Pipecolate | 9052.02 | 31061.86 | 16229.23 | 22757.14 |
| Piperine | 1699.90 | 949.72 | 1984.65 | 96.22 |
| Proline | 122261.39 | 244781.75 | 138599.90 | 192086.55 |
| Propanoate | 3228.18 | 16911.25 | 12848.05 | 21990.19 |
| Propionylcarnitine | 5631.78 | 30482.73 | 12832.17 | 7523.48 |
| Putrescine | 726.88 | 915.39 | 529.91 | 655.57 |
| Pyridoxamine | 1903.65 | 2794.55 | 1981.63 | 3007.72 |
| Pyroglutamic acid | 14984.07 | 26235.10 | 23710.25 | 34292.56 |
| Quinoline | 6196.30 | 17032.91 | 13487.30 | 17362.42 |
| Resorcinol Monoacetate | 2743.77 | 5506.87 | 6023.56 | 5547.78 |
| Riboflavin | 232.39 | 547.77 | 498.08 | 356.15 |
| Ribose 5-Phosphate | 455.23 | 4960.43 | 2317.42 | 4664.81 |
| Sedoheptulose | 1007.22 | 170.61 | 115.42 | 140.09 |
| Serine | 641.22 | 927.80 | 823.76 | 910.69 |
| Spermine | 746.21 | 225.07 | 149.28 | 351.33 |
| Taurine | 5612.74 | 4205.94 | 3797.18 | 6085.97 |
| Theobromine | 59585.65 | 209085.95 | 194148.20 | 100181.15 |
| Theophylline | 9967.69 | 77818.28 | 80298.34 | 22325.69 |
| Thiazolidine-4-carboxylic acid | 5074.84 | 5794.58 | 3288.36 | 6254.14 |
| Threitol | 1027.63 | 2975.48 | 1996.85 | 3028.48 |
| Threonic acid | 1487.41 | 15900.82 | 18785.02 | 7474.44 |
| Threonine | 1313.67 | 1816.19 | 1416.21 | 2261.57 |
| Trigonelline | 4579.42 | 39346.58 | 15593.76 | 13329.76 |
| Trimethylamine N-oxide | 1583.68 | 2064.99 | 1576.26 | 127.69 |
| Tryptamine | 16057.74 | 37824.80 | 28137.80 | 27134.54 |
| Tryptophan | 983866.40 | 2285268.50 | 1689525.00 | 1559894.00 |

| Subject | F | F | F | F |
|---|---|---|---|---|
| **Time Point** | **0** | **90** | **MW** | **END** |
| **metabolites** | Sample 13 | Sample 14 | Sample 15 | Sample 16 |
| Tyrosine | 139365.74 | 311316.70 | 229986.40 | 151924.10 |
| Uracil | 7935.99 | 6417.03 | 4168.77 | 1238.23 |
| Uridine | 43356.10 | 27345.86 | 16604.04 | 4852.82 |
| Urocanic acid | 637.46 | 1503.90 | 1007.56 | 1656.64 |
| Vanillin | 2556.32 | 3865.19 | 4215.44 | 3777.19 |

| Subject | I | I | I | I |
|---|---|---|---|---|
| **Time Point** | **0** | **90** | **MW** | **END** |
| **metabolites** | Sample 17 | Sample 18 | Sample 19 | Sample 20 |
| 1,3 Cyclohexanedione | 1286.15 | 2733.18 | 3061.09 | 1521.44 |
| 1,7-Dimethyluric acid | 733.69 | 1782.12 | 1996.58 | 1002.79 |
| 1-Methyladenosine | 2460.21 | 6725.67 | 12640.41 | 4391.03 |
| 1-Methylhistidine | 3974.62 | 2495.98 | 3086.17 | 2783.25 |
| 1-Methylnicotinamide | 109.14 | 579.76 | 2378.50 | 804.83 |
| 2'-Deoxyguanosine 5'-monophosphate | 8404.36 | 133.71 | 136.38 | 77.15 |
| 2-Hydroxy-4-methylpentanoic acid | 6413.09 | 9133.15 | 8019.91 | 4538.07 |
| 2-Phenylacetamide | 57969.78 | 86408.10 | 114036.70 | 38581.05 |
| 3-(1-Pyrazolyl)-alanine | 105.98 | 580.25 | 763.94 | 357.13 |
| 3-(2-Hydroxyphenyl)propanoic acid | 10211.73 | 11063.72 | 8057.71 | 4682.54 |
| 3-Hydroxybenzaldehyde | 11446.00 | 19041.83 | 22980.27 | 8567.62 |
| 3-Indoleacetic acid | 17205.80 | 51077.45 | 79697.12 | 48585.77 |
| 3-Indolepropionic acid | 9155.22 | 6579.26 | 8717.14 | 6019.90 |
| 3-Methoxytyrosine | 456.26 | 970.26 | 1026.46 | 566.44 |
| 4-Aminophenol | 27.62 | 41.15 | 47.41 | 57.13 |
| 4-Guanidinobutanoate | 57.78 | 291.31 | 197.44 | 122.61 |
| 4-Hydroxyhippuric acid | 28.16 | 207.30 | 875.36 | 1576.25 |
| 4-Hydroxyphenylglycine | 2236.19 | 4618.64 | 4819.22 | 2966.37 |
| 4-Hydroxyproline | 1999.18 | 1182.03 | 972.97 | 822.06 |
| 4-Hydroxyquinoline | 938.38 | 1364.18 | 1984.69 | 538.37 |
| 4-Imidazoleacetate | 1631.33 | 3527.99 | 4078.08 | 4098.96 |
| 4-Methylcatechol | 536.78 | 338.38 | 256.12 | 344.04 |
| 5-Aminosalicylic acid | 50.11 | 1939.30 | 2125.98 | 778.34 |
| Acetaminophen glucuronide | 2.76 | 0.00 | 7.29 | 3.70 |
| Acetominophen | 5.26 | 6.82 | 4.71 | 6.27 |
| Acetylcholine | 14358.04 | 32536.79 | 52513.05 | 31560.73 |
| Adenosine | 406.36 | 869.90 | 458.51 | 919.39 |
| Adenosine 3',5'-Diphosphate | 3218.80 | 17.13 | 33.43 | 8.08 |
| Adenosine-3-monophosphate | 7876.77 | 20.96 | 19.92 | 31.21 |
| Adipic acid | 10712.07 | 57.22 | 83.92 | 19.02 |
| Alanine | 882.05 | 920.26 | 1001.07 | 714.42 |
| Arabinose | 0.00 | 2179.53 | 3092.77 | 1083.87 |
| Arginine | 9900.54 | 16321.73 | 17424.34 | 13648.67 |
| Asp-Phe | 210.10 | 430.43 | 307.06 | 241.12 |
| Benzoic acid | 319.35 | 167.73 | 407.90 | 311.06 |
| Betaine | 72360.15 | 72879.75 | 83929.84 | 61361.72 |
| Biliverdin | 9.15 | 24.50 | 11447.86 | 595.48 |
| Cadaverine | 836.75 | 816.81 | 827.70 | 1070.27 |
| Caffeine | 60677.75 | 582805.15 | 125883.55 | 48229.39 |
| Carnitine | 4914.23 | 5741.07 | 10607.46 | 4439.44 |
| Cholic acid | 9346.27 | 210529.45 | 3041.89 | 594.15 |
| Choline | 128185.15 | 189172.70 | 230034.00 | 199111.20 |
| Citrulline | 2967.06 | 3801.58 | 4793.94 | 2904.71 |
| CMPF | 316063.50 | 87508.83 | 170219.90 | 54429.01 |
| Cortisol | 16811.63 | 19624.45 | 46868.59 | 23085.83 |
| Cortisone | 2442.26 | 3659.57 | 5524.53 | 3992.47 |
| Creatine | 45249.29 | 41929.41 | 51437.58 | 25531.32 |

| Subject | I | I | I | I |
|---|---|---|---|---|
| **Time Point** | **0** | **90** | **MW** | **END** |
| **metabolites** | Sample 17 | Sample 18 | Sample 19 | Sample 20 |
| Creatinine | 38779.70 | 90731.17 | 117042.85 | 62563.32 |
| Cyclo(Leu-Pro) | 1881.62 | 4631.26 | 4830.75 | 2406.44 |
| Cyclohexanamine | 389.77 | 906.10 | 1150.06 | 1157.78 |
| Cystine | 715.45 | 784.10 | 1305.28 | 1053.05 |
| Dehydroisoandrosterone sulfate | 2261872.50 | 1041153.55 | 709902.40 | 427863.85 |
| delta-Trimethyllysine | 1540.61 | 889.25 | 950.74 | 1025.25 |
| Docosahexanoic acid | 94389.46 | 59362.39 | 57939.79 | 92444.44 |
| Dopamine | 454.89 | 611.90 | 712.63 | 257.49 |
| Eicosenoic acid | 1734.10 | 1549.22 | 2661.78 | 10566.64 |
| Ergothioneine | 4877.05 | 2084.02 | 2899.25 | 1522.61 |
| Ethanolamine | 1435.95 | 1569.21 | 5528.66 | 1931.90 |
| Ethyl myristate | 263934.35 | 395525.50 | 309520.50 | 506037.10 |
| Galactosamine | 449.92 | 250.09 | 360.47 | 207.04 |
| gamma-Glutamylleucine | 14787.42 | 72316.65 | 66555.68 | 32332.76 |
| gamma-Glutamyltyrosine | 2606.02 | 10228.42 | 9387.05 | 5950.39 |
| Glutamic acid | 2620.73 | 1638.90 | 2640.85 | 1819.71 |
| Glutamine | 9570.05 | 11833.02 | 14857.18 | 14471.30 |
| Glutarylcarnitine | 2075.06 | 2121.08 | 4285.49 | 1869.69 |
| Glu-Thr | 3553.09 | 413.37 | 1009.39 | 442.81 |
| Glyceraldehyde | 194031.55 | 49673.22 | 72021.03 | 50381.78 |
| Glycochenodeoxycholate | 23123.83 | 257981.80 | 38857.77 | 21008.98 |
| Glycocholate | 10150.90 | 36682.58 | 5178.64 | 2227.50 |
| Guanosine | 6.57 | 214.26 | 42.45 | 87.69 |
| Histamine | 532.51 | 420.95 | 490.36 | 440.10 |
| Histidine | 2854.68 | 2813.90 | 4925.34 | 4412.32 |
| Homocysteine | 270.90 | 19562.77 | 26389.99 | 8404.08 |
| Hypoxanthine | 169305.40 | 16378.86 | 24106.17 | 34333.40 |
| Indole-3-carboxaldehyde | 27130.78 | 52709.88 | 78245.42 | 42519.13 |
| Indolelactic acid | 25606.88 | 29913.41 | 25516.78 | 13608.11 |
| Inosine-5-monophosphate | 7863.41 | 32.97 | 74.03 | 15.21 |
| Kynurenic acid | 1131.55 | 1631.00 | 1615.73 | 909.22 |
| Kynurenine | 12199.08 | 20090.50 | 28428.85 | 7155.14 |
| Lactose | 0.00 | 7208.15 | 4340.71 | 7235.79 |
| Lauroylcarnitine | 4179.95 | 10359.55 | 29783.97 | 11273.61 |
| Leucine | 201033.75 | 326845.15 | 367701.80 | 178050.61 |
| Linoleic acid | 105171.66 | 196324.60 | 238723.95 | 402492.05 |
| Lysine | 4061.45 | 4612.01 | 5080.03 | 4070.37 |
| Mannose | 120918.25 | 437476.55 | 629301.85 | 323776.25 |
| Methionine | 13379.78 | 35675.15 | 52985.15 | 28760.18 |
| Methyl Heptadecanoic acid | 100830.46 | 182011.55 | 101799.44 | 146148.68 |
| Methylguanidine | 779.81 | 627.19 | 875.57 | 603.19 |
| Methyltestosterone | 9740.01 | 10070.48 | 18008.86 | 30146.83 |
| Methylthioadenosine | 262.70 | 1492.26 | 1438.39 | 846.91 |
| N,N-Dimethylarginine | 14680.55 | 7362.58 | 10545.55 | 15031.27 |
| N2,N2-Dimethylguanosine | 732.00 | 1109.83 | 1715.50 | 890.62 |
| N-Acetylglucosamine | 0.00 | 1000.17 | 1474.17 | 593.91 |
| N-Acetylputrescine | 446.60 | 381.10 | 529.18 | 282.99 |

| Subject | I | I | I | I |
|---:|---:|---:|---:|---:|
| Time Point | 0 | 90 | MW | END |
| **metabolites** | Sample 17 | Sample 18 | Sample 19 | Sample 20 |
| N-epsilon-Acetyllysine | 1043.68 | 1751.68 | 1721.80 | 1099.97 |
| Nicotinamide | 4378.58 | 2055.58 | 3322.40 | 975.66 |
| N-Methylproline | 545.14 | 2030.31 | 2805.90 | 2072.44 |
| Noradrenaline | 957.43 | 1253.34 | 2073.40 | 1338.16 |
| Norleucine | 109821.25 | 226726.60 | 229428.55 | 111198.27 |
| Norvaline | 83455.12 | 180055.80 | 221700.55 | 125796.45 |
| O-Acetylcarnitine | 99795.58 | 146700.78 | 202686.30 | 123716.62 |
| O-Acetylserine | 46419.03 | 31720.40 | 62012.10 | 21901.20 |
| Octopine | 387.12 | 1177.13 | 1396.85 | 624.20 |
| Olmesartan | 38.51 | 34.12 | 13.50 | 9.32 |
| Ornithine | 3061.68 | 2598.13 | 2015.32 | 1849.34 |
| Palmitoylcarnitine | 3105.42 | 8806.60 | 16548.00 | 7892.72 |
| Pantothenic acid | 907.72 | 1774.52 | 2191.86 | 1365.09 |
| Paraxanthine | 78071.09 | 157253.35 | 146307.35 | 68775.07 |
| Phenylacetaldehyde | 883.61 | 4149.41 | 540.01 | 281.01 |
| Phenylacetylglutamine | 27712.92 | 119115.06 | 200829.65 | 96989.84 |
| Phenylalanine | 460682.35 | 824199.80 | 1082096.50 | 417726.85 |
| Phenylethanolamine | 136.56 | 422.04 | 479.20 | 314.24 |
| Phosphocholine | 56338.19 | 53423.58 | 67447.84 | 83885.79 |
| Phosphoric acid | 16125.36 | 52569.77 | 36500.39 | 41374.33 |
| P-Hydroxyphenylacetate | 300.82 | 3021.30 | 277.92 | 309.71 |
| Pipecolate | 7946.65 | 13113.92 | 53874.81 | 15558.21 |
| Piperine | 4289.34 | 2806.63 | 1235.05 | 7249.56 |
| Proline | 128752.05 | 281079.90 | 317597.65 | 171897.90 |
| Propanoate | 3672.89 | 10097.25 | 11274.00 | 12709.63 |
| Propionylcarnitine | 15164.52 | 37026.33 | 39325.33 | 3198.49 |
| Putrescine | 788.58 | 602.37 | 782.25 | 738.79 |
| Pyridoxamine | 2549.96 | 2569.95 | 3166.54 | 2643.89 |
| Pyroglutamic acid | 17085.17 | 20896.58 | 24785.72 | 25167.55 |
| Quinoline | 12689.68 | 37093.96 | 60027.29 | 35375.82 |
| Resorcinol Monoacetate | 2911.17 | 5634.81 | 7438.64 | 5204.07 |
| Riboflavin | 305.37 | 1008.88 | 905.18 | 299.66 |
| Ribose 5-Phosphate | 445.71 | 1153.38 | 1783.10 | 718.61 |
| Sedoheptulose | 1046.48 | 121.05 | 202.21 | 82.64 |
| Serine | 685.04 | 730.23 | 829.34 | 788.87 |
| Spermine | 700.73 | 357.05 | 235.76 | 170.80 |
| Taurine | 5397.09 | 2517.96 | 3991.34 | 4384.98 |
| Theobromine | 34340.50 | 155440.50 | 69374.21 | 45100.37 |
| Theophylline | 11860.61 | 10883.93 | 19431.12 | 7000.62 |
| Thiazolidine-4-carboxylic acid | 5297.16 | 3943.95 | 5581.29 | 1160.94 |
| Threitol | 880.72 | 1861.97 | 2196.26 | 1832.24 |
| Threonic acid | 3001.71 | 19678.28 | 19364.38 | 11080.03 |
| Threonine | 1870.75 | 1434.49 | 1970.58 | 1423.78 |
| Trigonelline | 5351.88 | 18376.13 | 16893.67 | 7185.03 |
| Trimethylamine N-oxide | 1422.31 | 2130.49 | 5127.75 | 2284.61 |
| Tryptamine | 16741.03 | 32270.05 | 45555.53 | 24933.84 |
| Tryptophan | 1037008.50 | 1970552.50 | 2718954.00 | 1540119.50 |

| Subject | I | I | I | I |
|---|---|---|---|---|
| **Time Point** | **0** | **90** | **MW** | **END** |
| **metabolites** | Sample 17 | Sample 18 | Sample 19 | Sample 20 |
| Tyrosine | 150636.20 | 268322.90 | 311454.80 | 109813.30 |
| Uracil | 4311.74 | 1441.32 | 4214.14 | 1803.76 |
| Uridine | 28582.75 | 6008.29 | 17651.85 | 7607.85 |
| Urocanic acid | 583.62 | 1958.00 | 2293.04 | 1915.39 |
| Vanillin | 2362.29 | 4552.35 | 5194.46 | 3484.28 |

| Subject | K | K | K | K |
|---|---|---|---|---|
| Time Point | 0 | 90 | MW | END |
| **metabolites** | Sample 21 | Sample 22 | Sample 23 | Sample 24 |
| 1,3 Cyclohexanedione | 1673.37 | 2302.17 | 2395.47 | 2120.64 |
| 1,7-Dimethyluric acid | 525.94 | 5534.49 | 1172.02 | 1412.29 |
| 1-Methyladenosine | 2791.18 | 5892.05 | 9715.78 | 7670.52 |
| 1-Methylhistidine | 2199.55 | 1608.32 | 7925.70 | 3080.56 |
| 1-Methylnicotinamide | 378.57 | 1144.49 | 2501.05 | 865.19 |
| 2'-Deoxyguanosine 5'-monophosphate | 16152.62 | 145.77 | 142.03 | 73.69 |
| 2-Hydroxy-4-methylpentanoic acid | 8260.99 | 5219.71 | 4882.28 | 3365.13 |
| 2-Phenylacetamide | 62069.34 | 79786.70 | 91519.51 | 63743.17 |
| 3-(1-Pyrazolyl)-alanine | 176.48 | 574.77 | 499.86 | 364.25 |
| 3-(2-Hydroxyphenyl)propanoic acid | 5661.04 | 9432.93 | 7386.32 | 5197.19 |
| 3-Hydroxybenzaldehyde | 12725.24 | 17301.13 | 22612.46 | 17728.00 |
| 3-Indoleacetic acid | 19675.24 | 45134.96 | 133854.65 | 70501.70 |
| 3-Indolepropionic acid | 5322.76 | 40577.84 | 21313.24 | 21585.40 |
| 3-Methoxytyrosine | 530.07 | 640.11 | 926.52 | 791.69 |
| 4-Aminophenol | 22.99 | 60.41 | 44.92 | 52.59 |
| 4-Guanidinobutanoate | 167.98 | 180.36 | 235.17 | 264.95 |
| 4-Hydroxyhippuric acid | 53.15 | 494.54 | 56.36 | 188.41 |
| 4-Hydroxyphenylglycine | 2589.80 | 3647.79 | 5412.71 | 2840.80 |
| 4-Hydroxyproline | 1623.66 | 612.32 | 1449.36 | 661.02 |
| 4-Hydroxyquinoline | 835.40 | 1053.89 | 1565.49 | 895.90 |
| 4-Imidazoleacetate | 1278.81 | 3856.55 | 5254.01 | 4481.13 |
| 4-Methylcatechol | 356.44 | 530.19 | 854.29 | 188.28 |
| 5-Aminosalicylic acid | 22.50 | 1592.98 | 2272.80 | 914.11 |
| Acetaminophen glucuronide | 2.72 | 0.67 | 3.21 | 7.28 |
| Acetominophen | 2.63 | 10.36 | 8.73 | 9.41 |
| Acetylcholine | 25199.01 | 25086.74 | 35315.58 | 29230.40 |
| Adenosine | 442.76 | 373.59 | 377.13 | 460.99 |
| Adenosine 3',5'-Diphosphate | 12024.63 | 21.88 | 16.33 | 16.91 |
| Adenosine-3-monophosphate | 8753.34 | 42.64 | 24.98 | 9.41 |
| Adipic acid | 10243.00 | 67.59 | 54.84 | 26.23 |
| Alanine | 720.70 | 795.44 | 1205.58 | 697.24 |
| Arabinose | 18.04 | 924.38 | 1642.33 | 1072.21 |
| Arginine | 2359.01 | 5090.65 | 39867.82 | 13969.57 |
| Asp-Phe | 125.79 | 265.47 | 337.30 | 358.43 |
| Benzoic acid | 162.32 | 409.58 | 522.03 | 229.70 |
| Betaine | 102335.49 | 51097.48 | 78844.70 | 64686.88 |
| Biliverdin | 7.94 | 10379.42 | 968.28 | 2617.68 |
| Cadaverine | 648.94 | 687.12 | 1236.20 | 917.76 |
| Caffeine | 25785.88 | 590932.10 | 72351.95 | 97017.85 |
| Carnitine | 3630.17 | 6114.69 | 10245.25 | 5707.10 |
| Cholic acid | 1941.68 | 69200.16 | 22193.38 | 74796.03 |
| Choline | 126045.40 | 204002.50 | 282185.40 | 198575.20 |
| Citrulline | 3698.32 | 3540.73 | 4340.22 | 3788.11 |
| CMPF | 46571.03 | 390769.95 | 823349.25 | 354820.30 |
| Cortisol | 19051.03 | 35904.61 | 75631.10 | 56117.94 |
| Cortisone | 2471.29 | 6053.83 | 6206.91 | 6716.13 |
| Creatine | 64729.16 | 41752.82 | 60248.75 | 31171.33 |

| Subject | K | K | K | K |
|---|---|---|---|---|
| **Time Point** | **0** | **90** | **MW** | **END** |
| **metabolites** | Sample 21 | Sample 22 | Sample 23 | Sample 24 |
| Creatinine | 71804.59 | 60266.95 | 77357.30 | 65429.91 |
| Cyclo(Leu-Pro) | 3232.77 | 10928.02 | 2939.94 | 5916.63 |
| Cyclohexanamine | 757.84 | 918.40 | 1120.54 | 1454.85 |
| Cystine | 560.36 | 638.69 | 1433.04 | 806.56 |
| Dehydroisoandrosterone sulfate | 905013.45 | 1360476.50 | 1171848.00 | 996932.80 |
| delta-Trimethyllysine | 884.28 | 588.06 | 950.59 | 978.54 |
| Docosahexanoic acid | 108064.97 | 128762.54 | 63747.19 | 100777.54 |
| Dopamine | 385.73 | 447.15 | 711.35 | 512.80 |
| Eicosenoic acid | 3136.54 | 4570.39 | 1937.36 | 1087.71 |
| Ergothioneine | 9854.45 | 2418.23 | 3279.50 | 3216.38 |
| Ethanolamine | 2364.42 | 1794.37 | 2217.15 | 1840.48 |
| Ethyl myristate | 459292.90 | 505884.80 | 228497.80 | 126143.36 |
| Galactosamine | 313.65 | 238.45 | 211.28 | 258.34 |
| gamma-Glutamylleucine | 19421.55 | 29916.19 | 35767.54 | 37730.31 |
| gamma-Glutamyltyrosine | 3127.77 | 5990.68 | 5661.01 | 7073.19 |
| Glutamic acid | 3899.35 | 2143.45 | 2344.84 | 1822.38 |
| Glutamine | 7049.33 | 8980.35 | 15978.11 | 8262.80 |
| Glutarylcarnitine | 2839.09 | 2014.71 | 2267.27 | 1204.67 |
| Glu-Thr | 2570.11 | 235.85 | 645.61 | 313.57 |
| Glyceraldehyde | 194466.90 | 75475.25 | 53439.74 | 34389.04 |
| Glycochenodeoxycholate | 41706.43 | 119695.28 | 156746.90 | 84815.05 |
| Glycocholate | 6643.82 | 22230.11 | 35940.65 | 3051.02 |
| Guanosine | 41.02 | 438.10 | 33.02 | 568.54 |
| Histamine | 562.56 | 376.54 | 550.25 | 444.61 |
| Histidine | 1835.09 | 2062.33 | 6826.45 | 2562.23 |
| Homocysteine | 845.66 | 13060.54 | 17603.23 | 10109.57 |
| Hypoxanthine | 266743.00 | 42431.05 | 25726.14 | 50164.31 |
| Indole-3-carboxaldehyde | 29775.77 | 50138.94 | 64512.51 | 48669.89 |
| Indolelactic acid | 21478.83 | 19963.30 | 21089.22 | 13789.21 |
| Inosine-5-monophosphate | 7098.34 | 30.92 | 39.87 | 3.63 |
| Kynurenic acid | 764.18 | 822.96 | 1229.93 | 1102.54 |
| Kynurenine | 12250.90 | 12995.57 | 24100.07 | 13458.02 |
| Lactose | 0.00 | 2265.33 | 1590.47 | 2366.42 |
| Lauroylcarnitine | 3212.51 | 14787.37 | 8258.20 | 6175.98 |
| Leucine | 228066.40 | 278378.45 | 317406.65 | 265767.10 |
| Linoleic acid | 166763.25 | 466423.15 | 273174.55 | 253050.05 |
| Lysine | 3174.62 | 4067.53 | 5236.74 | 3927.87 |
| Mannose | 132320.28 | 453655.75 | 715688.60 | 504796.30 |
| Methionine | 16209.07 | 29502.46 | 49015.94 | 38923.88 |
| Methyl Heptadecanoic acid | 236450.05 | 119288.21 | 76820.27 | 138011.80 |
| Methylguanidine | 732.75 | 727.72 | 803.38 | 772.83 |
| Methyltestosterone | 14760.61 | 28926.81 | 12061.35 | 24110.62 |
| Methylthioadenosine | 480.06 | 1056.00 | 1239.23 | 2020.33 |
| N,N-Dimethylarginine | 28211.31 | 3364.76 | 2950.38 | 4489.18 |
| N2,N2-Dimethylguanosine | 838.57 | 1252.78 | 1594.91 | 1293.96 |
| N-Acetylglucosamine | 2.47 | 842.39 | 937.10 | 721.53 |
| N-Acetylputrescine | 596.45 | 443.31 | 658.19 | 297.68 |

| Subject | K | K | K | K |
|---:|---:|---:|---:|---:|
| **Time Point** | **0** | **90** | **MW** | **END** |
| **metabolites** | Sample 21 | Sample 22 | Sample 23 | Sample 24 |
| N-epsilon-Acetyllysine | 611.59 | 1500.81 | 1803.33 | 1391.53 |
| Nicotinamide | 9467.77 | 6205.15 | 5645.61 | 2042.13 |
| N-Methylproline | 176.49 | 1453.48 | 3898.47 | 1235.32 |
| Noradrenaline | 748.23 | 1203.04 | 1365.32 | 1716.98 |
| Norleucine | 136703.45 | 143553.50 | 196903.30 | 170871.75 |
| Norvaline | 119080.69 | 142986.60 | 202026.00 | 162251.25 |
| O-Acetylcarnitine | 107955.13 | 169658.50 | 204575.55 | 153597.40 |
| O-Acetylserine | 24670.18 | 38823.45 | 32572.19 | 39793.32 |
| Octopine | 317.41 | 710.38 | 1130.21 | 736.57 |
| Olmesartan | 25.91 | 44.89 | 20.92 | 16.73 |
| Ornithine | 3257.45 | 2788.76 | 2331.54 | 1997.43 |
| Palmitoylcarnitine | 3949.49 | 12225.01 | 11823.77 | 6955.16 |
| Pantothenic acid | 1141.39 | 5399.37 | 11060.59 | 5643.15 |
| Paraxanthine | 48676.92 | 405036.15 | 101688.04 | 122592.45 |
| Phenylacetaldehyde | 387.69 | 732.08 | 699.41 | 817.77 |
| Phenylacetylglutamine | 68692.06 | 53719.20 | 131197.15 | 99802.96 |
| Phenylalanine | 530115.05 | 817379.80 | 912279.45 | 740254.70 |
| Phenylethanolamine | 158.98 | 439.97 | 399.95 | 463.85 |
| Phosphocholine | 82527.53 | 40804.14 | 63142.72 | 68916.28 |
| Phosphoric acid | 14230.79 | 30803.09 | 34368.05 | 28204.09 |
| P-Hydroxyphenylacetate | 338.39 | 288.96 | 656.28 | 546.58 |
| Pipecolate | 15486.74 | 13972.61 | 18865.12 | 14264.37 |
| Piperine | 6435.53 | 1780.63 | 13191.07 | 18384.34 |
| Proline | 159500.87 | 143739.80 | 146415.00 | 146210.55 |
| Propanoate | 2871.36 | 11916.66 | 14611.44 | 13107.98 |
| Propionylcarnitine | 18093.67 | 18779.51 | 19524.13 | 7222.00 |
| Putrescine | 769.57 | 713.77 | 655.34 | 697.74 |
| Pyridoxamine | 1791.52 | 2370.84 | 2902.78 | 2130.04 |
| Pyroglutamic acid | 14512.00 | 17279.35 | 26971.19 | 14587.59 |
| Quinoline | 15103.30 | 32156.87 | 100549.49 | 53088.08 |
| Resorcinol Monoacetate | 3321.81 | 6045.96 | 6372.23 | 6026.40 |
| Riboflavin | 374.10 | 584.08 | 774.67 | 767.81 |
| Ribose 5-Phosphate | 0.00 | 2502.08 | 4935.18 | 3577.02 |
| Sedoheptulose | 996.16 | 152.06 | 159.49 | 118.71 |
| Serine | 492.94 | 769.45 | 743.36 | 680.19 |
| Spermine | 196.44 | 86.02 | 301.80 | 306.13 |
| Taurine | 5358.46 | 3438.13 | 4715.92 | 3884.26 |
| Theobromine | 49114.95 | 295815.00 | 246850.70 | 181447.50 |
| Theophylline | 6069.89 | 76010.76 | 16518.28 | 22429.09 |
| Thiazolidine-4-carboxylic acid | 4813.73 | 3001.11 | 3997.27 | 4112.13 |
| Threitol | 1204.52 | 2270.01 | 2801.00 | 2265.04 |
| Threonic acid | 2167.97 | 11074.82 | 13010.19 | 6522.42 |
| Threonine | 1538.81 | 1223.88 | 1484.44 | 1236.40 |
| Trigonelline | 3663.61 | 10791.32 | 6601.58 | 9532.77 |
| Trimethylamine N-oxide | 1712.77 | 5515.16 | 3197.83 | 3280.64 |
| Tryptamine | 18341.59 | 31361.38 | 38604.51 | 31982.09 |
| Tryptophan | 1073420.20 | 1895982.50 | 2369759.50 | 1980387.50 |

| Subject | K | K | K | K |
|---|---|---|---|---|
| **Time Point** | **0** | **90** | **MW** | **END** |
| **metabolites** | Sample 21 | Sample 22 | Sample 23 | Sample 24 |
| Tyrosine | 161484.95 | 231971.40 | 244835.60 | 163368.95 |
| Uracil | 6483.08 | 4697.52 | 4898.93 | 2070.87 |
| Uridine | 39968.76 | 21579.66 | 19329.03 | 8233.29 |
| Urocanic acid | 952.52 | 1892.44 | 2412.77 | 2188.80 |
| Vanillin | 2944.87 | 3733.20 | 3784.49 | 4357.33 |

| Subject | O | O | O | O |
|---|---|---|---|---|
| Time Point | 0 | 90 | MW | END |
| **metabolites** | Sample 25 | Sample 26 | Sample 27 | Sample 28 |
| 1,3 Cyclohexanedione | 1681.63 | 1182.35 | 3258.18 | 2253.81 |
| 1,7-Dimethyluric acid | 1797.42 | 353.69 | 3558.79 | 703.25 |
| 1-Methyladenosine | 1815.29 | 946.80 | 6176.03 | 7127.11 |
| 1-Methylhistidine | 3798.44 | 2569.14 | 3815.93 | 4645.13 |
| 1-Methylnicotinamide | 96.83 | 1759.31 | 2550.28 | 2000.49 |
| 2'-Deoxyguanosine 5'-monophosphate | 12461.59 | 141.82 | 90.70 | 127.94 |
| 2-Hydroxy-4-methylpentanoic acid | 8674.83 | 4605.60 | 11321.66 | 8104.24 |
| 2-Phenylacetamide | 60281.01 | 96520.96 | 82916.16 | 73910.63 |
| 3-(1-Pyrazolyl)-alanine | 60.24 | 395.74 | 360.54 | 450.01 |
| 3-(2-Hydroxyphenyl)propanoic acid | 8293.40 | 5444.08 | 7458.87 | 9801.25 |
| 3-Hydroxybenzaldehyde | 12301.62 | 20583.80 | 9341.94 | 16055.82 |
| 3-Indoleacetic acid | 23292.30 | 21401.19 | 38944.25 | 28065.22 |
| 3-Indolepropionic acid | 14375.00 | 3318.05 | 9550.19 | 12076.73 |
| 3-Methoxytyrosine | 371.61 | 73.58 | 996.29 | 983.52 |
| 4-Aminophenol | 16.37 | 7.06 | 11089.15 | 71.71 |
| 4-Guanidinobutanoate | 82.64 | 355.05 | 142.72 | 212.44 |
| 4-Hydroxyhippuric acid | 47.27 | 64.63 | 34.74 | 47.51 |
| 4-Hydroxyphenylglycine | 3236.78 | 4998.43 | 5646.19 | 5026.33 |
| 4-Hydroxyproline | 1766.64 | 932.39 | 1116.19 | 806.60 |
| 4-Hydroxyquinoline | 754.00 | 109.51 | 1889.11 | 1684.01 |
| 4-Imidazoleacetate | 1325.91 | 3116.58 | 2974.05 | 5171.85 |
| 4-Methylcatechol | 196.48 | 759.44 | 350.04 | 228.26 |
| 5-Aminosalicylic acid | 31.98 | 1350.33 | 831.57 | 943.36 |
| Acetaminophen glucuronide | 6.08 | 0.00 | 9124.16 | 18.24 |
| Acetominophen | 5.27 | 11.16 | 11126.77 | 6.27 |
| Acetylcholine | 14798.04 | 41615.27 | 22508.63 | 37579.53 |
| Adenosine | 440.32 | 25.45 | 327.67 | 531.08 |
| Adenosine 3',5'-Diphosphate | 5551.12 | 18.81 | 17.39 | 27.78 |
| Adenosine-3-monophosphate | 5023.98 | 18.29 | 17.78 | 46.33 |
| Adipic acid | 8146.87 | 16951.51 | 30.34 | 44.96 |
| Alanine | 1133.13 | 568.25 | 502.11 | 896.19 |
| Arabinose | 15.70 | 1017.10 | 1164.58 | 1428.05 |
| Arginine | 5034.53 | 16646.35 | 25477.34 | 15816.51 |
| Asp-Phe | 130.69 | 74.03 | 324.41 | 463.80 |
| Benzoic acid | 592.28 | 98.21 | 165.23 | 71.04 |
| Betaine | 66365.47 | 52166.47 | 46668.25 | 104394.00 |
| Biliverdin | 6.23 | 3.23 | 419.21 | 15721.04 |
| Cadaverine | 489.97 | 1748.22 | 874.52 | 1363.36 |
| Caffeine | 170219.10 | 51349.42 | 660484.30 | 99126.52 |
| Carnitine | 3505.89 | 4792.26 | 4471.36 | 4683.74 |
| Cholic acid | 36461.67 | 143893.60 | 9445.08 | 43554.42 |
| Choline | 106155.03 | 164487.90 | 164931.70 | 229252.05 |
| Citrulline | 3015.63 | 1708.02 | 2087.20 | 4120.54 |
| CMPF | 70794.71 | 178360.20 | 310250.20 | 254318.90 |
| Cortisol | 23503.22 | 35824.85 | 100986.10 | 72353.36 |
| Cortisone | 4705.39 | 2377.11 | 5631.55 | 5180.51 |
| Creatine | 53830.49 | 29988.47 | 19810.11 | 34851.99 |

| Subject | O | O | O | O |
|---|---|---|---|---|
| **Time Point** | **0** | **90** | **MW** | **END** |
| **metabolites** | Sample 25 | Sample 26 | Sample 27 | Sample 28 |
| Creatinine | 45586.13 | 79511.59 | 66216.23 | 94750.74 |
| Cyclo(Leu-Pro) | 3908.36 | 4528.43 | 17129.47 | 7892.86 |
| Cyclohexanamine | 346.48 | 220.77 | 1206.09 | 2106.28 |
| Cystine | 540.23 | 630.90 | 632.80 | 925.61 |
| Dehydroisoandrosterone sulfate | 1256409.75 | 1981525.00 | 2920496.00 | 2620868.00 |
| delta-Trimethyllysine | 1357.83 | 1657.61 | 920.04 | 1359.40 |
| Docosahexanoic acid | 85838.02 | 72456.76 | 37228.51 | 70697.74 |
| Dopamine | 334.18 | 43.46 | 677.68 | 492.74 |
| Eicosenoic acid | 4326.13 | 826.24 | 2331.53 | 3799.93 |
| Ergothioneine | 7751.43 | 1517.39 | 1511.87 | 1818.80 |
| Ethanolamine | 1901.91 | 1883.39 | 1608.01 | 2076.30 |
| Ethyl myristate | 460374.05 | 210668.60 | 299144.30 | 504243.65 |
| Galactosamine | 318.27 | 290.92 | 195.93 | 253.64 |
| gamma-Glutamylleucine | 12705.81 | 23437.57 | 52432.97 | 49935.59 |
| gamma-Glutamyltyrosine | 2524.69 | 2086.27 | 5899.78 | 6353.69 |
| Glutamic acid | 3044.67 | 967.87 | 1063.90 | 1693.81 |
| Glutamine | 7707.82 | 11198.85 | 13466.68 | 16993.01 |
| Glutarylcarnitine | 2661.75 | 368.11 | 2377.86 | 2351.50 |
| Glu-Thr | 3445.50 | 416.19 | 350.61 | 414.05 |
| Glyceraldehyde | 165104.90 | 49554.04 | 26065.31 | 44248.28 |
| Glycochenodeoxycholate | 80885.94 | 336916.10 | 257461.00 | 160757.85 |
| Glycocholate | 7896.46 | 26100.14 | 14165.42 | 15399.03 |
| Guanosine | 27.47 | 10.91 | 43.38 | 380.90 |
| Histamine | 452.12 | 518.84 | 521.32 | 433.48 |
| Histidine | 3687.33 | 2788.99 | 4775.23 | 4816.22 |
| Homocysteine | 429.76 | 17174.39 | 10533.33 | 13924.56 |
| Hypoxanthine | 470014.00 | 6597.87 | 9511.73 | 73627.25 |
| Indole-3-carboxaldehyde | 24235.54 | 21834.18 | 65599.73 | 62621.73 |
| Indolelactic acid | 13504.54 | 18320.73 | 32744.54 | 25032.05 |
| Inosine-5-monophosphate | 2764.32 | 51.73 | 79.96 | 55.59 |
| Kynurenic acid | 682.19 | 1436.69 | 2429.50 | 1165.29 |
| Kynurenine | 8876.13 | 1360.21 | 30670.89 | 20330.90 |
| Lactose | 0.00 | 5798.03 | 2179.80 | 1768.86 |
| Lauroylcarnitine | 5041.64 | 9013.25 | 31525.17 | 32901.76 |
| Leucine | 216889.50 | 257672.90 | 302308.00 | 308185.45 |
| Linoleic acid | 251659.25 | 81606.99 | 213024.60 | 341653.25 |
| Lysine | 3196.68 | 5133.49 | 4401.02 | 4536.88 |
| Mannose | 138665.39 | 549125.65 | 422406.70 | 548318.40 |
| Methionine | 12925.39 | 36821.22 | 31762.09 | 42122.38 |
| Methyl Heptadecanoic acid | 186216.25 | 102092.89 | 82784.36 | 119504.69 |
| Methylguanidine | 680.42 | 976.67 | 448.31 | 1032.87 |
| Methyltestosterone | 20072.09 | 6426.56 | 31806.02 | 27458.21 |
| Methylthioadenosine | 347.63 | 482.63 | 1260.83 | 1156.40 |
| N,N-Dimethylarginine | 18394.56 | 2535.04 | 2189.16 | 3237.58 |
| N2,N2-Dimethylguanosine | 714.41 | 399.65 | 1693.71 | 1339.68 |
| N-Acetylglucosamine | 14.59 | 717.86 | 634.81 | 884.41 |
| N-Acetylputrescine | 508.28 | 468.18 | 364.05 | 335.96 |

| Subject | O | O | O | O |
|---|---|---|---|---|
| **Time Point** | **0** | **90** | **MW** | **END** |
| **metabolites** | Sample 25 | Sample 26 | Sample 27 | Sample 28 |
| N-epsilon-Acetyllysine | 764.27 | 965.87 | 1261.00 | 1283.45 |
| Nicotinamide | 6752.36 | 2655.79 | 3129.11 | 2557.10 |
| N-Methylproline | 143.14 | 2751.06 | 2391.78 | 3629.48 |
| Noradrenaline | 586.52 | 1517.91 | 1612.71 | 1668.32 |
| Norleucine | 128832.75 | 184466.30 | 176931.50 | 194381.90 |
| Norvaline | 104611.95 | 199761.05 | 127775.10 | 196912.80 |
| O-Acetylcarnitine | 146684.55 | 134276.35 | 164724.30 | 214094.55 |
| O-Acetylserine | 30086.94 | 27604.70 | 33023.29 | 40708.07 |
| Octopine | 426.22 | 512.12 | 674.69 | 528.40 |
| Olmesartan | 24.86 | 16.87 | 26.78 | 42.96 |
| Ornithine | 2374.44 | 2534.60 | 1831.93 | 2228.61 |
| Palmitoylcarnitine | 3489.88 | 2290.80 | 21371.63 | 19477.37 |
| Pantothenic acid | 1488.58 | 266.57 | 2100.03 | 2064.72 |
| Paraxanthine | 232925.55 | 21380.33 | 313274.70 | 54495.94 |
| Phenylacetaldehyde | 639.94 | 1172.24 | 445.71 | 511.54 |
| Phenylacetylglutamine | 14385.92 | 18492.65 | 77964.36 | 50062.72 |
| Phenylalanine | 483998.50 | 2182.90 | 801536.40 | 825609.60 |
| Phenylethanolamine | 158.58 | 196.77 | 469.38 | 413.90 |
| Phosphocholine | 83794.56 | 74976.32 | 73945.49 | 70811.14 |
| Phosphoric acid | 12095.00 | 36390.68 | 30310.23 | 37480.72 |
| P-Hydroxyphenylacetate | 171.07 | 385.75 | 408.63 | 452.69 |
| Pipecolate | 12671.31 | 16470.72 | 12339.74 | 15761.39 |
| Piperine | 7679.25 | 3709.61 | 12651.22 | 27318.10 |
| Proline | 102677.69 | 298605.90 | 101270.20 | 178396.50 |
| Propanoate | 2873.13 | 10534.87 | 9480.07 | 15695.86 |
| Propionylcarnitine | 12305.73 | 4336.57 | 6735.26 | 5391.55 |
| Putrescine | 687.20 | 1049.25 | 629.92 | 830.11 |
| Pyridoxamine | 2264.94 | 2726.12 | 2193.46 | 2589.07 |
| Pyroglutamic acid | 14290.87 | 17953.23 | 23232.62 | 29820.01 |
| Quinoline | 17179.88 | 15576.52 | 28881.14 | 22043.55 |
| Resorcinol Monoacetate | 2886.61 | 2106.73 | 7214.24 | 6098.69 |
| Riboflavin | 291.48 | 417.97 | 779.71 | 655.12 |
| Ribose 5-Phosphate | 74.27 | 4368.35 | 3168.25 | 6182.50 |
| Sedoheptulose | 488.60 | 128.81 | 104.47 | 185.37 |
| Serine | 641.22 | 890.63 | 536.54 | 746.89 |
| Spermine | 242.68 | 116.82 | 269.24 | 371.35 |
| Taurine | 4210.98 | 1879.05 | 3211.94 | 4489.75 |
| Theobromine | 93455.70 | 156063.15 | 734313.00 | 178838.85 |
| Theophylline | 41858.98 | 3448.92 | 52722.62 | 8883.32 |
| Thiazolidine-4-carboxylic acid | 5151.01 | 4552.11 | 2723.71 | 5558.41 |
| Threitol | 911.79 | 2044.83 | 1390.13 | 2360.43 |
| Threonic acid | 852.90 | 3429.55 | 13691.66 | 17329.32 |
| Threonine | 1647.65 | 1363.81 | 873.97 | 1345.87 |
| Trigonelline | 7820.35 | 12331.28 | 3177.00 | 13645.55 |
| Trimethylamine N-oxide | 1122.60 | 2103.20 | 2183.72 | 7526.18 |
| Tryptamine | 15767.24 | 15020.46 | 40121.29 | 38999.18 |
| Tryptophan | 971256.10 | 879541.10 | 2335039.00 | 2314164.00 |

| Subject | O | O | O | O |
|---|---|---|---|---|
| **Time Point** | **0** | **90** | **MW** | **END** |
| **metabolites** | Sample 25 | Sample 26 | Sample 27 | Sample 28 |
| Tyrosine | 158368.65 | 275753.25 | 221368.70 | 187604.25 |
| Uracil | 5803.93 | 1099.35 | 4815.21 | 3605.79 |
| Uridine | 32717.96 | 3387.89 | 11499.75 | 13573.81 |
| Urocanic acid | 578.44 | 2517.09 | 2071.88 | 1675.33 |
| Vanillin | 2559.52 | 1501.21 | 4834.63 | 4049.55 |
